# Hirshfeld atom refinement

**DOI:** 10.1107/S2052252514014845

**Published:** 2014-08-29

**Authors:** Silvia C. Capelli, Hans-Beat Bürgi, Birger Dittrich, Simon Grabowsky, Dylan Jayatilaka

**Affiliations:** a Department of Chemistry, University of Milan, Via Golgi 19, 20133 Milan, Italy; b Department of Chemistry and Biochemistry, Universität Bern, Freiestrasse 3, 3012 Bern, Switzerland; c Department of Chemistry, Universität Zürich, Winterthurstrasse 190, 8057 Zürich, Switzerland; d Institut für Anorganische und Angewandte Chemie, Universität Hamburg, Martin-Luther-King-Platz 6, 20146 Hamburg, Germany; e Institut für Anorganische Chemie, Georg-August-Universität Göttingen, Tammannstr. 4, 37077 Göttingen, Germany; f School of Chemistry and Biochemistry, The University of Western Australia, 35 Stirling Highway, Crawley WA 6009, Australia

**Keywords:** aspherical atom partitioning, quantum mechanical molecular electron densities, X-ray structure refinement, hydrogen atom modelling, anisotropic displacement parameters

## Abstract

Hirshfeld atom refinement (HAR) is a method which determines structural parameters from single-crystal X-ray diffraction data by using an aspherical atom partitioning of tailor-made *ab initio* quantum mechanical molecular electron densities without any further approximation. Here the original HAR method is extended by implementing an iterative procedure of successive cycles of electron density calculations, Hirshfeld atom scattering factor calculations and structural least-squares refinements, repeated until convergence. The importance of this iterative procedure is illustrated *via* the example of crystalline ammonia. The new HAR method is then applied to X-ray diffraction data of the dipeptide Gly–l-Ala measured at 12, 50, 100, 150, 220 and 295 K, using Hartree–Fock and BLYP density functional theory electron densities and three different basis sets. All positions and anisotropic displacement parameters (ADPs) are freely refined without constraints or restraints – even those for hydrogen atoms. The results are systematically compared with those from neutron diffraction experiments at the temperatures 12, 50, 150 and 295 K. Although non-hydrogen-atom ADPs differ by up to three combined standard uncertainties (csu’s), all other structural parameters agree within less than 2 csu’s. Using our best calculations (BLYP/cc-pVTZ, recommended for organic molecules), the accuracy of determining bond lengths involving hydrogen atoms from HAR is better than 0.009 Å for temperatures of 150 K or below; for hydrogen-atom ADPs it is better than 0.006 Å^2^ as judged from the mean absolute X-ray minus neutron differences. These results are among the best ever obtained. Remarkably, the precision of determining bond lengths and ADPs for the hydrogen atoms from the HAR procedure is comparable with that from the neutron measurements – an outcome which is obtained with a routinely achievable resolution of the X-ray data of 0.65 Å.

## Abstract 2

The new automated iterative Hirshfeld atom refinement method is explained and validated through comparison of structural models of Gly–l-Ala obtained from synchrotron X-ray and neutron diffraction data at 12, 50, 150 and 295 K. Structural parameters involving hydrogen atoms are determined with comparable precision from both experiments and agree mostly to within two combined standard uncertainties.

## 1. Introduction   

Structure and reactivity are the essence of chemistry, and X-ray diffraction is the work-horse for structure determination (Giacovazzo, 2002 ▶; Dunitz, 1995 ▶). For crystals, the structure is described by unit-cell dimensions, time- and space-averaged atomic positions in the unit cell and the corres­ponding mean-squared displacements, represented by the so-called atomic displacement tensors (Trueblood *et al.*, 1996 ▶). Cell parameters, coordinates and displacement tensors are gleaned from the signal generated when X-rays diffract from the electronic and nuclear charge density in the molecule. The overwhelming part of the signal is due to the electron density (ED), the maxima of which serve as proxies for the nuclear positions.

The structural data pertaining to H atoms, which form the skin of most organic and organometallic molecules, are particularly important in many fields such as enzymology (Halliwell & Gutteridge, 1999 ▶), organic reaction mechanisms (Hynes *et al.*, 2007 ▶), supramolecular chemistry and crystal engineering (Desiraju, 1989 ▶; Desiraju & Steiner, 1999 ▶). From the point of view of X-rays, the H atom is just a small hump in the landscape of the total electron density. Neutrons ‘see’ H atoms more clearly than X-rays; diffraction of neutrons has the advantage of providing atomic positions and anisotropic displacement parameters (ADPs) for H atoms as accurately as for other atom types. Therefore, considerable technical and financial effort has been made to construct beamlines at neutron facilities, *e.g.* new spallation sources, in order to secure H-atom parameters (Langan *et al.*, 2004 ▶, 2008 ▶; Myles, 2006 ▶; Bunick & Hanson, 2003 ▶). However, X-ray diffraction is the much more widespread technique to study crystalline materials. The enormous progress in the development and availability of in-house and synchrotron sources, detectors and software has transformed X-ray analysis into a standard investigation tool that can be easily accessed by an increasing number of users. In addition, the ability of X-rays to probe the electronic distribution in a solid, a property directly related to chemical bonding and macroscopic behaviour, has made X-ray electron density analysis an important tool for studying crystals.

Our interest here is in high-quality X-ray structural data, those related to H atoms in particular. Apart from the usual intrinsic interest in advancing any experimental technique, the ability to obtain accurate and precise structural data is a critical prerequisite for several types of secondary analyses:

(i) The first is the analysis of the electron density obtained from accurate X-ray diffraction data (Tsirelon & Ozerov, 1996 ▶; Coppens, 1997 ▶; Gatti & Macchi, 2012 ▶; Stalke, 2012 ▶). The importance of the positions and ADPs in this field is discussed in more detail in §2. Electron density analysis addresses the fundamentals of chemical bonding, which gives rise to the potential surfaces on which nuclear dynamics and chemical reactions occur.
(ii) The second example is ‘normal coordinate analysis’, whereby ADPs are analyzed at several temperatures in order to obtain the low-frequency normal modes of a molecule (Bürgi & Capelli, 2000 ▶; Capelli *et al.*, 2000 ▶; Bürgi *et al.*, 2000 ▶). This kind of analysis goes some way toward understanding the nuclear dynamics of molecular systems in real space and complements spectroscopy, which works in the energy regime.

Given the basic importance of the X-ray structural data in itself, and for further analyses, and given that the X-ray technique is now a century old, it seems timely to ask: how accurately and precisely can we obtain these data now? The IUCr project on α-oxalic acid was an important milestone which established that quantitative agreement can be obtained for non-H-atom positions between different measurements provided high-angle reflection data are used. The positions were determined to a precision of 0.001 Å (Coppens *et al.*, 1983 ▶). However, H-atom positions were not compared in this study. A later study of *syn*-1,6:8,13-biscarbonyl[14]annulene at 19 K by Destro & Merati (1995 ▶) reported a precision in the positions of better than 0.0004 Å for non-H atoms and of ∼0.007 Å for H atoms. The average of the ten aromatic C—H bond lengths was found to be 1.087 (7) Å, indistinguishable from the average of neutron determined C_ar_—H distances of 1.083 (11) Å (Allen *et al.*, 2004 ▶). This result was achieved by assuming generalized structure factors from ‘polarized’ H atoms, taken from the hydrogen molecule and including monopole and dipole terms (Stewart *et al.*, 1975 ▶; Coppens *et al.*, 1971 ▶). Two similar studies by Zhurov *et al.* (2011 ▶) modelled the electron density on the H atoms with a monopole, three dipoles and one quadrupole (*Q*_0_). For hexahydro-1,3,5-trinitro-1,3,5-triazine, the average of six methylene C—H bond distances was found to be 1.073 (12) Å at 20 K and 1.082 (15) Å at 298 K, to be compared with the corresponding room-temperature neutron result of 1.082 (12) Å (Choi & Prince, 1972 ▶) and the average methylene C—H neutron distances of 1.097 (10) Å at *T* ≤ 60 K and 1.087 (16) Å at *T* ≥ 240 K (Allen & Bruno, 2010 ▶). For the ferroelectric croconic acid (Zhurov & Pinkerton, 2013 ▶) the average of the two O—H distances is 0.941 Å, to be compared with an average neutron value for C_ar_O—H groups of 0.992 (17) Å (Allen & Bruno, 2010 ▶). In the above studies, there seems to be a tendency for the bonds involving an H atom (*D*—H) to be shorter if determined from X-ray diffraction than the distances from neutron diffraction by up to 0.05 Å. However, in all cases the difference is clearly less than the bond shortening of 0.1 Å usually found after spherical atom refinements.

As a result of many years of electron density analysis, several libraries of experimental and theoretical generalized X-ray scattering factors have been established using non-spherical pseudo-atoms (Dittrich *et al.*, 2013 ▶; Volkov *et al.*, 2004 ▶; Jarzembska & Dominiak, 2012 ▶; Domagała *et al.*, 2012 ▶; Hathwar *et al.*, 2011 ▶). This idea was pioneered by Stewart *et al.* (1975 ▶) to obtain improved time-averaged proton positions. A few systematic comparisons between results from applications of databases to X-ray data and results from neutron diffraction have been published (Dittrich *et al.*, 2005 ▶, 2009 ▶; Bendeif & Jelsch, 2007 ▶; Bąk *et al.*, 2011 ▶; Dadda *et al.*, 2012 ▶). Agreement of *D*—H distances with values from neutron diffraction is improved relative to that obtained from spherical atom refinements. Depending on the database, *D*—H distances generally differ by a few hundredths of an Ångström compared with (tabulated) bond distances derived from neutron experiments.

It has been known for a long time that refinement against high-angle X-ray data improves the description of atomic displacements (Hirshfeld, 1976 ▶). Subsequently, Blessing (1995 ▶) had noticed that the differences between non-H ADPs determined by X-ray and neutron diffraction can be minimized by using high-angle data together with multipoles in X-ray refinements. Where significant differences persisted, he proposed empirical corrections to adjust X-ray ADPs to those obtained from neutron diffraction. To our knowledge, Iversen *et al.* (1996 ▶) were the first to demonstrate explicitly that, if low temperatures are used and if one is careful with the data analysis, then quantitative agreement between X-ray and neutron diffraction ADPs can be achieved for non-H atoms. In a recent update of this work similar conclusions were reached (Morgenroth *et al.*, 2008 ▶).

It has been recognized that not only H-atom positions but also the corresponding ADPs are critical prerequisites for obtaining accurate electron densities and derived properties (Spackman *et al.*, 2007 ▶; Madsen *et al.*, 2004 ▶; Hoser *et al.*, 2009 ▶). In the case of the [14]annulene mentioned above, H-atom ADPs were estimated from a TLS (translation/libration/screw coupling) analysis of the C atoms, supplemented with values estimated from spectroscopy for the C—H stretch and bend motions (Destro & Merati, 1995 ▶). Subsequently, several different and increasingly sophisticated methods for approximating H-atom ADPs have been proposed. These methods are well reviewed in the articles by Munshi *et al.* (2008 ▶) and Madsen (2012 ▶). The former propose using a modification of Madsen’s database method (Madsen, 2006 ▶) to construct H-atom ADPs from neutron data, whereas Madsen *et al.* currently explore pure *ab initio* techniques for the determination of H-atom ADPs (Madsen *et al.*, 2013 ▶).

The philosophy behind the methods for approximating H-atom ADPs is based on a statement by Hirshfeld: ‘*…there is no possibility of deriving hydrogen vibration parameters from the X-ray intensities*’ (Hirshfeld, 1976 ▶; italics in the original text). Hirshfeld’s verdict voices the unfortunate fact that, in a least-squares determination, the ADPs are highly correlated with the model used to represent the electron density (O’Connell *et al.*, 1966 ▶; El Haouzi *et al.*, 1996 ▶). At ambient or higher temperatures the ADPs often inappropriately ameliorate agreement factors by absorbing other physical effects, such as vibrational anharmonicity and effects related to thermal diffuse scattering (Willis & Pryor, 1975 ▶). In this context the results found for the above-mentioned hexahydro-1,3,5-trinitro-1,3,5-triazine at 298 K are remarkable (Zhurov *et al.*, 2011 ▶). The average difference between the H-atom ADPs from the room-temperature X-ray data and from the neutron data is 2.3 su’s. The corresponding numbers for O, N and C are 1.5, 1.2 and 1.1 su’s, respectively.

Jayatilaka & Dittrich (2008 ▶) have demonstrated that it *is* possible to obtain H-atom positions that are in quantitative agreement with positions from neutron diffraction, and H-atom ADPs that are nearly so, using high-resolution X-ray data sets of urea and benzene. This was achieved using Hirshfeld’s stockholder partitioning scheme (Hirshfeld, 1977 ▶) to obtain aspherical atomic electron densities and their scattering factors from *ab initio* self-consistent charge-embedded mol­ecular electron density calculations. With these scattering factors, atomic coordinates and ADPs were refined against the structure-factor amplitudes in the usual way. This method is colloquially known as ‘Hirshfeld atom refinement’ (HAR).

Despite this success there are a number of issues with the earlier version of HAR proposed by Jayatilaka & Dittrich (2008 ▶):

(i) For efficiency reasons, Jayatilaka & Dittrich (2008 ▶) used a ‘rigid atom approximation’, whereby the Hirshfeld atoms were determined once and then held fixed during the X-ray refinement procedure.^1^ The effect of this approximation was not properly explored. It can be removed by repeated cycles of electron density and non-spherical atomic scattering factor calculations followed by coordinate and ADP refinements. In the previous work only one such cycle was calculated and the refinements were not checked for convergence. Furthermore, it is important to quantify and minimize the effort involved in the *ab initio* calculation of the electron density because this step is much more time-consuming compared with the X-ray structure refinements. The dangers inherent in an incomplete HAR-like refinement will be illustrated in §2.

(ii) Urea and benzene are rather small and symmetrical molecules. This leads to two questions. Firstly, are the results for such molecules representative of those expected for larger molecules without inherent symmetry? Secondly, which type of quantum mechanical calculation and which basis set are necessary and adequate for dealing with larger molecules?

(iii) The data sets of urea (Birkedal *et al.*, 2004 ▶) and benzene (Bürgi *et al.*, 2002 ▶), as well as all other data sets of compounds that have been subjected to HAR in different studies for different purposes (Dittrich *et al.*, 2012 ▶; Dittrich & Jayatilaka, 2012 ▶; Chęcińska *et al.*, 2013 ▶; Grabowsky *et al.*, 2012 ▶), are high-resolution data sets (*d* < 0.5 Å) originally collected for experimental electron density studies. It is unclear to what extent the use of lower-resolution data affects the accuracy of hydrogen and non-hydrogen parameters within the framework of HAR.

(iv) The X-ray HAR ADPs of benzene C_6_H_6_ were compared with ADPs obtained indirectly from neutron ADPs of deuterobenzene C_6_D_6_ by taking isotope effects into account and interpolating between different experimental temperatures *via* a normal coordinate analysis (Bürgi *et al.*, 2002 ▶). There are lingering doubts about the validity of this comparison, and direct comparison between X-ray HAR and neutron ADPs at the same temperature for the same compound would be necessary to dispel those doubts.

To address these concerns we have done the following:

(i) We have removed the rigid atom approximation by implementing an automated procedure where molecular calculations and structure refinements alternate and are iterated to convergence. This new version of HAR is described in §3.3.

(ii) We have measured X-ray diffraction data of the dipeptide Gly–l-Ala at six different temperatures (12, 50, 100, 150, 220 and 295 K) with synchrotron radiation. At four of these temperatures (12, 50, 150 and 295 K) neutron diffraction data are available (Capelli *et al.*, 2014 ▶). In its crystalline form this molecule, the structure of which was first determined by Wang & Paul (1979 ▶), has no symmetry; it has 20 atoms and is thus larger than either urea or benzene studied previously; half of the atoms are H atoms.

(iii) We have performed the new HAR procedure for Gly–l-Ala at all temperatures with different quantum-mechanical methods and basis sets. This permitted a direct comparison between the atomic coordinates, ADPs and molecular geometries from the X-ray and neutron diffraction studies. In order to permit a more rigorous statistical analysis, uncertainties in these quantities were obtained from the full variance–covariance matrix, whereas in earlier versions of HAR they had to be estimated.

## 2. An example concerning the importance of accurate geometries and ADPs   

Bytheway *et al.* (2002 ▶) have published a study on the ammonia molecule. They modified an isolated molecule Hartree–Fock wavefunction and thus the molecular electron density to reproduce observed X-ray structure factors to a χ^2^ value of 1 (starting from an unconstrained value of 10.5) and with the minimum possible electronic energy penalty [so-called X-ray constrained wavefunction (XCW) fitting (Jayatilaka, 1998 ▶; Jayatilaka & Grimwood, 2001 ▶)]. This study used the unmodified atomic coordinates and ADPs determined by Boese *et al.* (1997 ▶) from a multipole refinement using 160 K X-ray data. H-atom coordinates were corrected to match approximately the N—H distance obtained from neutron powder data at 2 K [1.012 (2) Å; Hewat & Riekel, 1979 ▶] and an isotropic hydrogen displacement parameter was fixed at the value from an independent atom refinement (Table 1 ▶).

Given the uncertainties related to the interdependence of electron densities, accurate geometries and ADPs (see §1), Boese’s diffraction data and starting geometry have now been used for an iterative HAR (see §3.3) using the same level of theory as in the preceding study by Bytheway *et al.* (2002 ▶) [HF/6-311++G(2d,2p)]. This calculation reveals significant changes in the H-atom coordinates and ADPs (Table 1 ▶). Crystallographic data for this refinement are given as supporting information, also available from Fachinformationszentrum Karlsruhe as CIF *via* www.fiz-karlsruhe.de/request_for_deposited_data.html.

After six iterations of alternating between electron density calculations and crystallographic least-squares refinements, the parameter shifts divided by the su’s of the parameters were smaller than 0.01 and the χ^2^ statistic became 0.6. This is much less than the starting value χ^2^ = 10.5 and less than the target value χ^2^ = 1 assumed in the constrained wavefunction fit using the structural parameters from Boese *et al.* (1997 ▶). This decrease is due only to the changes in atomic coordinates and ADPs; and because Hirshfeld atoms are used to perform the thermal smearing. The H-atom parameters change more than those of the N atom, and the changes seem reasonable. The refined N—H bond length at 160 K [0.987 (5) Å] exactly matches the average neutron value of 0.989 (5) Å at 180 K. This is, however, incidental to the main point: refinement of the structural parameters produces a goodness of fit less than 1 by adjusting atomic coordinates and ADPs, but without having to tamper with the electronic wavefunction. In fact, if the experimental errors are to be believed, there is nothing to constrain after refining the structural parameters!

This finding presents a striking example of the importance of the positions and ADPs for the electron density. As a result of this new analysis, it is possible that the reconstructed electron densities in the 2002 paper – although reproducible – contain artefacts due to the use of sub-optimal geometric parameters. Analogous iterative HARs for urea and benzene did not change the single-cycle results significantly.

Rather than pursuing the case of crystalline ammonia, another small and symmetric molecule, we now turn to analysing the results from the larger and unsymmetric Gly–l-Ala molecule.

## 3. Methodology   

### 3.1. Neutron data collection   

Details of data collection, data processing and structure refinement for the neutron diffraction experiments on Gly–l-Ala at 12, 50, 150 and 295 K are given elsewhere (Capelli *et al.*, 2014 ▶). For the sake of easy direct comparison with the X-ray data, we reproduce some relevant details of the experiments in Table 2 ▶.

### 3.2. Synchrotron X-ray data collection and preliminary data analysis   

Colourless prismatic crystals of Gly–l-Ala were grown by slow evaporation from aqueous solution. Intensities of X-ray reflections were collected at 12, 50, 100, 150, 220 and 295 K with a Bruker SMART CCD detector on the ID11 beamline of the European Synchrotron Radiation Facility (ESRF), Grenoble, using a wavelength of 0.5259 (2) Å. The 12 and 50 K data were collected using an Oxford Diffraction Helijet open-cycle helium Cryostream, while the data from 100 to 295 K were obtained using an Oxford Cryosystem open-cycle nitrogen Cryostream. A sample-to-detector distance of 5.82 cm gave a resolution at the edge of the CCD detector of 0.657 Å.

Two series of 600 frames were collected with a ϕ-scan rotation width of 0.3 ° and with an exposure time of 1 s at fixed ω and χ positions (ω = −155, −115°; χ = 54.7°; 4.5 h total collection time per temperature). For the two helium-temperature data sets, the sampling of reciprocal space had to be reduced due to the steric limitation imposed by the Helijet equipment: series of 600 and 400 frames were collected at ω = −125 and −110°, respectively, with the same χ position, exposure time and ϕ rotation width as for the higher-temperature data sets. In all experiments, the detector was positioned at θ = 0°. For further experimental details see Table 2 ▶.

Cell dimensions and the space group were determined with the Bruker *SMART* software from all reflections with *I*/σ(*I*) ≥ 50 in the first 400 images of each data collection. Reflections were integrated with the program *SAINT*, resulting in ∼2500 unique reflections for each temperature. Because of the short wavelength used in the experiment, no absorption correction was made. The data were also not corrected for oblique incidence effects because at the time of the measurement (2005) the necessary software was not generally available and the characteristics of the phosphorescent layer for the detector are not resurrectable. Estimates of such a correction are described in §[Sec sec4.4.1]4.4.1.

Initial structure models were obtained by direct methods and refined by full-matrix least-squares on *F* ^2^ using *SHELX97* (Sheldrick, 2008 ▶). All non-H atoms were refined anisotropically. H atoms were located in the difference Fourier map and refined freely with an isotropic displacement parameter.

### 3.3. Hirshfeld atom refinement (HAR) of X-ray data   

This section describes the basic idea behind the new automated iterative Hirshfeld atom refinement, gives some general technical details of the procedure and the specific details for the Gly–l-Ala refinements.

HAR requires a provisional set of atomic coordinates, either obtained from a spherical or an aspherical atom refinement. With these coordinates, a first electron density (ED) of a molecule representing at least the asymmetric unit is calculated from a quantum mechanical wavefunction. This ED is divided up into Hirshfeld atoms. The charges and moments of these atoms are calculated and used to simulate the electric field surrounding the molecule of interest. A new ED is then calculated in the presence of the simulated crystal field. We call this an ED cycle. ED cycles are repeated to convergence in the molecular energy. Together they are called an ED step. Subsequently, the Fourier transforms of the Hirshfeld atoms are used as non-spherical atomic scattering factors in a conventional crystallographic least-squares refinement of the coordinates and ADPs. This step, called a structural refinement step, usually consists of several least-squares cycles. The sequence of an ED step followed by a structural refinement step is called an ‘HAR iteration’. These iterations are repeated until both ED cycles and refinement cycles show no further changes.

*Quantum mechanical electron density calculations*. In this paper and all others to date, the EDs are calculated using the Hartree–Fock (HF) and BLYP density functional theory methods (DFT). DFT calculations employ the Becke numerical integration technique (Becke, 1988 ▶) modified by the efficient scheme suggested by Stratmann *et al.* (1996 ▶). The Mura radial integration grids (Mura & Knowles, 1996 ▶) are used with 30 points plus five for every principle quantum number above one. For the angular part, the Lebedev procedure is used (Lebedev & Laikov, 1999 ▶). It is capable of integrating up to an angular momentum of *L* = 29 (35) for H (non-H) atoms.

For both HF and BLYP, the wavefunction is obtained using the standard self-consistent field (SCF) method. Convergence is assumed when changes in the electronic energy (and its gradient) are less than 0.0005 (0.001) a.u. Considerable savings in computation are obtained by initiating the wavefunction calculations with the ED from the previous cycle, and similarly if refinements for larger basis sets are initiated from structural parameters obtained from HAR refinements using a basis set slightly smaller in size.

*Atomic partitioning*. The method of Hirshfeld (1977 ▶) is used to partition the molecular electron density into atomic contributions. The Hirshfeld or stockholder weight function for an atom is the spherically averaged atomic ED divided by the sum of spherical EDs of all atoms in the molecule chosen for the wavefunction calculation – not the ED in the whole crystal. There are two main reasons for not using the sum of electron densities of all the atoms in the crystal. First, the underlying wavefunction is essentially molecular. Second, moving beyond a molecular partitioning is computationally more demanding. Resources permitting, one could choose an ED model where the ‘molecule’ is a cluster of several mol­ecules.

The spherically averaged atomic EDs are not taken from tables but calculated as needed with the unrestricted method (different spatial orbitals for different spins) and the same wavefunction method (HF or BLYP) and basis set as used for the corresponding molecular calculation. This only takes a few seconds; in fact, less than reading in stored tables.

*Calculation and placement of charges simulating the crystal field*. The Hirshfeld charges and dipole moments of each atom are calculated numerically *via* the Becke (1988 ▶) method with the Hirshfeld atomic weight function, *i.e.* without using the Stratmann *et al.* (1996 ▶) extension. Charges are placed on atomic sites surrounding the central molecule for which the wavefunction is calculated. The distance between positive and negative charges used to simulate the atomic dipoles on each site is 0.001 a.u. Charges are placed on all complete molecules which have at least one atom within a specified distance of the central molecule. Complete molecules are used, to avoid charged species and therefore minimize errors in slowly converging potential sums.

*Structural refinement step*. Structural least-squares refinement uses the Fourier-transformed Hirshfeld atoms from the ED step as atomic scattering factors. The atomic positions and ADPs are optimized under the assumption that the Hirshfeld atoms move ‘rigidly’, *i.e.* they are only translated, but not rotated nor deformed. The least-squares matrix is pseudo-inverted, so that symmetry-redundant and numerically ill-defined parameter combinations can be eliminated, and the covariance matrix and errors properly calculated. The details of how this is done when symmetry is present, and taking into account the nonlinear relations between model parameters and derived quantities (such as bond lengths, angles and torsion angles), are described by Sands (1966 ▶). Least-squares cycles are repeated until the parameter shifts divided by the su’s of the parameters are less than 0.01.

Since EDs are obtained from wavefunctions calculated in the Cartesian coordinate system, HAR is performed in the same system. Consequently, errors associated with cell-parameter determination on derived properties such as bond lengths and angles do not arise. (Note that, in an aspherical atomic scattering formalism, there is no benefit in using fractional coordinates at all.)

*Application to Gly–l-Ala*. The ED calculations were carried out with three increasingly better Gaussian basis sets: cc-pVDZ, cc-pVTZ and cc-pVQZ (Dunning, 1989 ▶). All cc-pVDZ calculations were initiated with atomic positions from invariom refinements (Dittrich *et al.*, 2013 ▶). The initial displacement parameters on the H atoms were isotropic, but were refined anisotropically in the HARs. The crystal field was simulated by placing atomic charges and dipole moments on 55 complete molecules which had at least one atom within 8 Å of the central molecule. The eigenvalues of the least-squares matrix were always clearly non-zero, so there were never any undetermined coordinates or ADPs. There could be as many as 15 rigid-atom fit cycles in the first coordinate and ADP refinement step. Typically, only four of the HAR iterations were required for complete optimization, the penultimate cycle being converged in χ^2^ (Δχ^2^ ≤ 0.001). We did not observe any problems starting the wavefunction calculations from EDs obtained from a previous iteration.

*Software*. All calculations were performed using revision 4009 of the open-source *Tonto* program (http://sourceforge.net/projects/tonto-chem, later versions and bug fixes may be obtained from https://github.com/dylan-jayatilaka/tonto) which includes the option to perform ED steps and structural refinement steps, *i.e.* HAR iterations, to convergence automatically. The wavefunction calculations were performed in parallel using the MPICH2 library compiled with the gfortran compiler and executed on a Linux platform. Indicative wall clock time for the 12 K data set refinement at the cc-pVTZ level was 3 (6) h for the HF (BLYP) calculations on 16 processors. These timings varied for different temperatures and basis sets depending on the number of SCF cycles, and – to a much smaller extent – the number of refinement cycles needed in a particular case.

### 3.4. Statistical analysis   

Several statistics are used for comparing the X-ray and neutron measurements in §4 and in the supporting information. The formulae and names for these statistics are collected here, and discussed.

Suppose that we have a set of *N* values *V* = {*V*_*i*_}. Then the mean value and its population standard deviation are defined in the usual way by, respectively
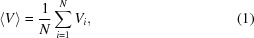


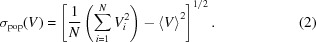
The population standard deviation σ_pop_ is also called the root mean-square deviation (RMSD). It gives an indication of the spread of the values around the mean. It is *not* an estimate of the error in the mean; that is given by
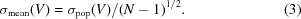
It is this quantity which should be used when judging whether trends in mean values are significant. This elementary fact is pointed out here only because on this subject in the literature there is a confusing but accepted notation for indicating errors in, and distribution widths of, a particular quantity: namely that an *individual* quantity written as 0.123 (4) indicates a standard uncertainty (su) of 0.004 associated with the value 0.123 (obtained by propagation of errors), whereas when the same refers to an *averaged* quantity, the bracketed term refers to a population standard deviation of 0.004 [see *e.g.* Iversen *et al.* (1996 ▶) and Morgenroth *et al.* (2008 ▶)].

In this paper we compare several pairs of data sets comprised of values derived from the X-ray and neutron measurements, respectively, denoted {X_*i*_} and {N_*i*_} below. These two sets are combined into measures of similarity/difference in several ways. The neutron value is always subtracted from the X-ray value or divides it. This is purely conventional and should not be taken to imply that the neutron experimental values are more accurate. The mean value and its population standard deviation for the combined set *V* are then reported, with the following nomenclature:

(i) The mean absolute difference (MAD), denoted 〈|Δ*P*|〉, is associated with the set *V* = |Δ*P*| = {|X_*i*_ − N_*i*_|}.

(ii) The mean difference (MD), denoted 〈Δ*P*〉, is associated with the set *V* = Δ*P* = {(X_*i*_ − N_*i*_)}. This quantity is also known as the signed difference. Unlike the MAD, the MD can be positive or negative, meaning that on average the parameters derived from the X-ray measurements are larger or smaller, respectively, than those derived from the neutron measurements.

(iii) The mean of the square of the weighted difference – weighted by the combined standard uncertainties from both measurements – is denoted 〈[Δ*P*/csu(*P*)]^2^〉. It is associated with the set *V* = {[(X_*i*_ − N_*i*_)/csu(X_*i*_,N_*i*_)]^2^}. The combined standard uncertainty (csu), which appears in this expression, is given by (Schwarzenbach *et al.*, 1995 ▶)
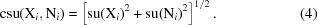
Combining these equations, the mean of the square of the weighted difference is
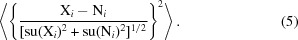
For reasons of convention, we report the square root of this property and refer to it as the csu-weighted root mean-square difference (wRMSD). It is meaningless to report a population standard deviation. The wRMSD values should be equal to 1 if the two sets of data are statistically in agreement, although in practice values of 1.5–2 are often found when comparing multiple determinations of the same crystal structure (Taylor & Kennard, 1983*a* ▶,*b* ▶; Martin & Orpen, 1996 ▶).

(iv) The mean ratio (MR), denoted 〈*r*〉, is associated with the set *V* = *r* = {(X_*i*_/N_*i*_)}. This statistic only makes sense when the parameter values to be compared may not be zero. Thus, it is not reported for coordinates, and is only reported for the diagonal ADPs (off-diagonal ADPs may be zero). The MR quantifies information similar to the MD.

The χ^2^ and *R*-factor statistics reported for the HARs are defined and reported in the usual way for crystallographic refinements.

## 4. Results   

The following sections primarily discuss comparisons between the HAR X-ray and the neutron results, in particular their dependence on basis sets, the quantum mechanical model and temperature. §4.1 deals with agreement factors; §§4.2–4.4 [Sec sec4.3] [Sec sec4.4] deal with the coordinates, bond distances and ADPs involving C, N and O atoms on one hand, and H atoms on the other. Some comments on outliers are collected in §4.5. Our aim in these comparisons is to validate the structural parameters obtained from HARs.

**Figure 1:**
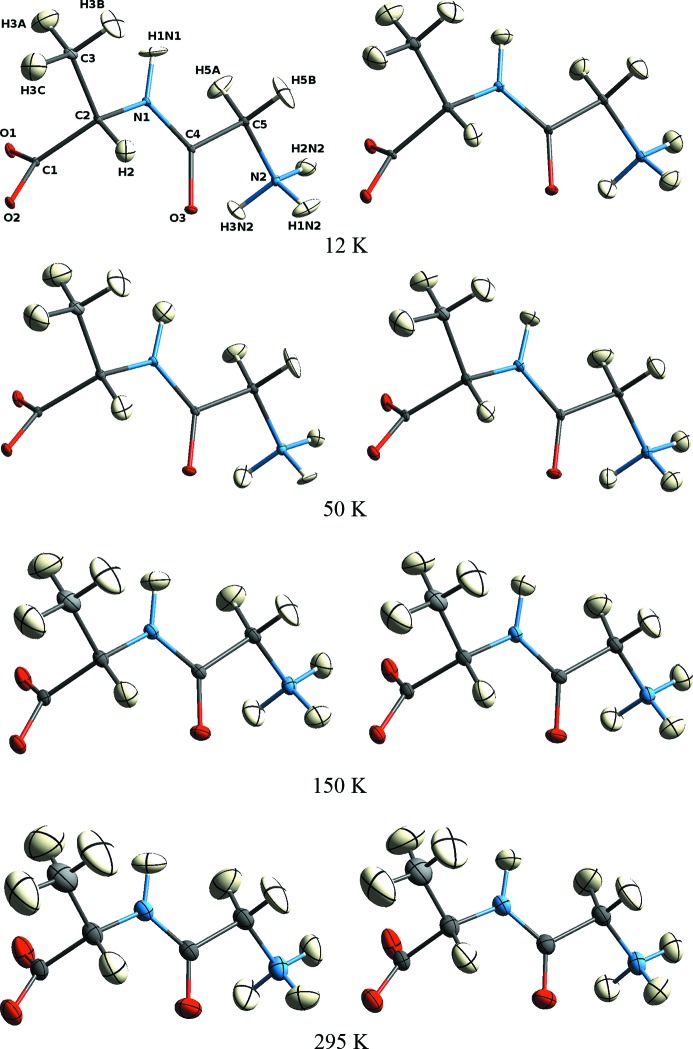
Projections of the Gly–l-Ala molecule as obtained from X-ray data after HAR at the BLYP/cc-pVTZ level (left column) and from neutron data (right column). ADPs are shown at the 50% probability level. The atom-numbering scheme at the top left is used throughout.

Fig. 1 ▶ shows the structures of Gly–l-Ala at all temperatures with anisotropic H atoms derived from X-ray and neutron data and establishes the labelling scheme used in the following sections. Further figures depict the differences between X-ray and neutron derived coordinates, bond lengths and ADPs in terms of histogram and frequency plots relative to the parameter su’s and csu’s. Tables summarize the X-ray–neutron comparisons in terms of the statistical properties discussed in §3.4. Because of the large volume of data, only the most interesting and representative of these figures and tables are shown in this article. The complete set of figures and tables is given in the supporting information; it encompasses the differences between each X-ray and neutron derived parameter at each temperature, for each basis set, and with both methods HF and BLYP. (Throughout the text, we refer to the supplementary tables and figures using the label ‘S’, *e.g.* Table S1.) Individual atomic coordinates, bond distances, ADPs and other information concerning the X-ray HAR and neutron refinements from which the figures and tables are derived are given in the supporting information in the form of CIFs. Additionally, for the HAR results at the BLYP/cc-pVTZ level of theory, CIFs at all six temperatures (12, 50, 100, 150, 220 and 295 K) have been deposited with the Cambridge Structural Database with deposition codes CCDC 995876–995881, and can be downloaded free of charge from www.ccdc.cam.ac.uk/data request/cif.

### 4.1. Figures of merit   

The χ^2^ statistics have been obtained with least-squares weights equal to 1/su^2^(|*F*_obs_|). The values collected in Tables 3 ▶ and S1 cover the range ∼1.0–1.6. The data show two main trends. First, the χ^2^ for all temperatures and all basis sets are lower by ∼0.09–0.15 for refinements with the BLYP than for those with the HF method. Second, the differences between the basis sets cc-pVDZ and cc-pVTZ are small, ∼0.02–0.06; those between cc-pVTZ and cc-pVQZ are negligible. This shows that the experiment can distinguish between different quality basis sets in the expected order and that HAR based on the cc-pVTZ basis set is sufficiently converged. On the basis of the χ^2^ statistics, the conclusion follows that BLYP/cc-pVTZ is the preferred level of theory. The χ^2^ statistics as a function of temperature for BLYP/cc-pVTZ vary from 0.96 to 1.19, but show no obvious trend. The trends in the statistics *R*(|*F*|) and *wR*(|*F*|) are similar but less pronounced (Tables S2 and S3).

### 4.2. Comparison of positional parameters   

#### 4.2.1. Non-H atoms   

The mean absolute differences (MADs) of non-H-atom positions between the X-ray and neutron experiments are ∼0.0002–0.0003 in fractional coordinates or ∼0.002–0.003 Å (Table S4). Since the precision of the non-H coordinates from the X-ray experiment is significantly better than that from the neutron experiment (Figs. S1–S6), the neutron su’s are the limiting factor for any comparison. The MD values (Table S5), although close to zero (2–9 × 10^−5^), are all positive, indicating that on average the X-ray coordinates are slightly larger than the corresponding neutron ones. However, the σ_mean_ values are larger than the MD values themselves which means that this trend is insignificant. The MAD and MD values for HF and BLYP calculations with any of the three basis sets are very similar for each temperature (Tables S4 and S5). The corresponding histograms and frequency plots of individual csu-weighted differences (Figs. S1–S6) are also very similar with respect to changes in method and basis set; possibly the histograms are slightly more symmetrical for the data from the BLYP than from the HF refinements. With respect to temperature, there appears to be a tendency that the agreement between X-ray and neutron experiments is best at 12 K and worsens towards higher temperatures, as may be seen in the MAD and MD values (Tables S4 and S5), as well as in Figs. S1–S6. However, the wRMSD values (Table S6) are all close to 1 and stay nearly the same with increasing temperature because the magnitudes of the su’s of the coordinates also increase with temperature.

#### 4.2.2. H atoms   

**Figure 2:**
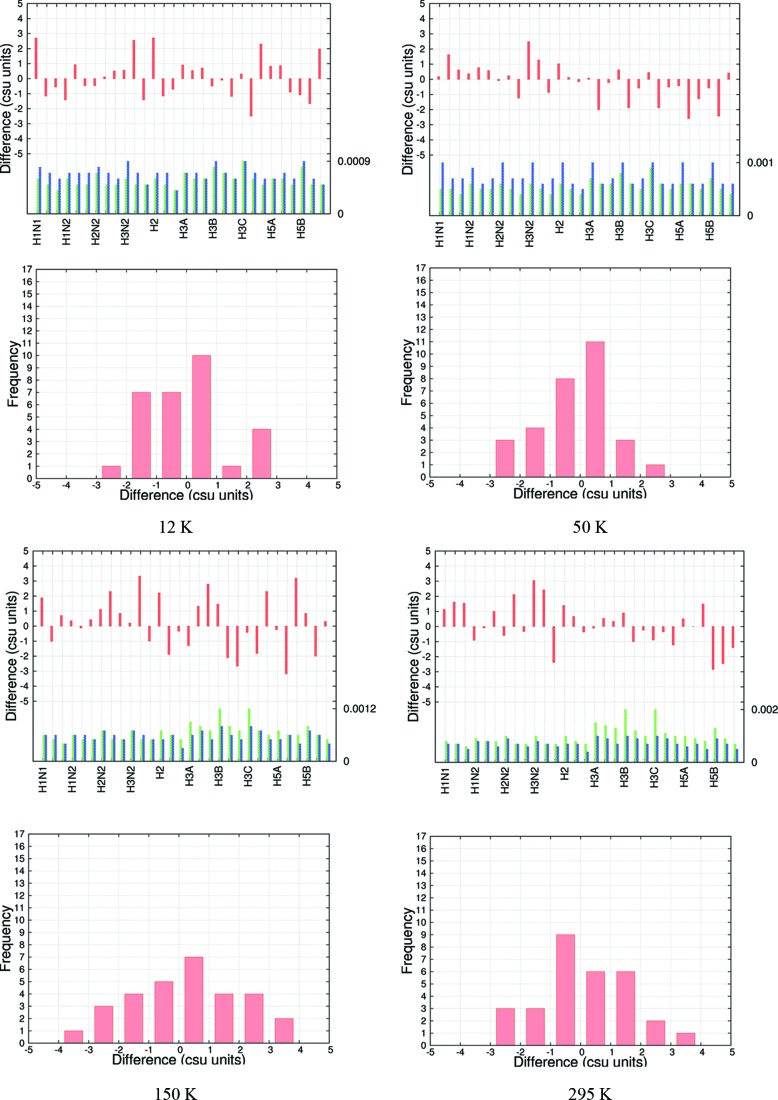
Csu-weighted differences between X-ray and neutron measurements for H-atom fractional coordinates at four temperatures for the model ED at BLYP/cc-pVTZ. Rows 1 and 3: histograms with three entries (*x*, *y*, *z*) per atom. Coordinate su’s are shown for neutron (green) and X-ray (blue) measurements; the maximum su value is shown on the right axis. Rows 2 and 4: frequency plots.

As expounded in §1, obtaining the positions of and bonds to H atoms from X-ray data is of special interest. Two important aspects of this problem are the precision of the refined coordinates and their accuracy as judged from a comparison with neutron diffraction data. Remarkably, Figs. 2 ▶ and S7–S12 show that the precision of the H-atom fractional coordinates from the X-ray and neutron experiments is nearly the same. At 12 and 50 K, the su’s are ∼0.0007 or ∼0.007 Å; at the higher temperatures they are 50% larger. For comparison, we mention again the precision of 0.007 Å quoted by Destro & Merati (1995 ▶) in a multipole analysis of X-ray data with a high resolution (*d*_max_ ≃ 0.44 Å). We achieve a comparable result with relatively low-resolution data (*d*_max_ ≃ 0.65 Å).

Table 4 ▶ shows that the mean absolute differences between the X-ray and neutron results are ∼0.001 or 0.01 Å, respectively, *i.e.* about ten times larger than those for the non-H atoms. The wRMSD values which take into account the errors in both X-ray and neutron measurements are between 1.0–2.0 (Table S9). Thus, the MAD and wRMSD values show that the non-H-atom coordinates are determined more accurately than the H-atom coordinates, but in practice (see §3.4) the H-atom coordinates from the HAR procedure are also in statistical agreement with those from neutron diffraction.

In all the measures discussed (MADs, Table 4 ▶; MDs, Table S8; wRMSDs, Table S9), the values for the BLYP calculations are systematically better than those for the HF ones (*e.g.* 1.07–1.75 *versus* 1.41–1.99 in wRMSD). The csu-weighted differences are roughly normally distributed, with a slight decrease in width from HF to BLYP calculations (Figs. S7–S12). These observations imply that electron correlation effects are important in order to secure more accurate H-atom positions in the refinements.

It is interesting and perhaps unexpected that the H-atom coordinates obtained in the HARs with the smallest basis set (cc-pVDZ) show the same or even better agreement with the coordinates from the neutron measurememts than those with larger basis sets. This is reflected in all statistical properties (Tables 4 ▶ and S8 and S9) and in the plots (Figs. S7–S12). The X-ray H-atom coordinates seem to be insensitive to subtle differences in the theoretical electron densities due to different basis sets. This may explain why the multipole model is generally so successful with a rather small number of radial functions.

The distributions in Fig. 2 ▶ show the effect of temperature on the results. There is a small increase in distribution width with increasing temperature. This trend can also be observed in Table 4 ▶, where the MADs overall become larger with increasing temperature.

### 4.3. Comparison of bond lengths   

#### 4.3.1. Bonds between non-H atoms   

The precision of determining the non-H bond lengths is significantly higher from the X-ray experiments than from the neutron experiments (Figs. S13–S18) in correspondence with the same observation for the coordinates. Therefore, the su’s of the neutron diffraction experiments are the limiting factor of the comparisons. The accuracy of the non-H bond lengths as determined from the comparison of X-ray and neutron experiments is about 0.001–0.003 Å if measured in terms of mean absolute differences (MADs, Table S10).

The signed mean differences (MDs, Table S11) show that, in the low-temperature experiments (12, 50 K), the distances derived from the X-ray experiments are slightly longer by ∼0.0002–0.0009 Å than those derived from the neutron experiments. In contrast, in the higher-temperature experiments (150, 295 K) the bonds from the neutron experiments are slightly longer by up to 0.0005 Å. The origin of this seemingly systematic trend is unclear, but we recall that the 12 and 50 K measurements were conducted using an open-flow helium device, whereas the setup was changed for the 150 and 295 K measurements to open-flow nitrogen cooling.

There is no dependency of the various quantities with respect to the method used (HF *versus* BLYP). The smallest basis set cc-pVDZ is sufficient, as the normality of the distributions (Figs. S12–S18) and the MDs (Table S11) do not improve with higher basis sets. The agreement between X-ray and neutron experiments is clearly best at 12 K and worsens noticeably towards higher temperature, as visible in terms of MAD trends (Table S10) and the csu-weighted differences (Figs. S13–S18).

#### 4.3.2. Bonds involving an H atom (*D*—H)   

The N—H and C—H distances from both X-ray (BLYP/cc-pVTZ) and neutron experiments are summarized as a function of temperature in Table 5 ▶. The N—H amide distances from both experiments are shorter by ∼0.02–0.03 Å than the N—H ammonium distances at all temperatures. This is in agreement with the average neutron reference values (Allen & Bruno, 2010 ▶). The distinction of the C—H distances is a bit less clear, but in most cases *d*(C—H) > *d*(C—H_2_) > *d*(C—H_3_). Although the comparisons for individual experiments at a single temperature may not look significant, the overall trend found from eight independent experiments certainly is (Table 5 ▶). It shows that trends in *D*—H distances obtained from HAR are accurate enough to distinguish between different functional groups.

The mean absolute differences between *D*—H distances from X-ray and neutron experiments are 0.007–0.013 Å (Tables 6 ▶ and S14). From Table 5 ▶ it can be deduced that the average differences are somewhat larger for the N—H bonds (0.009 Å) than for the C—H bonds (0.004 Å). The reason for this is likely that all of the N—H bonds are involved in relatively short intermolecular contacts (Table 7 ▶). HAR does not have basis functions at the acceptor atoms and might therefore introduce a small bias when describing *D*—H bonds involved in hydrogen-bonding interactions (see further discussion in §5). For C—H bonds this problem is much less severe because the acceptor atoms of intermolecular interactions are usually at larger distances (Table 7 ▶). An approach analogous to the supramolecular synthon-based fragments database of Hathwar *et al.* (2011 ▶) might be needed here.

**Figure 3:**
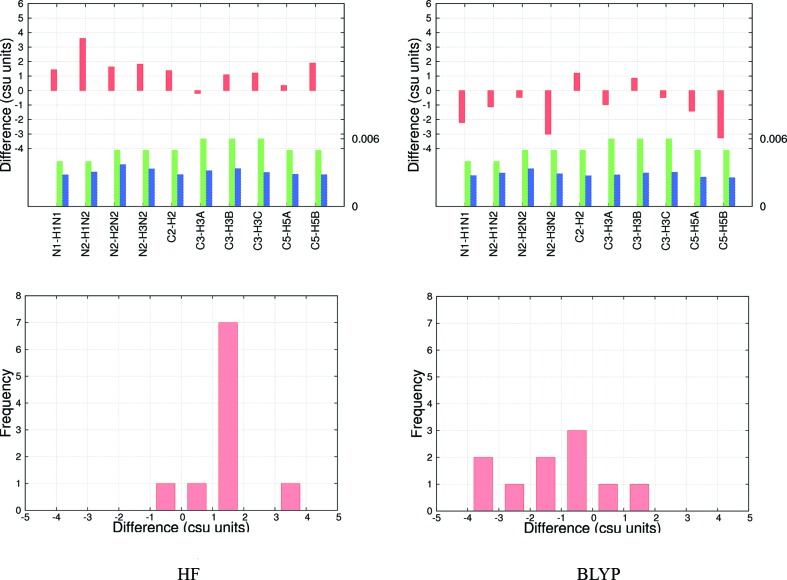
Csu-weighted differences (Å) between X-ray and neutron measurements for *D*—H bond distances, at 12 K for the Hartree–Fock (left) and BLYP model EDs (right) with the cc-pVTZ basis set. Top row: histograms. *D*—H su’s (Å) are shown for neutron (green) and X-ray (blue) measurements; the maximum su value is shown on the right axis. Bottom row: frequency plots.

The su’s of the X-ray *D*—H distances from HAR at 12 and 50 K are no larger than 0.006 Å (Figs. 3 ▶ and S19–S24), about the same as the neutron su’s or up to 50% higher. The precision becomes much worse relative to neutron measurements at higher temperatures. Assuming that these measurement errors are realistic, agreement between the X-ray and neutron *D*—H bond lengths measured in terms of the wRMSDs (1.2–3.0) is rather poor (Tables 6 ▶ and S16). This is in contrast with the MADs discussed above, which are excellent and probably overall the best reported so far in the literature.

There is a systematic difference between *D*—H distances based on HF and BLYP refinements. At the HF level the bond lengths are systematically too long, while at the BLYP level the bond lengths are systematically too short. This can be seen clearly in Fig. 3 ▶, where the effect of introducing electron correlation is visible as positive differences in the HF histogram but negative differences in the BLYP histrogram. This corresponds to a right-skewness (positive csu units) of the distribution in the HF frequency plot (Fig. 3 ▶), changing towards a left-skewness (negative csu units) of the distribution in the BLYP frequency plot. The same fact appears as a change in the sign of the MD values between HF and BLYP refinements (Tables 6 ▶ and S15) regardless of basis set and temperature. From these results we might hypothesize that a ‘hybrid’ density functional theory method which combines both exact HF exchange and DFT correlation, *e.g.* the popular B3LYP functional, may produce better bond lengths. Such hybrid functionals are known to produce better agreement for properties strongly correlated with the electron density, such as bond lengths and vibrational frequencies (Wong, 1996 ▶; Riley *et al.*, 2007 ▶), optical properties (Stephens *et al.*, 1994 ▶) and the density itself (Wang *et al.*, 1996 ▶).

With regard to trends in the basis set, it is clear that the cc-pVTZ basis set is sufficient for accurate *D*—H bond lengths (Figs. S19–24, Tables S14 and S15).

Table 7 ▶ lists the closest intermolecular distances from neutron diffraction and BLYP/cc-pVTZ HAR at 12 K. The carboxylic acid O atoms are each involved in two hydrogen bonds with the H atoms on the N atoms, while the carbonyl O atom forms two contacts with one methyl and one methylene H atom. In four out of six cases the H⋯*A* distances from HAR overlap with the ranges of the values from the neutron diffraction experiment, while for the D—H⋯*A* angles only N1—H1N1⋯O2 is out of range. This reflects the excellent agreement of *D*—H bond distances between neutron and HAR results discussed above.

### 4.4. Comparison of ADPs   

#### 4.4.1. Non-H atoms   

**Figure 4:**
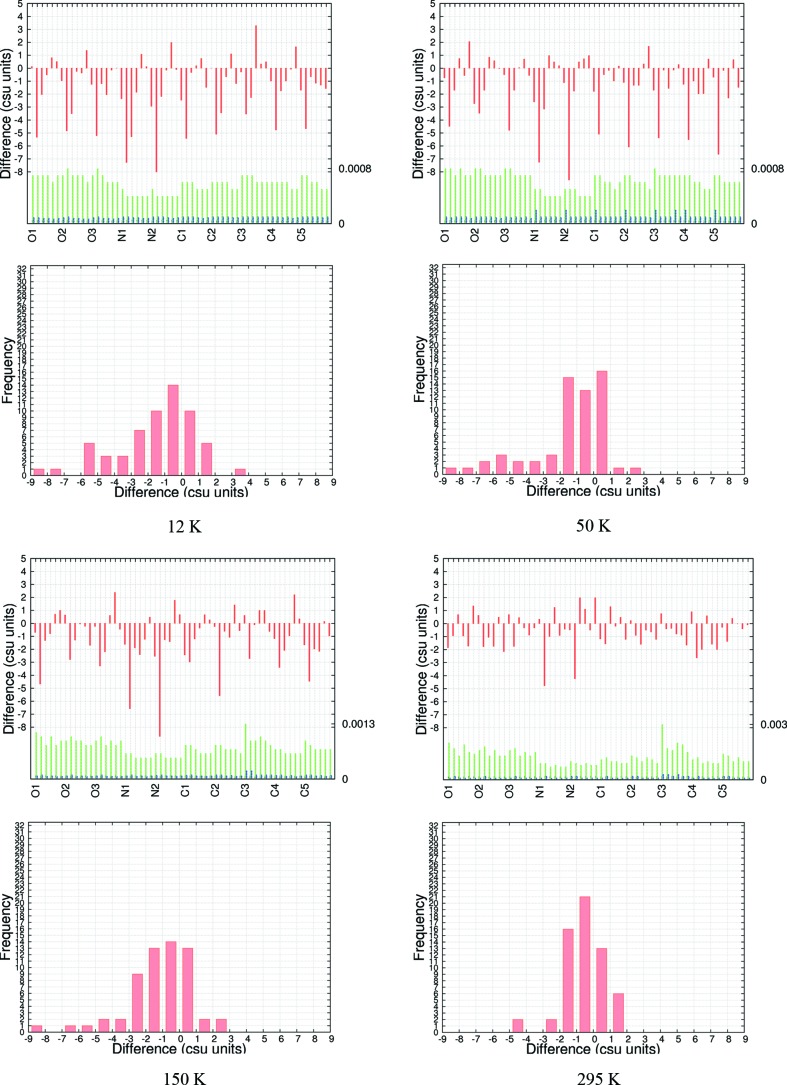
Csu-weighted differences between X-ray and neutron measurements for non-H-atom ADPs, at four temperatures for the best model EDs at BLYP/cc-pVTZ. Rows 1 and 3: histograms with six entries (*U*_11_, *U*_22_, *U*_33_, *U*_12_, *U*_13_, *U*_23_) per atom. ADP su’s (Å^2^) are shown for neutron (green) and X-ray (blue) measurements; the maximum su value is shown on the right axis. Rows 2 and 4: frequency plots.

The signed 〈Δ*U*_*ij*_〉 values (MDs) in Tables 8 ▶ and S19 show significant negative values throughout, and the mean ratios 〈*U*_*ii*_(X-ray)/*U*_*ii*_(neutron)〉 (MRs) in Tables 8 ▶ and S21 are below 1 by ∼30–25% (12 K), 25–22% (50 K), 13–10% (150 K) and 5–3% (295 K). This means that the ADPs from the X-ray experiment are too small. The same effect is visible as large negative csu-weighted differences in the histograms of Figs. 4 ▶ and S25–S30, especially for *U*_22_, as well as in a left-skewness of the distributions in the frequency plots in the same figures. Visually, this skewness is reduced significantly towards 295 K with correspondingly better csu-weighted differences in the histograms.

We ascribe these effects to the lack of an oblique-incidence correction in the X-ray experiments which must lead to an underestimation of ADPs. This hypothesis is also consistent with the fact that the effect is less pronounced at higher temperatures, where high-order reflections at the edge of the detector are less intense and have a lower weight in the least-squares refinement compared with lower temperatures. Since the correction curve for the detector is not available to us, we have attempted an estimate of the effect.

We have assumed that the errors in the intensities are isotropic and can be absorbed into an isotropic contribution to the *U*_eq_. This contribution would be related to the ratio *I*_corr_/*I*_uncorr_ of the corrected and uncorrected intensities at the maximum scattering angle according to
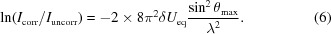
For ratios *I*_corr_/*I*_uncorr_ of 1.1, 1.2, 1.3, 1.4 and 1.5, the quantity δ*U*_eq_ is 0.0010, 0.0019, 0.0028, 0.0036 and 0.0043 Å^2^, respectively. Ratios of 1.3–1.5 are not unreasonable, as shown by Johnas *et al.* (2006 ▶) and Poulsen *et al.* (2007 ▶). The corresponding values of δ*U*_eq_, 0.0028–0.0043 Å^2^, may be compared with the average differences for the *U*_22_ values of ∼0.004 Å^2^ at 12 K (BLYP/cc-pVTZ, Fig. 4 ▶). As discussed above, the differences become smaller with increasing temperature. Although such arguments based on errors in the X-ray data reduction process rationalize the large X-ray–neutron differences, we cannot exclude unknown systematic errors in the neutron ADPs. We have therefore refrained from applying scale factors to the HAR ADPs to make them larger artificially.

The shortcomings discussed above do not show in the differences *U*(*X*_*i*_) − *U*(*X*_*j*_) (DMSDA, differences of the mean-square displacement amplitudes) along the *X*_*i*_—*X*_*j*_ bond, the quantities underlying the so-called Hirshfeld rigid-bond test. For the HARs at BLYP/cc-pVTZ level, 33 of the DMSDA values are in the range 0.0000–0.0005 Å^2^ (Table S26), well within the limit of 0.001 Å^2^ suggested by Hirshfeld (1976 ▶). The remaining three values are 0.0006 (150 K), 0.0007 and 0.0013 Å^2^ (295 K). The csu’s are 0.0001–0.0002 Å^2^. The corresponding DMSDA values derived from the neutron diffraction experiments cover a much larger range (0.0001–0.0040 Å^2^), but their csu’s are also larger by about an order of magnitude (0.001–0.002 Å^2^). The finding for the X-ray values confirms the earlier conclusion that difference displacement parameters are generally more physically meaningful than the displacement parameters themselves. The phenomenon is due to the fact that the systematic errors in the ADPs vary little between atoms and cancel largely on taking differences (Ammeter *et al.*, 1979 ▶; Chandrasekhar & Bürgi, 1984 ▶).

In addition, a few results from Tables 8 ▶ and S18–S21 are also worth mentioning. As for the other quantities involving only non-H atoms, the precision of the ADPs from the X-ray experiments is significantly higher than that from the neutron experiments (see Figs. 4 ▶ and S25–S30). The mean absolute differences (MADs, 〈|Δ*U*_*ij*_|〉), a measure of the accuracy of determination of the non-H ADPs, are ∼0.0011–0.0013 Å^2^ (Table 8 ▶ and S18). These values are about an order of magnitude larger than the smallest values in Iversen *et al.* (1996 ▶) and Morgenroth *et al.* (2008 ▶), but are within the spread of 〈|Δ*U*_*ij*_|〉 values for organic compounds listed by Morgenroth *et al.* (2008 ▶; Table 2). In fact, if judged from MADs, MDs and wRMSDs together (Table 8 ▶ and S18–S20), our results are in the top 50% of the studies listed by Morgenroth *et al.* (2008 ▶).^2^ This is surprising given the problems discussed with the missing oblique-incidence correction. Moreover, several of the values listed by Morgenroth *et al.* (2008 ▶) are based on studies in which H-atom positions and ADPs were fixed to values derived from neutron experiments. By contrast, in this study all parameters are derived from the X-ray data only and without constraints to the neutron experiment.

The dependence of the average and individual differences on the method used (HF *versus* BLYP) is minimal (Tables 8 ▶ and S18–S21 as well as Figs. 4 ▶ and S25–S30). Differences derived from HF calculations are generally slightly smaller than those from BLYP. Within BLYP, there is an improvement in the agreements with increasing size of basis set. The cc-pVTZ basis set clearly performs better than cc-pVDZ and is sufficient as the differences from the cc-pVQZ basis set become insignificant.

With increasing temperature, agreement between neutron and X-ray ADPs becomes worse as far as MADs are concerned (Tables 8 ▶ and S18). This trend is reversed in the wRMSDs (Tables 8 ▶ and S20) by the increase in the su’s with increasing temperature, and, as discussed above, the csu-weighted differences in Fig. 4 ▶ become smaller with increasing temperature and the distributions appear more normal. This observation emphasizes the fact that distribution widths and averaged differences are complementary pieces of information.

#### 4.4.2. H atoms   

The ADPs obtained from the X-ray data with BLYP/cc-pVTZ EDs are shown in Fig. 1 ▶, together with the corresponding ADPs from the neutron diffraction study. The ellipsoids for the H atoms allow some important, albeit qualitative, conclusions. They increase in size with temperature, as one would expect, and their orientations are closely similar at the different temperatures, although they are from eight independent experiments. The anisotropies of the methyl and ammonium H atoms indicate torsional motion. The remarkable consistency with respect to the anisotropies and general orientations of the ellipsoids from neutron and X-ray diffraction indicates that, overall, the H-atom ADPs from HAR represent physically meaningful information.

**Figure 5:**
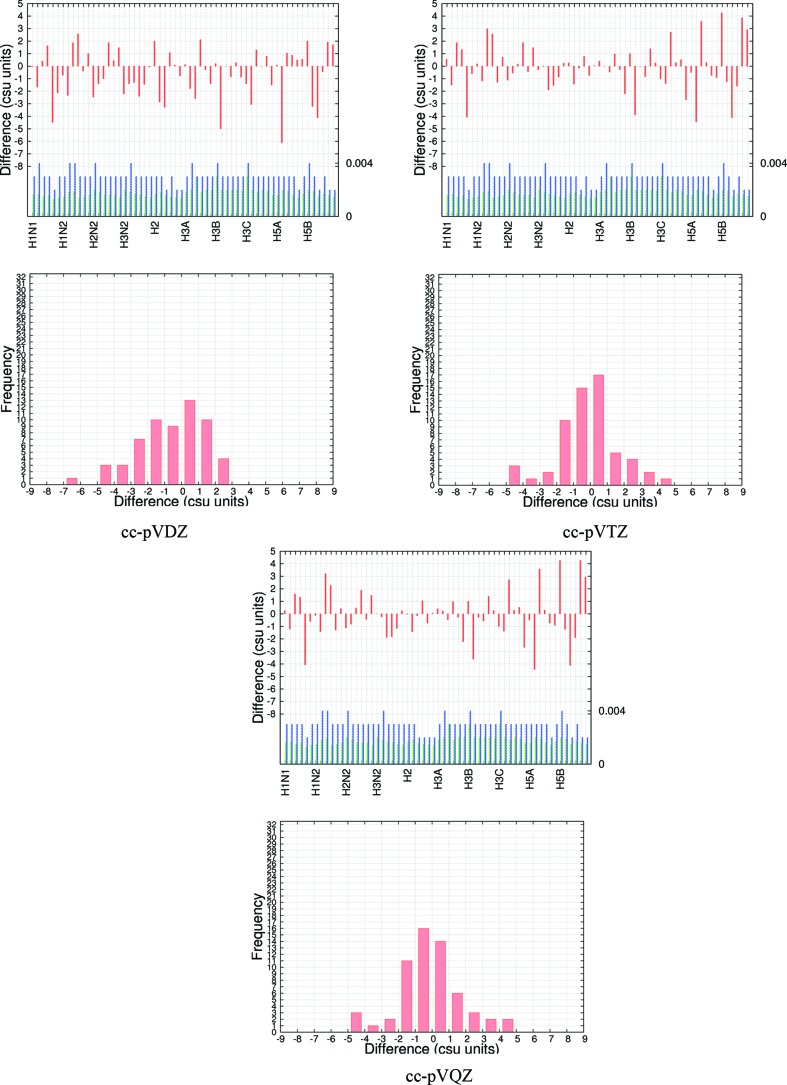
Csu-weighted differences between X-ray and neutron measurements for hydrogen ADPs at 12 K with the BLYP method at all three different basis sets. Rows 1 and 3: histograms with six entries (*U*_11_, *U*_22_, *U*_33_, *U*_12_, *U*_13_, *U*_23_) per atom. ADP su’s (Å^2^) are shown for neutron (green) and X-ray (blue) measurements; the maximum su value is shown on the right axis. Rows 2 and 4: frequency plots.

The su’s of the ADPs at the lower temperatures (12 and 50 K) as derived from the X-ray data are smaller than those from the neutron measurements (0.002 Å^2^ *versus* 0.004–0.005 Å^2^, Figs. 5 ▶ and S31–S36). At the higher temperatures the precision is about the same, except for three very large su’s at 295 K (Figs. S31–S36).

The HAR DMSDA values *U*(*X*_*i*_) − *U*(H_*j*_) at the BLYP/cc-pVTZ level of theory are all negative except for one insignificantly positive value [0.0004 (33) Å^2^, 50 K, C5—H5A, Table S26]. The average DMSDA value over all four N—H bonds at all four temperatures is −0.0068 Å^2^; the corresponding average DMSDA value for all six C—H bonds is −0.0053 Å^2^. The averages derived from the neutron data are −0.0063 Å^2^ (N—H) and −0.0059 Å^2^ (C—H). These relatively large values reflect a mass effect related to the *X*—H stretching vibrations: the displacements of the lighter H atoms are significantly larger than those of the heavier *X* atoms. All values are in the expected ranges.

The mean absolute differences between the X-ray and neutron measurements are ∼0.004–0.006 Å^2^, the values at 295 K being larger (Tables 9 ▶ and S22). These MAD values are of the order of about a quarter to a tenth of the individual *U_ii_* values, which is a remarkable result for H-atom ADPs. The wRMSD values range between 1.5 and 2.0 (Tables 9 ▶ and S24), similar to the values observed for the H-atom coordinates.

The distributions of the csu-weighted differences are sufficiently normal with only some slight indications of skewness (Figs. 5 ▶ and S31–S36), in contrast with the same plots for the non-H ADPs (Fig. 4 ▶). In line with this finding, the systematic underestimate of X-ray ADPs that was striking for the non-H ADPs in MD and MR statistics (Table 8 ▶) and which was ascribed to a missing oblique-incidence correction is also *not* visible here, neither in the mean differences for the H-atom ADPs (Tables 9 ▶ and S24S23) nor in the mean ratios for the H-atom ADPs (Tables 9 ▶ and S25). In all probability, the information on the H-atom ADPs derives primarily from the low-angle reflections, which are less affected by oblique-incidence errors.

The MDs (Tables 9 ▶ and S23) are smaller at the HF than at the BLYP level (for all temperatures except 295 K). The same effect is visible in the MRs (Tables 9 ▶ and S25), where the values are closer to 1 for HF than for BLYP calculations (except for 295 K). On the other hand, for both MD and MR the σ_pop_ values are consistently larger for the HF than the BLYP results. Judging from the MADs and wRMSDs (Tables 9 ▶, S22 and S24), the BLYP calculations produce clearly better H-atom ADPs than the HF method. In summary, although the HF results have a larger spread around the neutron measurements, they are more accurate as judged from MD and MR; BLYP is more accurate as judged from MAD and wRMSD. Therefore, further experiments will be required to decide the best functional to use. In fact, such ADP measurements could be used to test different functionals for accuracy (*cf.* similar comments for *D*—H bond lengths in §4.3.2).

There is a significant improvement in the HAR ADPs with the basis set change from cc-pVDZ to cc-pVTZ. This is evident from the decrease in the mean differences in terms of MADs, MDs and wRMSDs (Tables S22, S23 and S24). The MRs are significantly closer to 1 if going from cc-pVDZ to cc-pVTZ (Table S25). In all of these quantities a further step from cc-pVTZ to cc-pVQZ brings no further improvement. This is depicted strikingly in Fig. 5 ▶, where from BLYP/cc-pVDZ to BLYP/cc-pVTZ the distribution becomes more normal and narrower with less severe csu-weighted differences in the histogram plot, whereas from BLYP/cc-pVTZ to BLYP/cc-pVQZ both histogram and frequency plots look virtually identical. This shows that the basis set cc-pVTZ is both necessary and sufficient. An improvement in the ADPs from cc-pVDZ to cc-pVTZ means an increase in X-ray ADP size seen in both MD and MR (Tables S23 and S25) because the reference values from the neutron experiment remain the same. The increase in ADPs may be rationalized by the fact that, when larger basis sets are used, there are generally a higher number of Gaussian functions employed to model the hydrogen 1*s* electrons, leading to a ‘sharper’ and ‘larger’ nuclear cusp. To compensate for this, it seems plausible that larger ADPs are required.

### 4.5. Outliers   

The data collected in the various tables and figures show some outliers which have not been discussed above.

(i) All indicators measuring X-ray–neutron differences of the non-H coordinates and bond lengths in the 50 K results are larger than those at 12 and 150 K which, together with the values for 295 K, increase smoothly (Tables S4–S6 and S10–S13).

(ii) The wRMSDs of the H-atom coordinates at 50 K are somewhat anomalous (Table S9): whereas the worst agreement is found at the HF/cc-pVTZ level (1.82), the BLYP/cc-pVTZ model shows the best agreement (1.19). The worst agreement in the wRMSDs of the *D*—H distances (2.99) is again found at 50 K, namely for the HF/cc-pVTZ model, whereas the best agreement (1.25) is for HF/cc-pVTZ at 295 K (Table 6 ▶).

(iii) At 50 K, the su’s for the H atom *x* coordinates from the HAR determinations are about half those for the corresponding H-atom *x* coordinates from the neutron measurements. Additionally, there seems to be a small systematic error in the H-atom *y* coordinates from the neutron measurements (see Figs. 2 ▶ and S7–S12).

(iv) It is conspicuous from the histogram plots in Figs. S13–S18 that the csu-weighted difference of the C1—C2 distance is consistently the largest at 50 K but it is not an outlier at the other temperatures. Inspection of the CIF files in the supporting information shows that the C1—C2 distance is overestimated in the 50 K neutron experiment [1.549 (2) Å], being otherwise in the range 1.540–1.542 Å for all other temperatures and experimental procedures (both X-ray and neutron, and different levels of theory for X-ray analysis).

These observations clearly show that there is an inconsistency or experimental error in the 50 K data, which is large enough to be detectable in some quantities and plots. From the two last points, it seems plausible that the error lies in the neutron experiment. Overall there are many different possible explanations for the error, so we were not able to trace its source.

## 5. Discussion   

HAR is a natural extension of previous schemes of structure refinement. It combines theoretical and experimental data in the same way the independent atom model (IAM) does. It may be worthwhile remembering here that the libraries of spherical atomic scattering factors widely used since the middle of the last century in the IAM have been derived from several different atomic models using sophisticated theoretical calculations, *e.g.* the Thomas–Fermi–Dirac, the Hartree–Fock–Slater, the Dirac–Slater or the Cowan–Griffin methods, the last for relativistic atomic theory. For a comparison of models see Cromer (1965 ▶). In that sense, HAR does not use more extensive or more sophisticated theory than the IAM, but it calculates tailor-made aspherical scattering factors on the fly (instead of using tables of spherical scattering factors) in order to make the most of the experimental data.

Developments beyond the IAM have become possible for two reasons. Firstly, the accuracy of the experiments has increased to the point where models of non-spherical EDs can be determined by multipole refinements. Secondly, advances in quantum mechanical theory and ever increasing computing power have made *ab initio* calculations of EDs tractable. These developments led to libraries of experimental and theoretical generalized X-ray scattering factors of aspherical pseudo-atoms, an earlier advancement of the IAM. HAR also takes advantage of these developments but uses customized molecular EDs and corresponding Hirshfeld atom scattering factors and is thus an obvious next step in structural modelling of X-ray diffraction data,^3^ as shown by the sequence
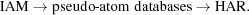
Free multipole modelling (as opposed to using multipoles as entries in pseudo-atom databases) is conspicuously absent from the sequence above. This is because the multipole model does not take any ED information from theory but refines it directly from the experimental data, unlike any of the methods above. The multipole model does, however, incorporate a significant and somewhat hidden contribution from theory in terms of the choice of the radial functions used. They are derived from atomic quantum mechanical models which have an exponential radial decay. Nevertheless, it is not a purely structural refinement method as the other methods are.

Following the basic philosophy behind multipole modelling, the final step of sophistication in the sequence shown above would be an iterative X-ray wavefunction refinement in which HAR is followed by an adjustment of the electronic wavefunction against the experimental data. This would take into account shortcomings of the theoretical model used to calculate EDs in HAR. In the present form, the term X-ray wavefunction refinement (XWR) refers to the subsequent execution of an iterative HAR, as introduced in this study, and an X-ray constrained wavefunction fitting procedure, introduced earlier by Jayatilaka (1998 ▶) and Jayatilaka & Grimwood (2001 ▶). The idea of XWR was first introduced by Grabowsky *et al.* (2012 ▶), and put into context with other methods by Grabowsky *et al.* (2013 ▶) and Schmøkel *et al.* (2013 ▶). The present XWR protocol was used by Chęcińska *et al.* (2013 ▶) and Zakrzewska *et al.* (2013 ▶), but could also be understood as the first cycle of a future iterative XWR.

There are advantages and shortcomings of HAR. Some significant advantages are as follows:

(i) *Tailor-made electron densities*. Database approaches use tabulated aspherical multipole-represented atomic densities according to different ‘atom types’ defined by the chemical environment. With these approaches, there are always lingering doubts about transferability, *i.e.* whether the database contains an entry which is sufficiently similar and in the adequate orientation for a given problem (Koritsanszky *et al.*, 2002 ▶; Grabowsky *et al.*, 2009 ▶). The iterative HAR scheme implemented in this study does not suffer from such problems, as EDs are always tailor-made for the molecule under investigation and are continually adjusted to small changes in the atomic coordinates.

(ii) *Easy applicability*. Although much research has been done on deriving and labelling database pseudo-atoms, one still requires a rather complex system of local coordinates to be defined according to atomic site symmetries. In contrast, HAR is as easy to apply as a normal IAM refinement using *e.g. SHELX* (Sheldrick, 2008 ▶), and therefore is accessible for the non-expert.

(iii) *Correlation between parameters is reduced*. Given an optimal approximation to the molecular ED, the correlation between ED and ADPs is minimized since the electron density is not refined directly. This has the potential to lead to physically more meaningful ADPs, even for H atoms.

(iv) *Phase uncertainty is reduced*. Since the EDs are not refined but obtained from a very accurate calculation, it might be expected that the phase uncertainty in the calculated structure factors in studies of non-centrosymmetric structures is also minimized (see *e.g.* Spackman & Byrom, 1997 ▶).

Limitations of HAR include the following, some of which are more technical in nature:

(i) *Computational cost*. One of the present limitations concerns the size of molecular asymmetric units that can be handled conveniently with the present implementation of HAR. Using 16 processors, computing time for a converged HAR of Gly–l-Ala with its 20 atoms is of the order of hours. However, such calculations scale linearly with the number of atoms *N* in the system at best, and with *N* ^2.5^ at worst. Increasing the number and efficiency of processors and optimization of the HAR code will make larger systems tractable, but applications in protein crystallography do not seem possible with the current algorithm.

(ii) *Application to network structures*. The present implementation of HAR uses molecular wavefunctions. Therefore, applications to network solids (ionic network crystals, metallic crystals, coordination polymers, organic polymers) will not be ideal. Nevertheless, using a supermolecule approach, this has been attempted (Jayatilaka, 1998 ▶; Hudák *et al.*, 2010 ▶). It would be better to use a fully periodic wavefunction to obtain the crystal ED.

(iii) *The crystal field*. Related to this is the treatment of intermolecular contacts, such as secondary coordination, hydrogen or halogen bonds. The present implementation of HAR takes into account ED polarization using a charge and dipole crystal field which may not be adequate. It would be better to include the EDs from near-neighbour molecules directly into the calculation for the ED of the central molecule where Hirshfeld atoms are extracted. As a matter of fact, such an approach can be extended to a fully periodic wavefunction and ED (Shukla *et al.*, 1998 ▶).

(iv) *Non-uniqueness of the atomic partitioning*. Since we use molecular EDs, the Hirshfeld atoms on the periphery are qualitatively different: they are unbounded because they have no near-neighbours. The effect of this shape change in HAR could be tested and avoided by accounting for the near-neighbour atoms, *e.g.* through a molecular cluster approach (see above). Also, the Hirshfeld atom partitioning can be improved *via* a theoretically more appealing iterative self-consistent procedure (Bultinck *et al.*, 2009 ▶). This Hirshfeld-I procedure is also known to produce atomic charges which better reproduce the total electrostatic potential (van Damme *et al.*, 2009 ▶).

(v) **Z*′ > 1 and disorder treatments*. Perhaps the most important limitations of the current implementation of HAR are its restriction to one or less molecules in the asymmetric unit and the treatment of disorder. The former is not a fundamental problem and different approaches to address it are currently being implemented and tested by us (Woińska *et al.*, 2014 ▶). The treatment of any aperiodic disorder is more difficult within our periodic single-wavefunction cluster framework.

## 6. Conclusions   

Hirshfeld atom refinement (HAR) is a conceptually straightforward general-purpose method for the unconstrained X-ray refinement of all structural parameters, including H-atom positions and anisotropic displacement parameters. In this study, we have abolished the ‘rigid atom approximation’ and implemented a new iterative and automated HAR. The new scheme consists of a succession of iterations, each including repeated molecular electron density calculations, determination of aspherical atomic scattering factors using Hirshfeld atoms, and structural least-squares refinement, all cycled to convergence.

We have performed HAR on the dipeptide Gly–l-Ala using synchrotron X-ray data measured at multiple temperatures (12, 50, 100, 150, 220 and 295 K) and compared the structural results of four of those measurements with results from neutron diffraction measurements (12, 50, 150 and 295 K).

We have shown that, for this molecule with 20 atoms, ten of which are H atoms, and without inherent symmetry, the HAR calculations are feasible within a few hours. Overall the structural results show a high precision and agree well with the results from neutron diffraction. HAR produces these results using X-ray data with moderate resolutions which are today routinely achieved for small molecules, *d*_max_ = 0.65 Å.

Our results for Gly–l-Ala establish that a triple-zeta basis set such as cc-pVTZ is sufficient to obtain basis-set converged results for the structural parameters. To determine the positions of all atoms, H and non-H, even a double-zeta basis set is sufficient.

In most cases, the BLYP density functional produces better agreement with the neutron measurements, although for the bond lengths involving H atoms the BLYP functional produces comparatively short bond lengths and HF calculations comparatively long ones. It would thus be worthwhile to check if hybrid density functionals (which contain an admixture of HF theory) give better results, thereby providing a potent test for the performance of different quantum mechanical methods.

In summary, the use of the BLYP/cc-pVTZ level of theory is recommended for HAR of organic molecules.

It is remarkable that H-atom positions, interatomic distances involving H atoms and H-atom ADPs, which so far have not been routinely obtainable from X-ray diffraction experiments, have been determined from HAR in agreement with the results from the corresponding neutron diffraction measurements and with a precision that is the same or even better.

Specifically, at temperatures of 150 K and lower, the N—H and C—H bond lengths show mean absolute differences between HAR and neutron-derived values that are no larger than 0.009 Å (agreement within ∼2 csu’s). Considering only the C—H bonds the agreement is even better, with absolute differences of ∼0.004 Å at all temperatures. This observation also holds for intermolecular hydrogen bond lengths.

Visual inspection of the H-atom ADPs from HAR shows that they are of the same size and orientation as those from neutron diffraction and are therefore physically reasonable. The mean absolute differences in the H-atom ADPs (at 150 K and lower) are between 0.004 and 0.006 Å^2^ (agreement within less than 2 csu’s).

All of these results are very promising. Nevertheless, some problems remain: the non-H atom ADPs have indicated systematic differences between the two measurements, attributable to experimental errors. After eliminating these errors, even better results should be achievable.

Our main priority now is to work on further improvements of HAR to allow applications to compounds with more than one molecule in the asymmetric unit. We believe that in the future, with more computational power and improvements in the algorithms and software, HAR has the potential to replace the IAM or pseudo-atom database approaches and to become the default standard for chemical crystallography.

## Supplementary Material

Extra figures and tables. DOI: 10.1107/S2052252514014845/fc5002sup1.pdf

Crystallographic information files (CIFs) and validation reports for the X-ray HAR and neutron refinements. DOI: 10.1107/S2052252514014845/fc5002sup2.zip

CCDC references: 995876, 995877, 995878, 995879, 995880, 995881

## Figures and Tables

**Table 1:** Comparison of results for NH_3_ from a multipole refinement with constrained hydrogen parameters, an X-ray constrained wavefunction (XCW) fitting on the fixed final geometry from the multipole refinement, and an iterative Hirshfeld atom refinement (HAR) without any constraints: χ^2^ agreement statistics, N—H bond length (Å), fractional coordinates and ADPs (Å^2^)

	Multipoles	XCW	HAR
χ^2^	0.8	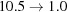 †	0.6
*r*(N—H)	1.010	1.010	0.987 (5)
N *x*	0.2103 (1)	0.2103	0.21059 (5)
H *x*	0.3722	0.3722	0.3668 (9)
H *y*	0.2627	0.2627	0.269 (1)
H *z*	0.1113	0.1113	0.1148 (8)
N *U*_11_	0.0372 (2)	0.0372	0.0363 (2)
N *U*_12_	−0.0009 (1)	−0.0009	−0.00163 (9)
H *U*_11_	0.0053	0.0053	0.070 (3)
H *U*_22_			0.066 (3)
H *U*_33_			0.064 (3)
H *U*_12_			−0.016 (3)
H *U*_13_			0.006 (2)
H *U*_23_			−0.009 (3)

† χ^2^ values before and after X-ray constrained wavefunction fitting.

**Table 2:** Experimental details for Gly–L-Ala in the orthorhombic space group *P*2_1_2_1_2_1_

		X-ray	Neutron			X-ray	Neutron
Crystal data†				Data collection‡			
*a* (Å)	12 K	7.4583 (4)	7.4541 (15)	No. of reflections§	12 K	8531/–/8078¶	2237/1960/1692
	50 K	7.462 (1)	7.4587 (7)		50 K	8714/–/8031	1467/1354/1205
	100 K	7.472 (2)	–		100 K	12 998/–/12 386	–
	150 K	7.487 (2)	7.4871 (16)		150 K	13 196/–/12 339	1481/1354/1165
	220 K	7.5071 (6)	–		220 K	13 350/–/12 005	–
	295 K	7.529 (2)	7.5302 (11)		295 K	11 998/–/10 542	1446/1354/1048
*b* (Å)	12 K	9.4892 (7)	9.4918 (19)	*R*_int_	12 K	0.0253	0.0289
	50 K	9.490 (2)	9.4928 (9)		50 K	0.0301	0.0240
	100 K	9.4907 (6)	–		100 K	0.0297	–
	150 K	9.496 (1)	9.4966 (19)		150 K	0.0262	0.0240
	220 K	9.5023 (2)	–		220 K	0.0281	–
	295 K	9.516 (1)	9.5115 (16)		295 K	0.0280	0.0254
*c* (Å)	12 K	9.7301 (6)	9.7287 (19)	Completeness (%)	12 K	0.969	0.998
	50 K	9.727 (1)	9.7250 (9)		50 K	0.938	0.996
	100 K	9.7169 (8)	–		100 K	0.974	–
	150 K	9.7099 (4)	9.7078 (19)		150 K	0.967	0.996
	220 K	9.699 (1)	–		220 K	0.962	–
	295 K	9.688 (1)	9.6855 (14)		295 K	0.959	0.986
*V* (Å^3^)	12 K	688.63 (8)	688.3 (2)	Diffractometer		ID11 at ESRF, Bruker SMART CCD	D9 at ILL, ^3^He position-sensitive
	50 K	688.81 (17)	688.57 (11)				
	100 K	689.1 (2)	–	Wavelength (Å)		0.5259 (2)	0.83130
	150 K	690.34 (19)	690.2 (2)	Resolution *d*_max_ (Å)		0.657	0.646–0.675††
	220 K	691.88 (9)	–	Crystal size (mm^3^)		0.10 × 0.08 × 0.05	3.0 × 3.0 × 1.5
	295 K	694.1 (2)	693.71 (18)	Criterion for observation		*F* > 3σ(*F*)	*I* > 2σ(*I*)

† Chemical formula: C_5_H_10_N_2_O_3_; *M*_r_ = 146.15; *Z* = 4.

‡ Absorption and extinction corrections only applied to the neutron measurements.

§ HAR was performed against the unmerged data set, so the number of unique reflections is irrelevant and ‘observed’ refers to the number of reflections after pruning the unmerged data set according to the criterion for observation. Redundancies are 5.82 (12 K), 6.14 (50 K), 8.72 (100 K), 8.82 (150 K), 8.88 (220 K), 8.03 (295 K).

¶ Measured/unique/observed.

†† The values vary slightly for the four different temperatures: 0.646 (12 K), 0.675 (50 K), 0.675 (150 K), 0.674 (295 K).

**Table 3:** Comparison of χ^2^ agreement statistics for different Hirshfeld atom refinement (HAR) models at the different temperatures

		Basis set		
*T* (K)	Method	cc-pVDZ	cc-pVTZ	cc-pVQZ
12	HF	1.2566	1.2299	1.2303
	BLYP	1.1674	1.1056	1.1006
50	HF	1.1133	1.0840	1.0818
	BLYP	1.0218	0.9620	0.9583
150	HF	1.3364	1.3124	1.3139
	BLYP	1.2166	1.1631	1.1609
295	HF	1.3163	1.2948	1.2948
	BLYP	1.2074	1.1884	1.1884

**Table 4:** Mean absolute differences (MADs) for H-atom fractional coordinates 〈|Δ*X*|〉 and corresponding population standard deviations σ_pop_; No. of data averaged = 30

		cc-pVDZ		cc-pVTZ		cc-pVQZ	
*T* (K)	Method	〈|Δ*X*|〉	σ_pop_	〈|Δ*X*|〉	σ_pop_	〈|Δ*X*|〉	σ_pop_
12	HF	0.001107	0.000800	0.001153	0.000763	0.001160	0.000777
	BLYP	0.000923	0.000760	0.000990	0.000683	0.001023	0.000648
50	HF	0.001353	0.000977	0.001377	0.000999	0.001270	0.000888
	BLYP	0.000903	0.000775	0.000833	0.000692	0.000760	0.000555
150	HF	0.001283	0.000952	0.001360	0.000935	0.001390	0.000894
	BLYP	0.001187	0.000891	0.001270	0.000826	0.001227	0.000797
295	HF	0.001427	0.001038	0.001403	0.001004	0.001370	0.001049
	BLYP	0.001353	0.001039	0.001350	0.001052	0.001350	0.001052

**Table 5:** Bond lengths *d*_*D*—H_ (Å) involving an H atom from HAR using the BLYP/cc-pVTZ model, compared with neutron measurements The last entry for every bond type refers to average values from neutron diffraction given by Allen & Bruno (2010 ▶). These authors use temperature intervals of *T* ≤ 60 K, 60 ≤ *T* ≤ 140 K, and *T* ≥ 240 K. We use the value from the middle range for comparison with our 150 K values. The errors in brackets refer to su’s for neutron and X-ray entries, but to σ_pop_ values for neutron reference values (Allen & Bruno, 2010 ▶).

	12 K		50 K		150 K		295 K	
Bond	Neutron	X-ray	Neutron	X-ray	Neutron	X-ray	Neutron	X-ray
N1—H1N1	1.023 (4)	1.012 (3)	1.018 (4)	1.016 (4)	1.025 (4)	1.017 (2)	1.024 (6)	1.011 (3)
*Z*_2_N—H	1.020 (10)	–	1.020 (10)	–	1.019 (13)	–	1.011 (20)	–

N2—H1N2	1.044 (4)	1.038 (3)	1.045 (4)	1.051 (4)	1.041 (5)	1.042 (2)	1.042 (8)	1.031 (3)
N2—H2N2	1.045 (5)	1.042 (3)	1.042 (4)	1.046 (3)	1.052 (5)	1.037 (3)	1.043 (7)	1.034 (3)
N2—H3N2	1.044 (5)	1.027 (3)	1.042 (4)	1.029 (3)	1.040 (5)	1.022 (2)	1.049 (7)	1.014 (3)
〈*d*_N2—H_〉	1.044	1.036	1.043	1.042	1.044	1.034	1.045	1.026
N^+^—H	–	–	–	–	1.040 (10)	–	1.034 (16)	–

C2—H2	1.095 (5)	1.102 (3)	1.096 (5)	1.106 (4)	1.102 (6)	1.106 (2)	1.098 (8)	1.110 (3)
*Z*_3_C—H	1.101 (6)	–	1.101 (6)	–	1.099 (7)	–	1.099 (10)	–

C5—H5*A*	1.109 (5)	1.101 (3)	1.090 (5)	1.104 (4)	1.088 (6)	1.097 (2)	1.089 (8)	1.083 (3)
C5—H5*B*	1.102 (5)	1.084 (3)	1.106 (5)	1.090 (3)	1.101 (5)	1.089 (2)	1.093 (8)	1.076 (3)
〈*d*_C5—H_〉	1.106	1.093	1.098	1.097	1.095	1.093	1.091	1.080
*Z*_2_C—H_2_	1.097 (10)	–	1.097 (10)	–	1.097 (6)	–	1.087 (16)	–

C3—H3*A*	1.097 (6)	1.091 (3)	1.081 (6)	1.092 (4)	1.088 (8)	1.085 (3)	1.076 (5)	1.072 (5)
C3—H3*B*	1.093 (6)	1.099 (3)	1.093 (6)	1.092 (3)	1.075 (7)	1.092 (3)	1.072 (1)	1.077 (5)
C3—H3*C*	1.082 (6)	1.079 (3)	1.092 (5)	1.077 (3)	1.090 (6)	1.090 (3)	1.084 (9)	1.072 (4)
〈*d*_C3—H_〉	1.091	1.090	1.089	1.087	1.084	1.089	1.077	1.074
*Z*C—H_3_	1.088 (9)	–	1.088 (9)	–	1.084 (13)	–	1.055 (36)	–

**Table 6:** Various measures of *D*—H bond-length differences (Å) for the cc-pVTZ basis set at several temperatures and both HF and BLYP methods Mean absolute differences (MADs) 〈|Δ*d*|〉 with corresponding σ_pop_; mean differences (MDs) 〈Δ*d*〉 with corresponding σ_pop_; csu-weighted root mean-square differences (wRMSDs) 〈[Δ *d*/csu(*d*)]^2^〉^1/2^. No. of data averaged = 10.

*T*	Method	〈|Δ*d*|〉	σ_pop_	〈Δ*d*〉	σ_pop_	〈[Δ*d*/csu]^2^〉^1/2^
12 K	HF	0.008418	0.004518	0.008148	0.009554	1.712758
	BLYP	0.008539	0.005132	−0.006019	0.009963	1.775959
50 K	HF	0.014082	0.010326	0.014082	0.017462	2.985122
	BLYP	0.009252	0.005225	−0.000188	0.010626	1.802856
150 K	HF	0.012666	0.006950	0.011659	0.014448	2.406803
	BLYP	0.008670	0.006168	−0.002521	0.010640	1.791486
295 K	HF	0.008863	0.004913	0.003918	0.010133	1.251866
	BLYP	0.012457	0.008435	−0.008935	0.015044	1.906538

**Table 7:** Geometry of hydrogen bonds (Å, °) derived from the 12 K neutron experiment (first row), compared with the 12 K HAR refinement results at the BLYP/cc-pVTZ level of theory (second row) For *D*—H distances see Table 5 ▶.

Bond	H⋯*A*	*D*⋯*A*	*D*—H⋯*A*	Symmetry codes
N1—H1N1⋯O1	1.869 (5)	2.876 (2)	167.8 (4)	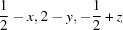
	1.874 (6)	2.8746 (3)	169.5 (5)	
N2—H1N2⋯O2	1.712 (5)	2.747 (3)	170.8 (4)	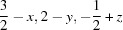
	1.718 (6)	2.7490 (3)	171.1 (6)	
N2—H2N2⋯O1	1.686 (5)	2.716 (2)	167.6 (4)	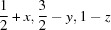
	1.690 (6)	2.7166 (3)	167.8 (5)	
N2—H3N2⋯O2	1.728 (5)	2.723 (2)	157.6 (4)	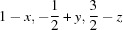
	1.747 (6)	2.7249 (3)	157.8 (6)	
C3—H3*C*⋯O3	2.406 (5)	3.465 (3)	166.1 (5)	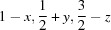
	2.401 (6)	3.4623 (3)	167.7 (5)	
C5—H5*B*⋯O3	2.475 (5)	3.555 (3)	166.3 (4)	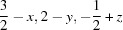
	2.494 (5)	3.5557 (4)	166.3 (5)	

**Table 8:** Various measures of non-H-atom ADP differences for the cc-pVTZ basis set at several temperatures and both HF and BLYP methods Mean absolute differences (MADs) 〈|Δ*U*_*ij*_|〉 with corresponding σ_pop_, both in Å^2^; mean differences (MDs) 〈Δ*U*_*ij*_〉 with corresponding σ_pop_, both in Å^2^; csu-weighted root-mean-square differences (wRMSDs) 〈[Δ*U*_*ij*_/csu(*U*_*ij*_)]^2^〉^1/2^; mean ratios (MRs) for diagonal ADPs 〈*r*〉 = 〈*U*_*ii*_(X-ray)/*U*_*ii*_(neutron)〉 with corresponding σ_pop_. No. of data averaged = 60.

*T* (K)	Method	〈|Δ*U*_*ij*_|〉	σ_pop_	〈Δ*U*_*ij*_〉	σ_pop_	〈[Δ*U*_*ij*_/csu]^2^〉^1/2^	〈r〉	σ_pop_
12	HF	0.001056	0.001045	−0.000815	0.001485	2.525565	0.750579	0.166088
	BLYP	0.001153	0.001136	−0.000905	0.001619	2.734121	0.711751	0.177196
50	HF	0.001152	0.001213	−0.000880	0.001673	2.589051	0.788322	0.163865
	BLYP	0.001194	0.001290	−0.000951	0.001758	2.686887	0.763786	0.164968
150	HF	0.001137	0.001163	−0.000768	0.001627	2.211385	0.895412	0.092325
	BLYP	0.001249	0.001194	−0.000853	0.001727	2.326422	0.879288	0.095122
295	HF	0.001336	0.001072	−0.000784	0.001713	1.486372	0.966640	0.062434
	BLYP	0.001357	0.001008	−0.000759	0.001691	1.415492	0.967150	0.065682

**Table 9:** Various measures of H-atom ADP differences for the cc-pVTZ basis set at several temperatures and with both HF and BLYP methods Mean absolute differences (MADs) 〈|Δ*U*_*ij*_|〉 with corresponding σ_pop_, both in Å^2^; mean differences (MDs) 〈Δ*U*_*ij*_〉 with corresponding σ_pop_, both in Å^2^; csu-weighted root-mean-square differences (wRMSDs) 〈[Δ*U*_*ij*_/csu(*U*_*ij*_)]^2^〉^1/2^; mean ratios (MRs) for diagonal ADPs 〈*r*〉 = 〈*U*_*ii*_(X-ray)/*U*_*ii*_(neutron)〉 with corresponding σ_pop_. No. of data averaged = 60.

*T* (K)	Method	〈|Δ*U*_*ij*_|〉	σ_pop_	〈Δ*U*_*ij*_〉	σ_pop_	〈[Δ*U*_*ij*_/csu]^2^〉^1/2^	〈*r*〉	σ_pop_
12	HF	0.004993	0.003702	−0.000050	0.006216	1.712070	1.013665	0.334025
	BLYP	0.004653	0.004217	−0.000267	0.006280	1.830323	0.971171	0.318888
50	HF	0.005000	0.004246	0.000003	0.006560	1.493656	0.988599	0.326270
	BLYP	0.004003	0.003443	−0.000680	0.005281	1.292494	0.941492	0.313424
150	HF	0.004850	0.003663	0.000003	0.006078	1.525596	1.013072	0.180484
	BLYP	0.003663	0.003133	−0.000480	0.004820	1.426491	0.989109	0.145564
295	HF	0.009683	0.007758	0.002383	0.012408	1.780602	1.078869	0.214357
	BLYP	0.007450	0.005795	0.001283	0.009438	1.607518	1.048397	0.148622

## Footnotes

1 The term ‘rigid atom approximation’ is proposed in the present context to distinguish between the original HAR and the iterative HAR versions. It is completely unrelated to the term ‘rigid pseudo-atom’ which means that atomic electron densities used in multipole modelling are not a function of atomic displacements.

2 In Morgenroth *et al.* (2008 ▶), Table 2 lists MAD values 〈|Δ*U*_*ij*_|〉, rather than MD values as might be expected from the column label 〈Δ*U*〉 given there. This was checked by reproducing some of the MAD values in their Table 2 from data given in the original literature.

3 Manual or automated iterations of HAR have been successfully applied in a number of other recent studies in the meantime (Dittrich *et al.*, 2012 ▶; Dittrich & Jayatilaka, 2012 ▶; Grabowsky *et al.*, 2012 ▶; Chęcińska *et al.*, 2013 ▶; Zakrzewska *et al.*, 2013 ▶; Hickstein *et al.*, 2013 ▶; Woińska *et al.*, 2014 ▶; Lübben *et al.*, 2014 ▶), but neither the background nor a validation has been published until now.
