# Supplementary material for: Hirshfeld atom refinement
Source: IUCrJ. 2014 Aug 29;1(Pt 5):361–79. doi: 10.1107/S2052252514014845 (PMC4174878; doi:10.1107/S2052252514014845)
Supplement: Supplementary file 1 [file m-01-00361-sup1.pdf]

IUCrJ

Volume 1 (2014)

Supporting information for article:

Hirshfeld atom refinement

Silvia C. Capelli, Hans-Beat Bürgi, Birger Dittrich, Simon Grabowsky and Dylan Jayatilaka

Supplementary Material:
"Hirshfeld Atom Refinement"

Silvia C. Capelli, Hans-Beat Bürgi, Birger Dittrich,
Simon Grabowsky, Dylan Jayatilaka

Contents

1 Figures of merit for the different HARs 2

2 Statistical data for non-hydrogen atom fractional coordinates 4

3 Statistical plots for non-hydrogen atom fractional coordinates 6

4 Statistical data for hydrogen atom fractional coordinates 12

5 Statistical plots for hydrogen atom fractional coordinates 14

6 Statistical data for non-hydrogen atom bond lengths 20

7 Statistical plots for non-hydrogen atom bond lengths 22

8 Statistical data for D-H bond lengths 28

9 Statistical plots for D-H bond lengths 30

10 Statistical data for non-hydrogen atom ADPs 36

11 Statistical plots for non-hydrogen atom ADPs 38

12 Statistical data for hydrogen atom ADPs 44

13 Statistical plots for hydrogen atom ADPs 46

14 Hirshfeld rigid-bond test 52

Tables: For the definitions of the statistical properties listed in the Tables, refer to section "Statistical analysis" in the main manuscript. In all tables, the properties are listed for the four temperatures 12, 50, 150 and 295 K (six temperatures in Tables S1-S3), all three basis sets and both quantum-mechanical methods HF and BLYP.

Figures: In all figures, csu-weighted differences between X-ray and neutron measurememts for the specified property are shown at the four different temperatures for the model ED specified. Rows 1 and 3: histograms with three entries per atom for coordinates (x, y, z), one entry per bond, or six entries per atom for ADPs (U_{11}, U_{22}, U_{33}, U_{12}, U_{13}, U_{23}). Property su's are shown for neutron (green) and X-ray (blue) measurements; the maximum su value is shown on the right axis. Rows 2 and 4: frequency plots.

1 Figures of merit for the different HARs

Table S1: Comparison of χ^2 agreement statistics for different Hirshfeld-atom refinement (HAR) models at all six different temperatures.

T	Method	Basis set		
		cc-pVDZ	cc-pVTZ	cc-pVQZ
12 K	HF	1.2566	1.2299	1.2303
	BLYP	1.1674	1.1056	1.1006
50 K	HF	1.1133	1.0840	1.0818
	BLYP	1.0218	0.9620	0.9583
100 K	HF	1.4047	1.3873	1.3914
	BLYP	1.3044	1.2455	1.2436
150 K	HF	1.3364	1.3124	1.3139
	BLYP	1.2166	1.1631	1.1609
220 K	HF	1.6401	1.6095	1.6063
	BLYP	1.5092	1.4627	failed
295 K	HF	1.3163	1.2948	1.2948
	BLYP	1.2074	1.1884	1.1884

Table S2: Comparison of R(F) agreement statistics for different Hirshfeld-atom refinement (HAR) models at all six different temperatures.

T	Method	Basis set		
		cc-pVDZ	cc-pVTZ	cc-pVQZ
12 K	HF	0.0204	0.0201	0.0201
	BLYP	0.0194	0.0188	0.0188
50 K	HF	0.0235	0.0231	0.0231
	BLYP	0.0224	0.0217	0.0217
100 K	HF	0.0220	0.0217	0.0218
	BLYP	0.0209	0.0203	0.0203
150 K	HF	0.0217	0.0215	0.0215
	BLYP	0.0205	0.0200	0.0199
220 K	HF	0.0247	0.0244	0.0244
	BLYP	0.0233	0.0229	N/A
295 K	HF	0.0255	0.0252	0.0252
	BLYP	0.0242	0.0239	0.0239

Table S3: Comparison of wR(F) agreement statistics for different Hirshfeld-atom refinement (HAR) models at all six different temperatures.

T	Method	Basis set		
		cc-pVDZ	cc-pVTZ	cc-pVQZ
12 K	HF	0.0247	0.0245	0.0245
	BLYP	0.0238	0.0232	0.0231
50 K	HF	0.0271	0.0267	0.0267
	BLYP	0.0259	0.0252	0.0251
100 K	HF	0.0261	0.0259	0.0259
	BLYP	0.0251	0.0245	0.0245
150 K	HF	0.0242	0.0240	0.0240
	BLYP	0.0231	0.0226	0.0226
220 K	HF	0.0279	0.0276	0.0276
	BLYP	0.0268	0.0263	N/A
295 K	HF	0.0259	0.0257	0.0257
	BLYP	0.0248	0.0246	0.0246

2 Statistical data for non-hydrogen atom fractional coordinates

Table S4: Mean absolute differences (MADs) of non-hydrogen atom fractional coordinates $\langle|\Delta X|\rangle$ and corresponding population standard deviations σ_{pop}. No. of data averaged: 30.

T	Method	cc-pVDZ $\langle\lvert\Delta X\rvert\rangle$	σ_{pop}	cc-pVTZ $\langle\lvert\Delta X\rvert\rangle$	σ_{pop}	cc-pVQZ $\langle\lvert\Delta X\rvert\rangle$	σ_{pop}
12K	HF	0.000155	0.000125	0.000158	0.000128	0.000158	0.000129
	BLYP	0.000161	0.000133	0.000163	0.000140	0.000163	0.000141
50K	HF	0.000204	0.000157	0.000214	0.000156	0.000216	0.000155
	BLYP	0.000201	0.000149	0.000208	0.000146	0.000209	0.000148
150K	HF	0.000176	0.000139	0.000174	0.000145	0.000175	0.000147
	BLYP	0.000165	0.000140	0.000163	0.000146	0.000164	0.000146
295K	HF	0.000244	0.000254	0.000242	0.000255	0.000245	0.000253
	BLYP	0.000249	0.000255	0.000250	0.000251	0.000250	0.000251

Table S5: Mean differences (MDs) for non-hydrogen atom fractional coordinates $\langle\Delta X\rangle$ and corresponding population standard deviations σ_{pop}. No. of data: averaged 30.

T	Method	cc-pVDZ $\langle\Delta X\rangle$	σ_{pop}	cc-pVTZ $\langle\Delta X\rangle$	σ_{pop}	cc-pVQZ $\langle\Delta X\rangle$	σ_{pop}
12K	HF	0.000018	0.000199	0.000016	0.000204	0.000015	0.000204
	BLYP	0.000023	0.000209	0.000019	0.000215	0.000021	0.000215
50K	HF	0.000084	0.000258	0.000084	0.000265	0.000084	0.000266
	BLYP	0.000090	0.000250	0.000088	0.000254	0.000089	0.000256
150K	HF	0.000072	0.000225	0.000073	0.000227	0.000072	0.000229
	BLYP	0.000071	0.000216	0.000069	0.000218	0.000070	0.000220
295K	HF	0.000039	0.000352	0.000039	0.000351	0.000041	0.000353
	BLYP	0.000032	0.000357	0.000028	0.000355	0.000028	0.000355

Table S6: Csu-weighted root-mean-square differences (wRMSDs) of non-hydrogen atom fractional coordinates $\left\langle [\Delta X/\mathrm{csu}(X)]^2 \right\rangle^{1/2}$. No. of data: averaged 30.

T	Method	cc-pVDZ	cc-pVTZ	cc-pVQZ
12K	HF	0.951879	0.968446	0.970630
	BLYP	0.997203	1.016120	1.020972
50K	HF	1.249446	1.285416	1.291801
	BLYP	1.244479	1.269376	1.280654
150K	HF	0.978945	1.004704	1.013880
	BLYP	0.947188	0.973824	0.981791
295K	HF	1.060981	1.078786	1.085922
	BLYP	1.065950	1.077062	1.077062

3 Statistical plots for non-hydrogen atom fractional coordinates

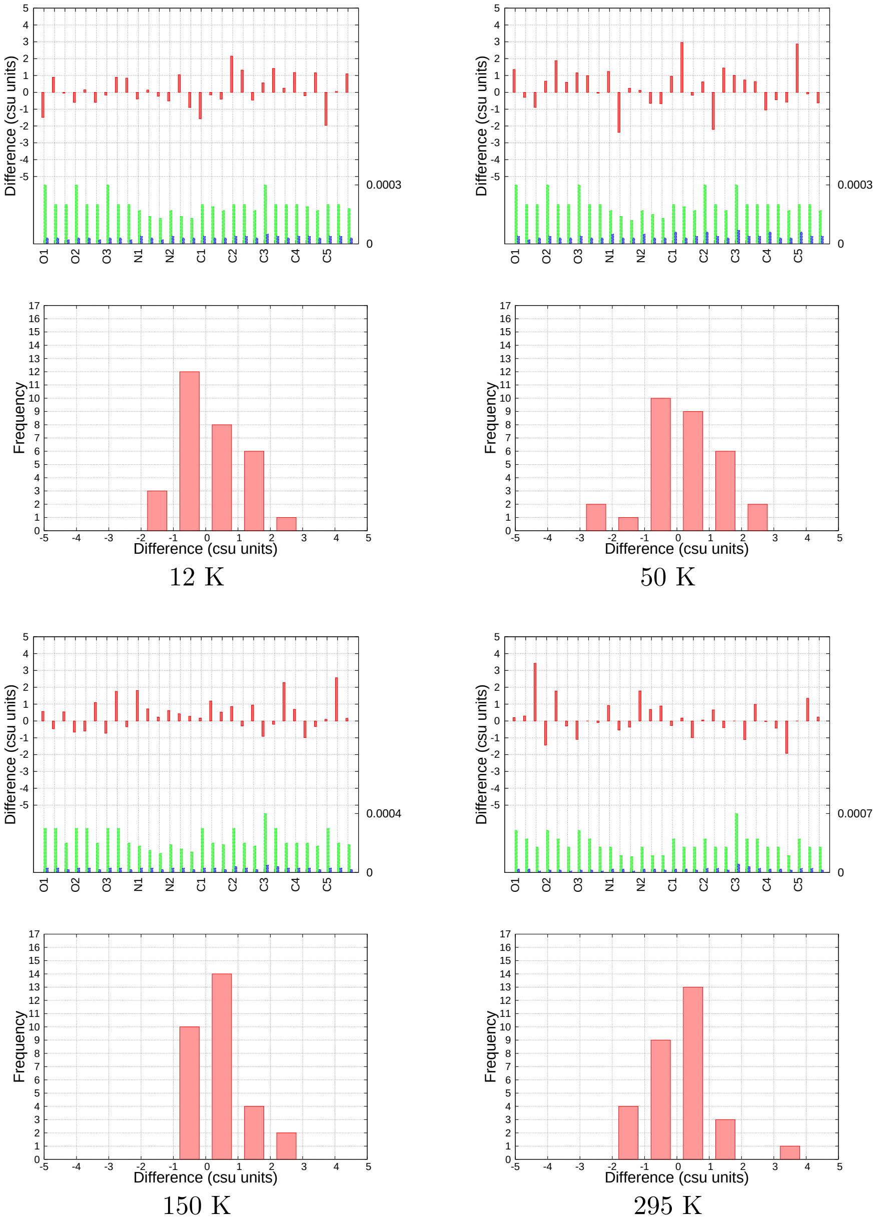

Figure S1: Non-hydrogen atom fractional coordinates, HF/cc-pVDZ

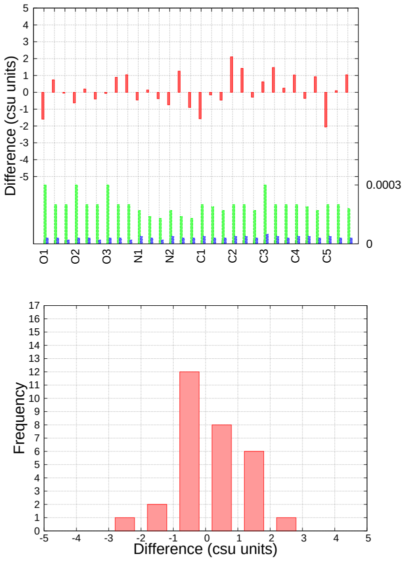

12 K

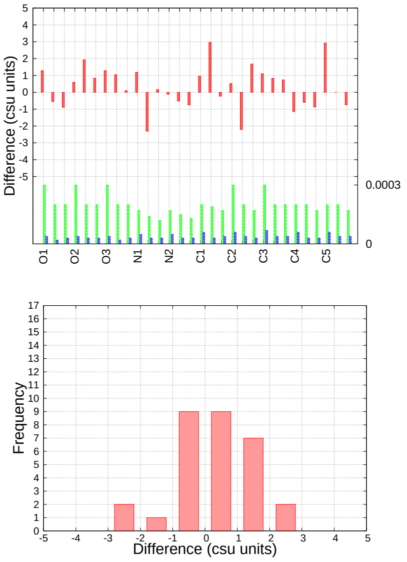

50 K

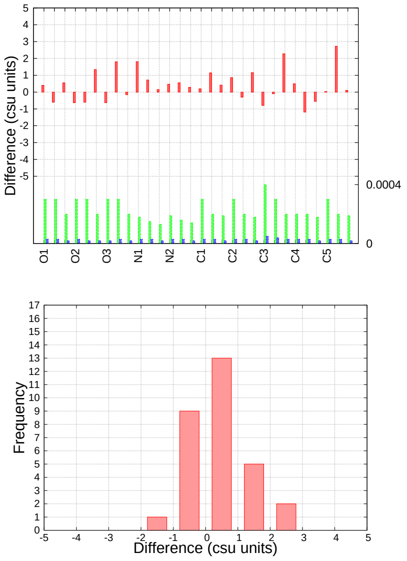

150 K

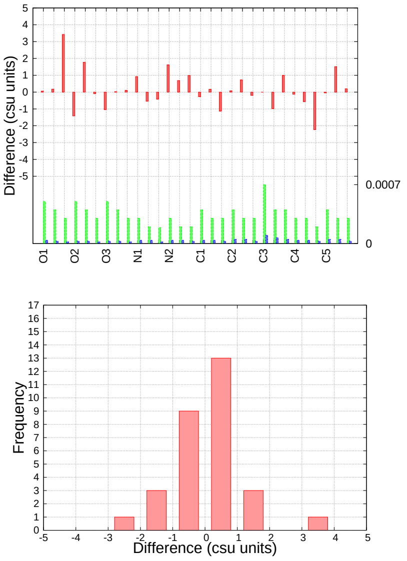

295 K

Figure S2: Non-hydrogen atom fractional coordinates, HF/cc-pVTZ

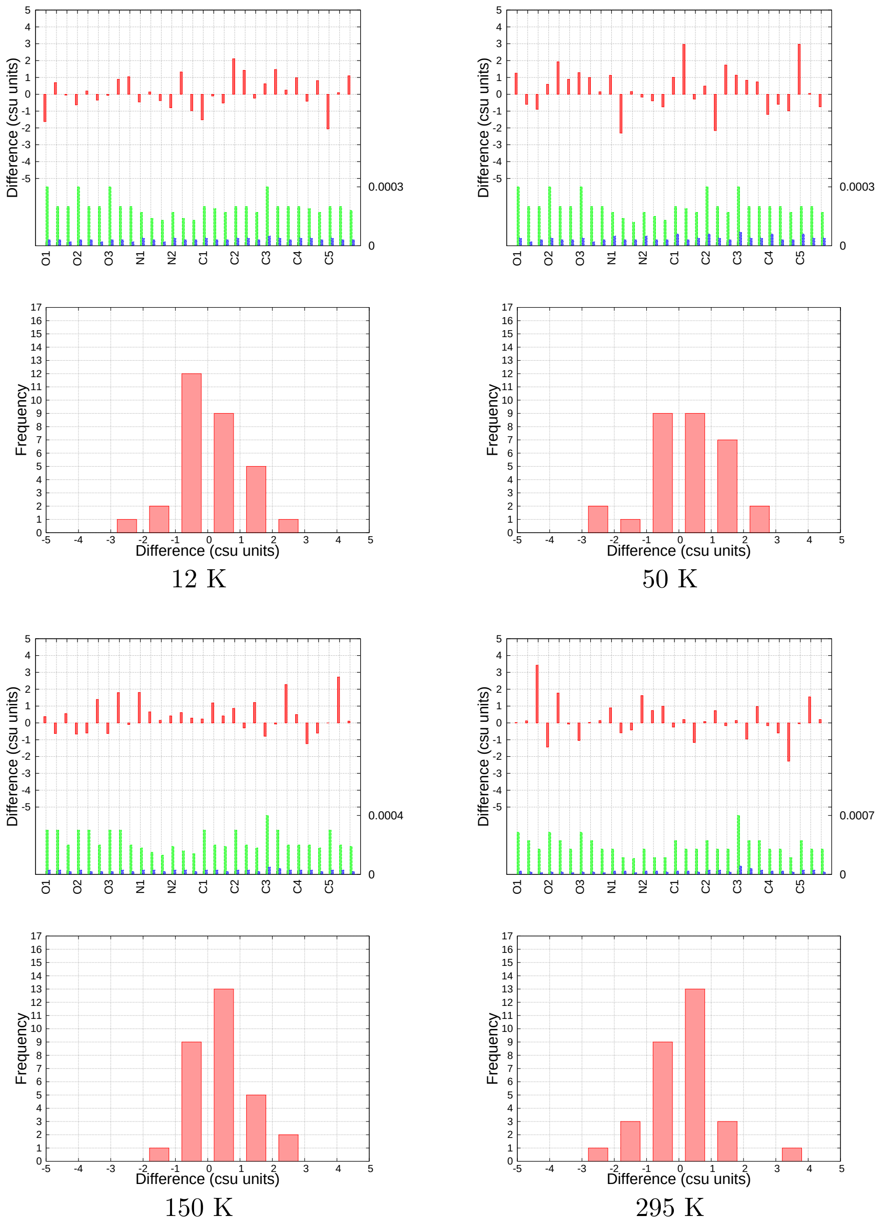

Figure S3: Non-hydrogen atom fractional coordinates, HF/cc-pVQZ

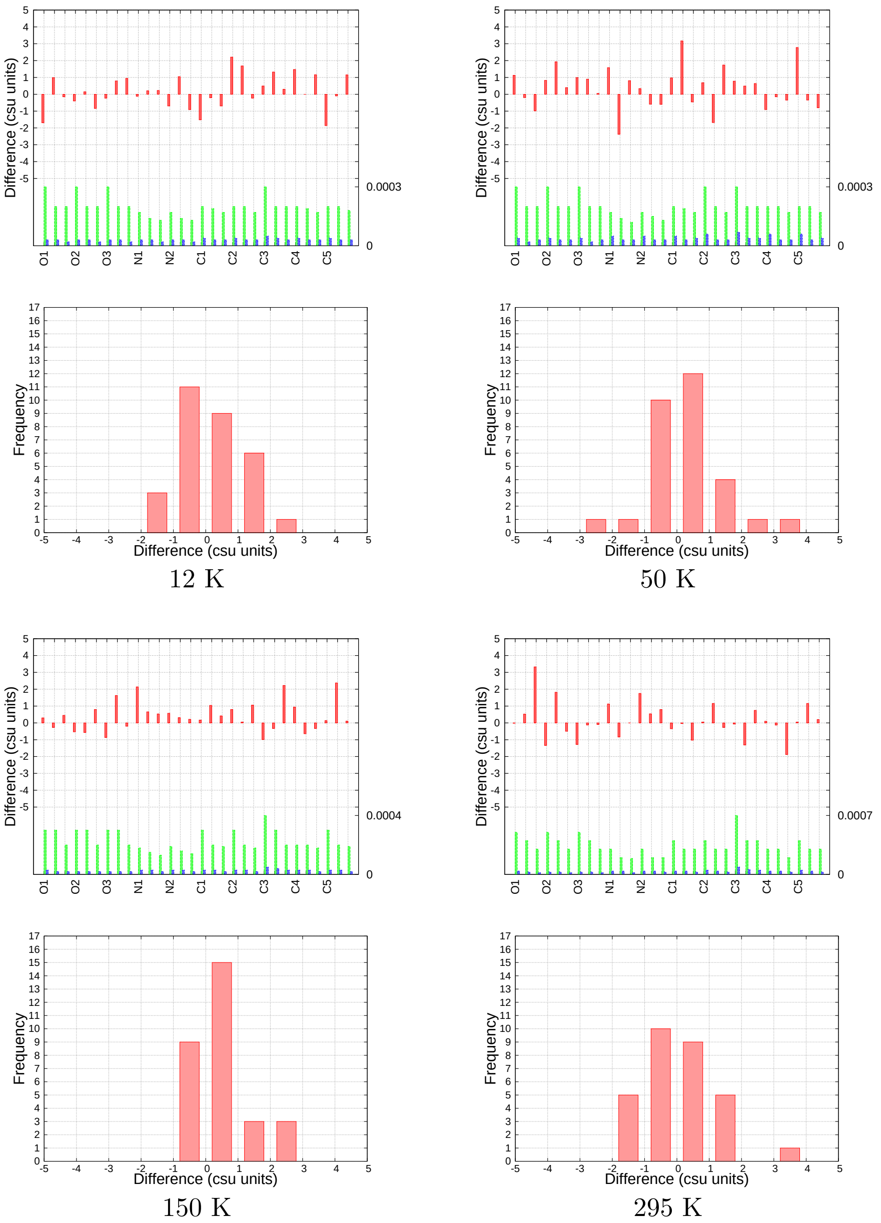

Figure S4: Non-hydrogen atom fractional coordinates, BLYP/cc-pVDZ

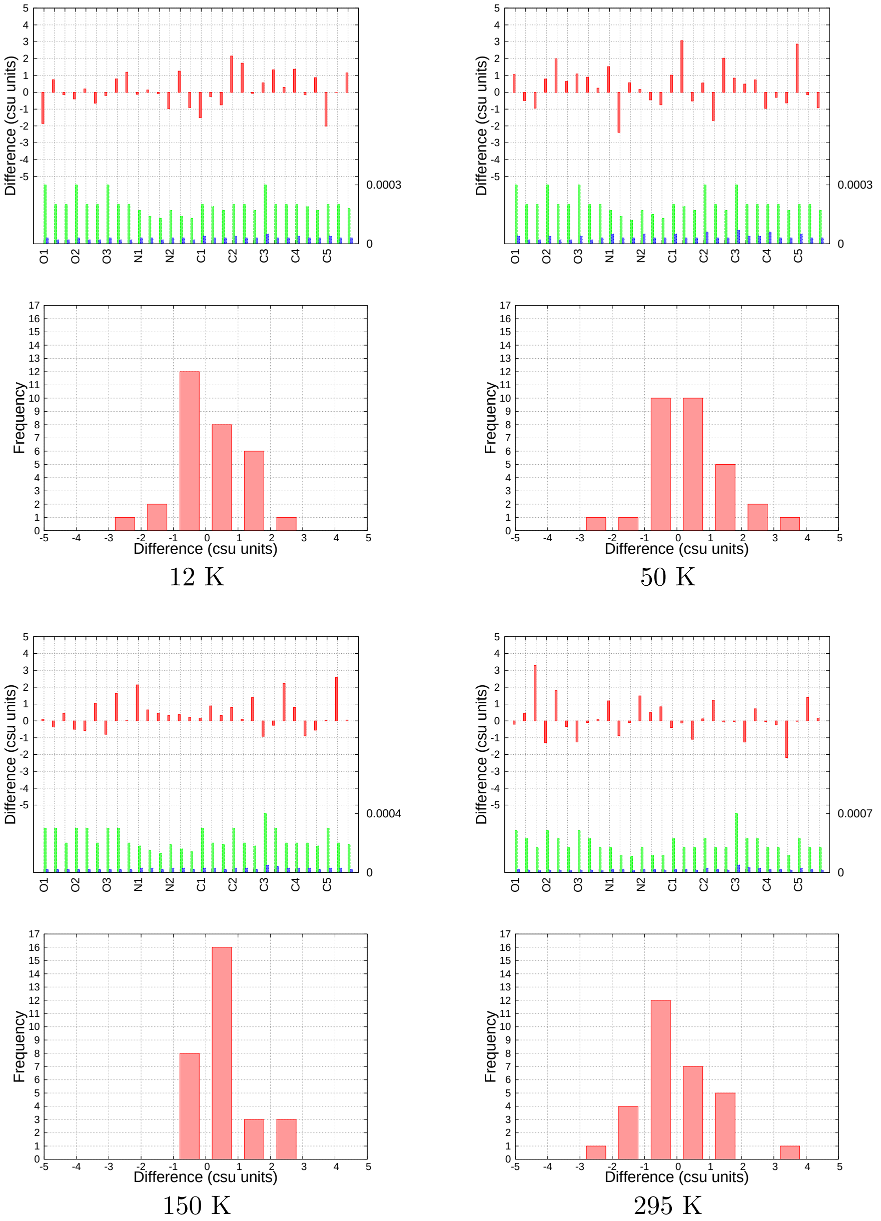

Figure S5: Non-hydrogen atom fractional coordinates, BLYP/cc-pVTZ

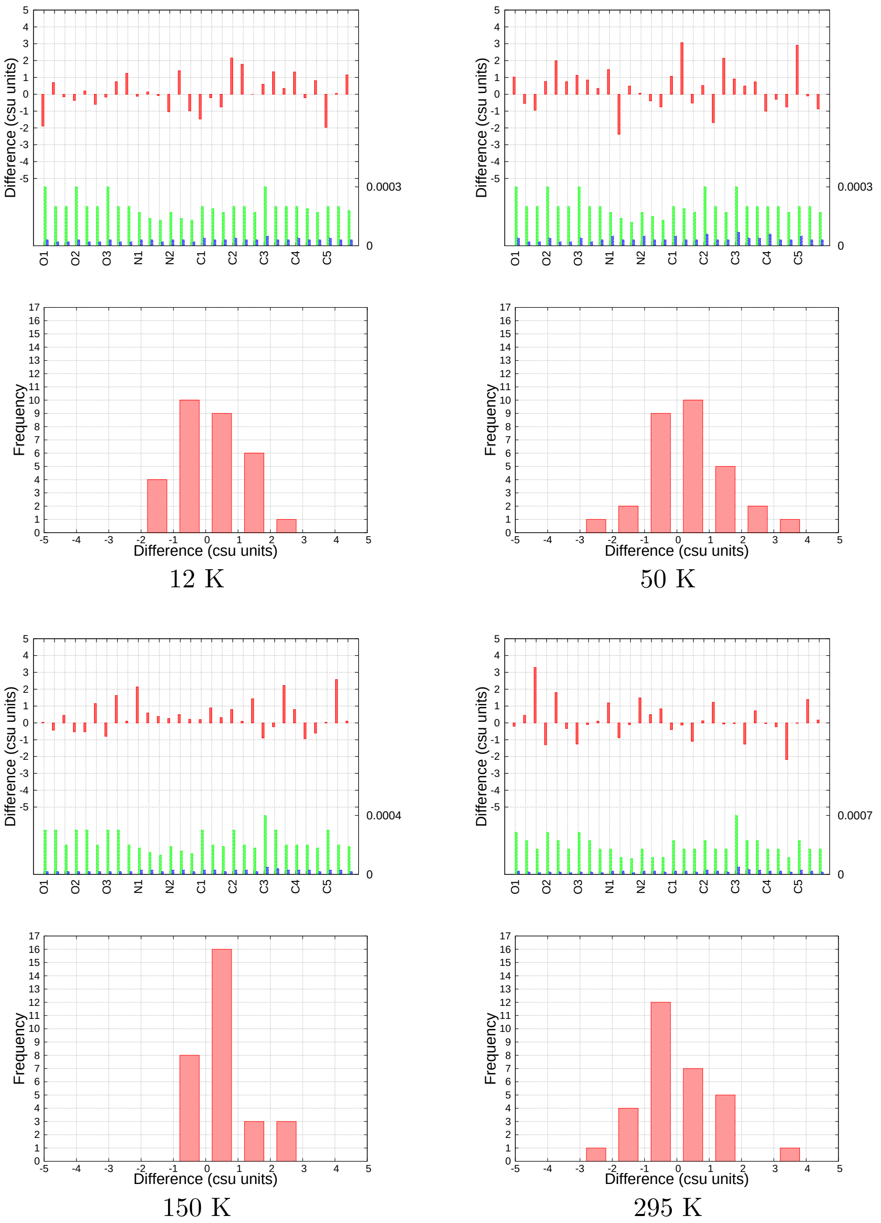

Figure S6: Non-hydrogen atom fractional coordinates, BLYP/cc-pVQZ

4 Statistical data for hydrogen atom fractional coordinates

Table S7: Mean absolute differences (MADs) of hydrogen atom fractional coordinates $\langle|\Delta X|\rangle$ and corresponding population standard deviations σ_{pop}. No. of data averaged: 30.

T	Method	cc-pVDZ $\langle|\Delta X|\rangle$	σ_{pop}	cc-pVTZ $\langle|\Delta X|\rangle$	σ_{pop}	cc-pVQZ $\langle|\Delta X|\rangle$	σ_{pop}
12K	HF	0.001107	0.000800	0.001153	0.000763	0.001160	0.000777
	BLYP	0.000923	0.000760	0.000990	0.000683	0.001023	0.000648
50K	HF	0.001353	0.000977	0.001377	0.000999	0.001270	0.000888
	BLYP	0.000903	0.000775	0.000833	0.000692	0.000760	0.000555
150K	HF	0.001283	0.000952	0.001360	0.000935	0.001390	0.000894
	BLYP	0.001187	0.000891	0.001270	0.000826	0.001227	0.000797
295K	HF	0.001427	0.001038	0.001403	0.001004	0.001370	0.001049
	BLYP	0.001353	0.001039	0.001350	0.001052	0.001350	0.001052

Table S8: Mean differences (MDs) for hydrogen atom fractional coordinates $\langle\Delta X\rangle$ and corresponding population standard deviations σ_{pop}. No. of data: averaged 30.

T	Method	cc-pVDZ $\langle\Delta X\rangle$	σ_{pop}	cc-pVTZ $\langle\Delta X\rangle$	σ_{pop}	cc-pVQZ $\langle\Delta X\rangle$	σ_{pop}
12K	HF	0.000020	0.001366	0.000107	0.001383	0.000127	0.001396
	BLYP	-0.000003	0.001196	0.000103	0.001203	0.000130	0.001211
50K	HF	-0.000227	0.001669	-0.000123	0.001701	-0.000137	0.001550
	BLYP	-0.000190	0.001190	-0.000113	0.001083	-0.000100	0.000941
150K	HF	-0.000037	0.001598	0.000047	0.001650	0.000070	0.001653
	BLYP	0.000040	0.001484	0.000190	0.001515	0.000207	0.001463
295K	HF	-0.000273	0.001764	-0.000217	0.001726	-0.000170	0.001726
	BLYP	-0.000073	0.001706	0.000063	0.001711	0.000063	0.001711

Table S9: Csu-weighted root-mean-square differences (wRMSDs) of hydrogen atom fractional coordinates $\left\langle [\Delta X/\mathrm{csu}(X)]^2 \right\rangle^{1/2}$. No. of data: averaged 30.

T	Method	cc-pVDZ	cc-pVTZ	cc-pVQZ
12K	HF	1.490959	1.544761	1.563969
	BLYP	1.314107	1.360913	1.376049
50K	HF	1.853681	1.815464	1.710207
	BLYP	1.345290	1.193593	1.071457
150K	HF	1.923555	1.974902	1.987612
	BLYP	1.742360	1.752998	1.709151
295K	HF	1.410538	1.427809	1.424014
	BLYP	1.354960	1.410249	1.410249

5 Statistical plots for hydrogen atom fractional coordinates

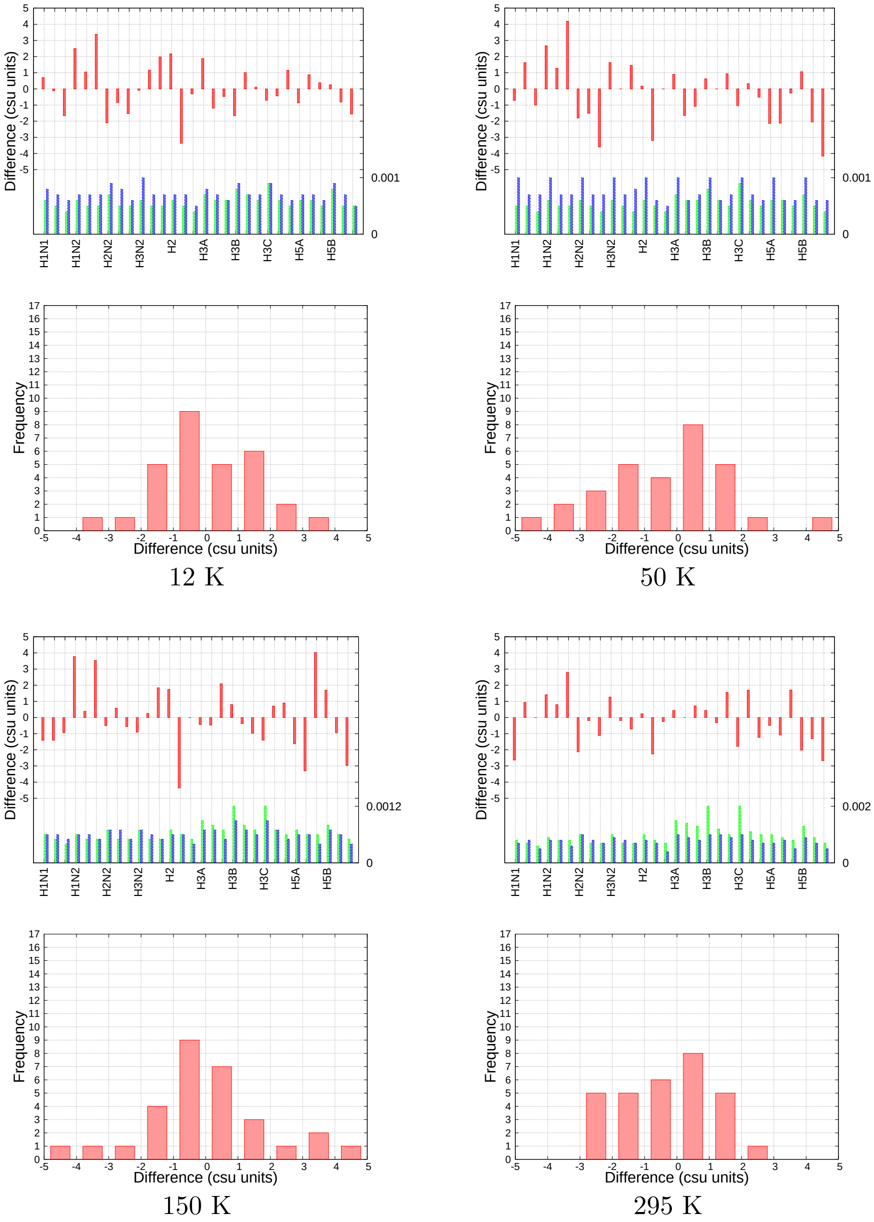

Figure S7: Hydrogen atom fractional coordinates, HF/cc-pVDZ

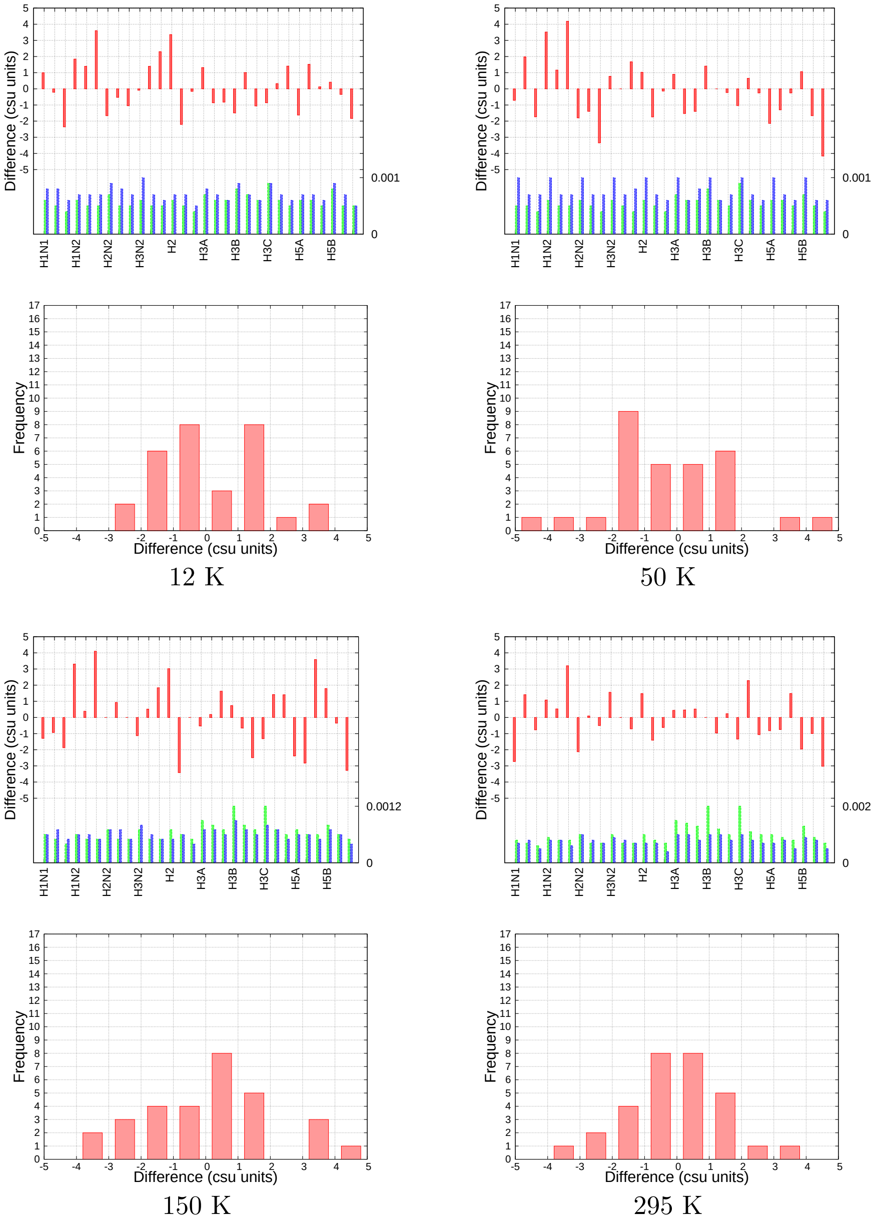

Figure S8: Hydrogen atom fractional coordinates, HF/cc-pVTZ

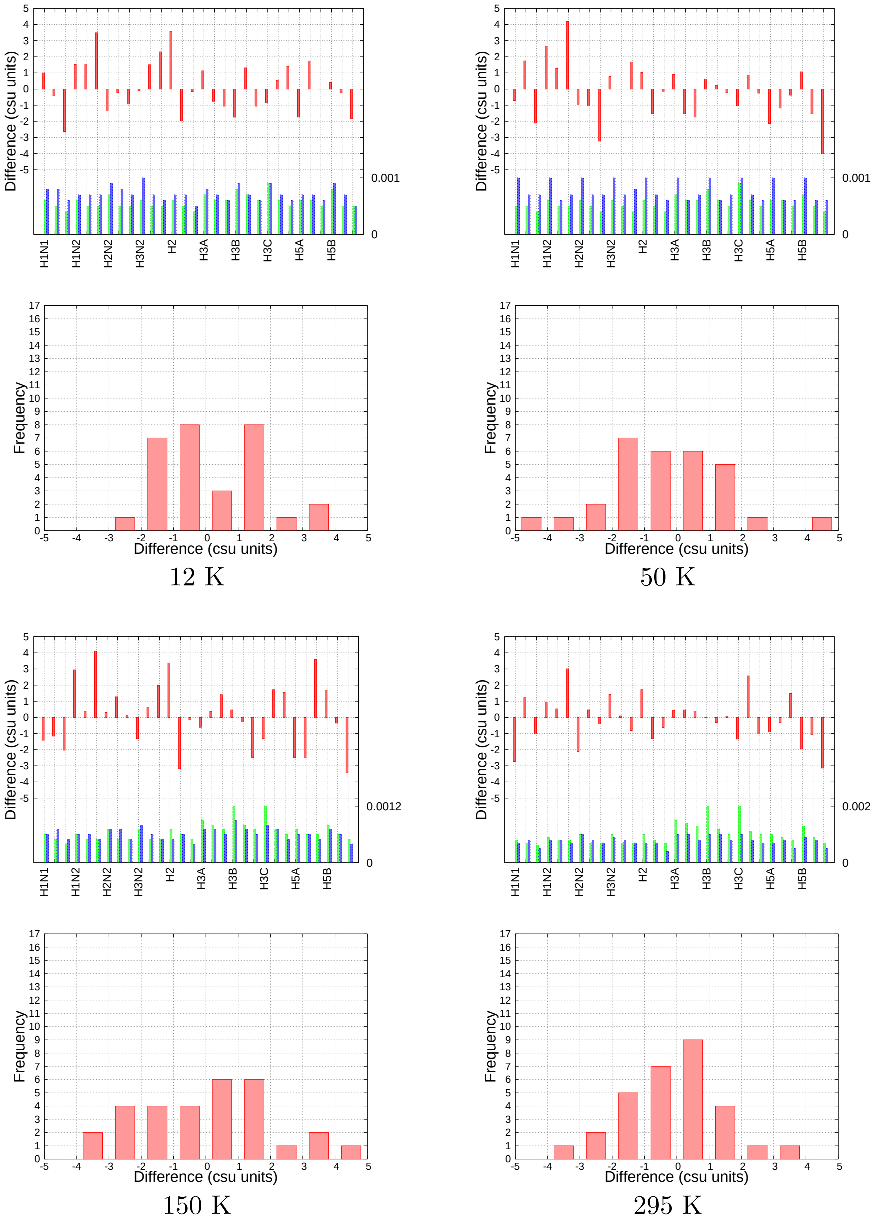

Figure S9: Hydrogen atom fractional coordinates, HF/cc-pVQZ

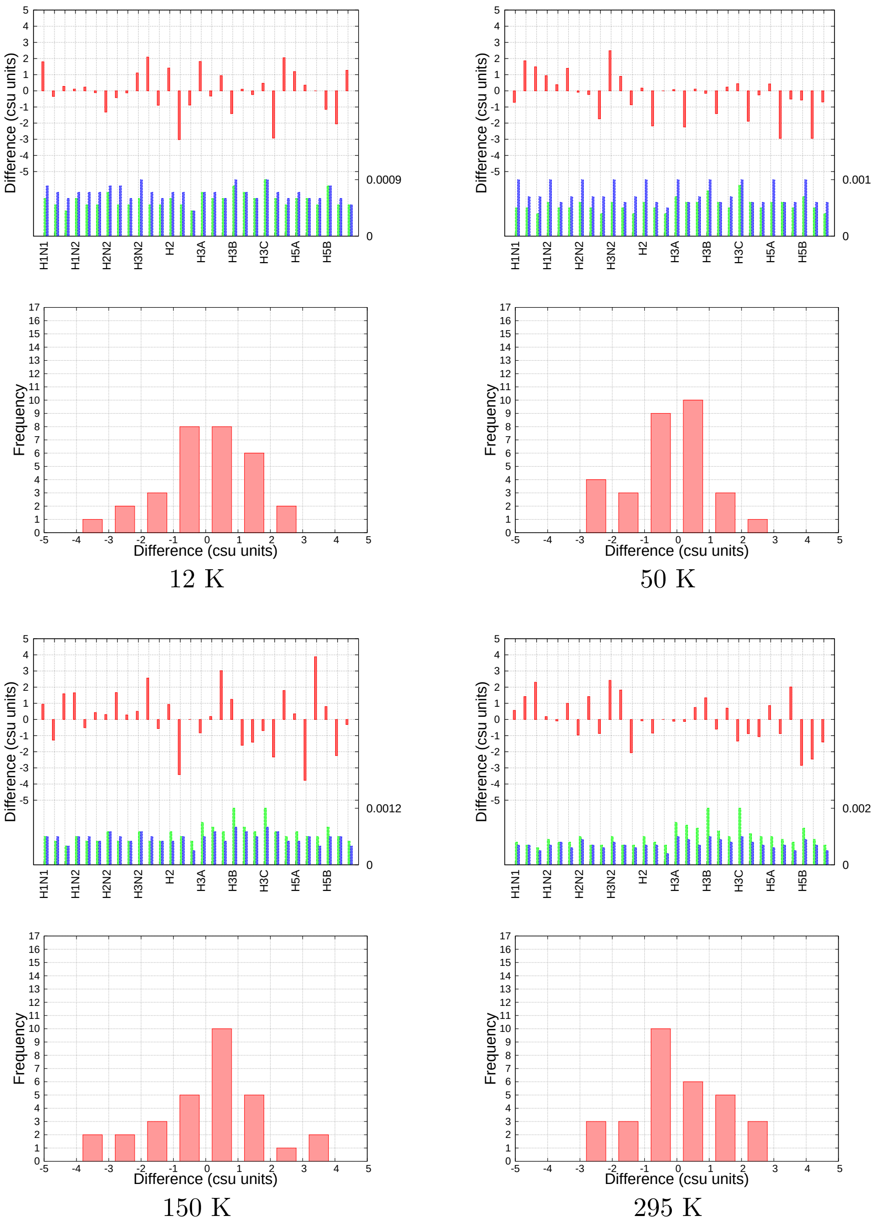

Figure S10: Hydrogen atom fractional coordinates, BLYP/cc-pVDZ

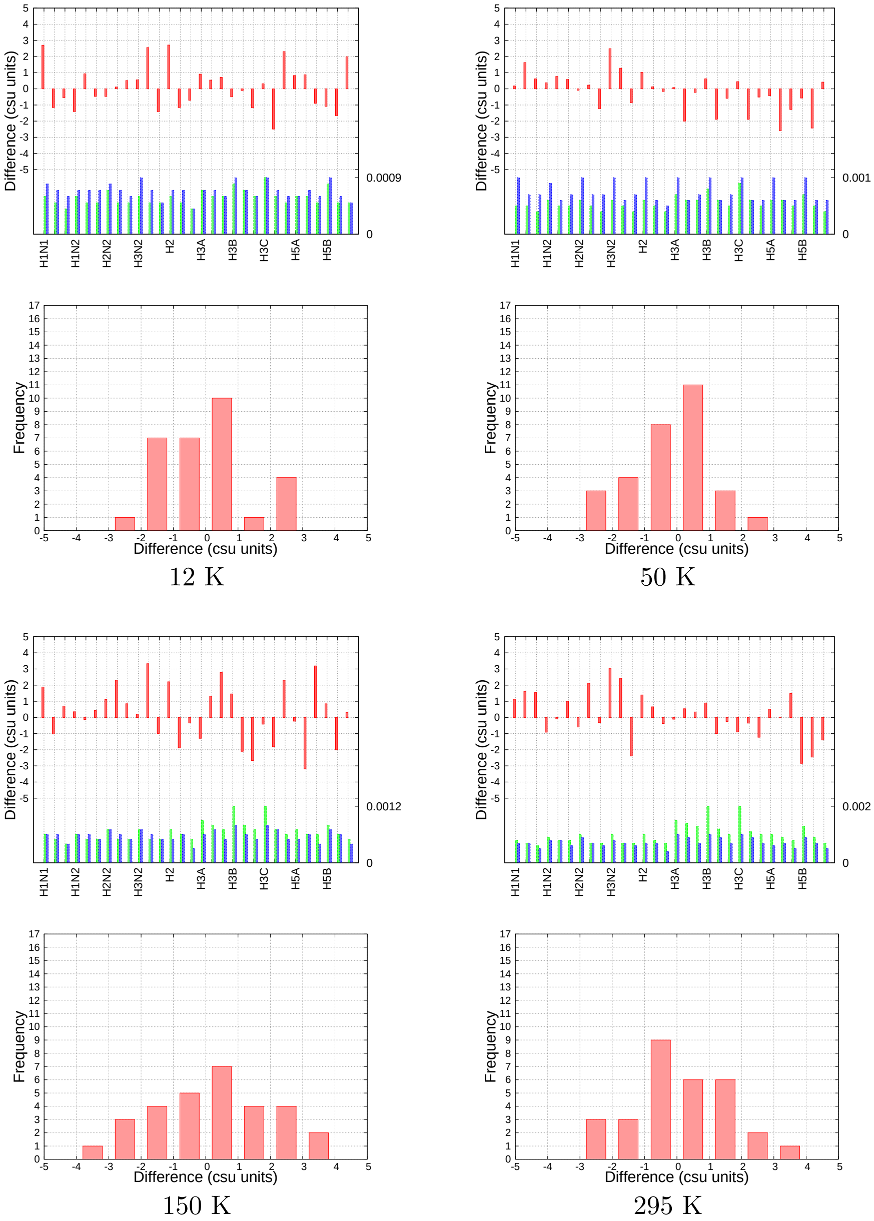

Figure S11: Hydrogen atom fractional coordinates, BLYP/cc-pVTZ

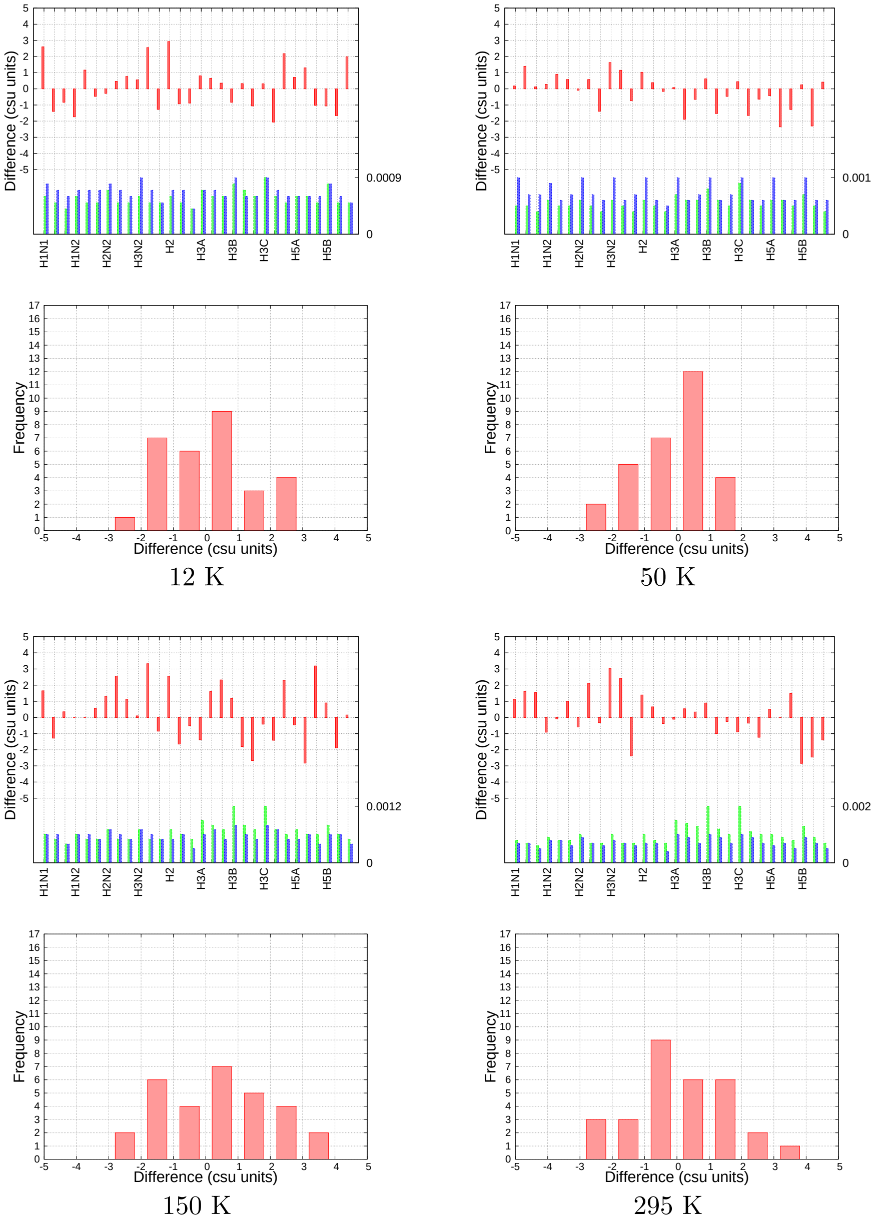

Figure S12: Hydrogen atom fractional coordinates, BLYP/cc-pVQZ

6 Statistical data for non-hydrogen atom bond lengths

Table S10: Mean absolute differences (MADs) of non-hydrogen atom bond lengths $\langle|\Delta d|\rangle$ and corresponding population standard deviations σ_{pop} in Å. No. of data averaged: 9.

T	Method	cc-pVDZ $\langle|\Delta d|\rangle$	σ_{pop}	cc-pVTZ $\langle|\Delta d|\rangle$	σ_{pop}	cc-pVQZ $\langle|\Delta d|\rangle$	σ_{pop}
12K	HF	0.001122	0.000836	0.001101	0.000790	0.001094	0.000775
	BLYP	0.001001	0.000734	0.000986	0.000781	0.001044	0.000737
50K	HF	0.002771	0.001686	0.003104	0.001702	0.003221	0.001702
	BLYP	0.002937	0.001702	0.003202	0.001884	0.003292	0.001931
150K	HF	0.002199	0.001177	0.002261	0.001246	0.002311	0.001291
	BLYP	0.002037	0.001033	0.002102	0.001104	0.002151	0.001156
295K	HF	0.002943	0.001879	0.003054	0.002230	0.003124	0.002273
	BLYP	0.003218	0.001748	0.003451	0.001988	0.003451	0.001988

Table S11: Mean differences (MDs) for non-hydrogen atom bond lengths $\langle\Delta d\rangle$ and corresponding population standard deviations σ_{pop} in Å. No. of data: averaged 9.

T	Method	cc-pVDZ $\langle\Delta d\rangle$	σ_{pop}	cc-pVTZ $\langle\Delta d\rangle$	σ_{pop}	cc-pVQZ $\langle\Delta d\rangle$	σ_{pop}
12K	HF	0.000746	0.001399	0.000870	0.001355	0.000878	0.001340
	BLYP	0.000666	0.001242	0.000809	0.001258	0.000809	0.001278
50K	HF	0.000235	0.003243	0.000330	0.003540	0.000338	0.003643
	BLYP	0.000249	0.003395	0.000381	0.003715	0.000381	0.003816
150K	HF	-0.000173	0.002494	-0.000019	0.002582	0.000009	0.002647
	BLYP	-0.000273	0.002284	-0.000101	0.002374	-0.000078	0.002442
295K	HF	-0.000370	0.003492	-0.000175	0.003782	-0.000119	0.003863
	BLYP	-0.000508	0.003662	-0.000303	0.003983	-0.000303	0.003983

Table S12: Csu-weighted root-mean-square differences (wRMSDs) of non-hydrogen atom bond lengths $\left\langle [\Delta d/\mathrm{csu}(d)]^2 \right\rangle^{1/2}$. No. of data: averaged 9.

T	Method	cc-pVDZ	cc-pVTZ	cc-pVQZ
12K	HF	0.557213	0.559553	0.561961
	BLYP	0.517736	0.549196	0.567727
50K	HF	1.400322	1.512199	1.552088
	BLYP	1.501773	1.634040	1.674012
150K	HF	0.927874	0.957337	0.975342
	BLYP	0.846509	0.886901	0.905364
295K	HF	0.858854	0.940217	0.964392
	BLYP	0.911703	1.014125	1.014128

Table S13: Mean ratios (MRs) of non-hydrogen atom bond lengths $\langle r \rangle$, $r = d(\text{X-ray})/d(\text{Neutron})$, and corresponding population standard deviations σ_{pop}. No. of data averaged: 9.

T	Method	cc-pVDZ		cc-pVTZ		cc-pVQZ	
		$\langle r \rangle$	σ_{pop}	$\langle r \rangle$	σ_{pop}	$\langle r \rangle$	σ_{pop}
12K	HF	1.000497	0.000830	1.000608	0.000715	1.000622	0.000694
	BLYP	1.000444	0.000750	1.000570	0.000674	1.000579	0.000690
50K	HF	1.000269	0.002291	1.000365	0.002515	1.000379	0.002596
	BLYP	1.000277	0.002391	1.000398	0.002625	1.000408	0.002706
150K	HF	0.999870	0.001766	1.000006	0.001800	1.000034	0.001845
	BLYP	0.999791	0.001634	0.999940	0.001660	0.999965	0.001704
295K	HF	0.999789	0.002398	0.999964	0.002605	1.000013	0.002661
	BLYP	0.999667	0.002527	0.999842	0.002766	0.999842	0.002766

7 Statistical plots for non-hydrogen atom bond lengths

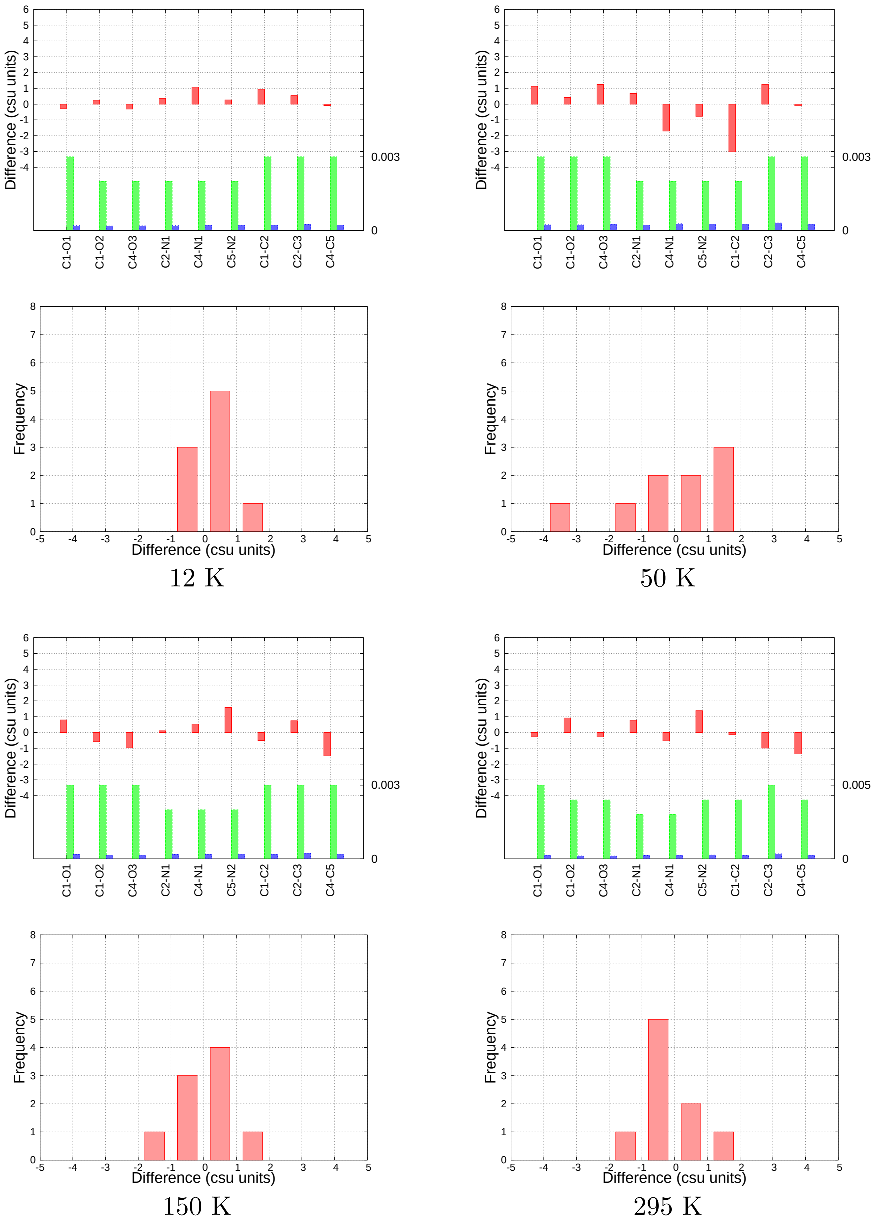

Figure S13: Non-hydrogen atom bond lengths in Å, HF/cc-pVDZ

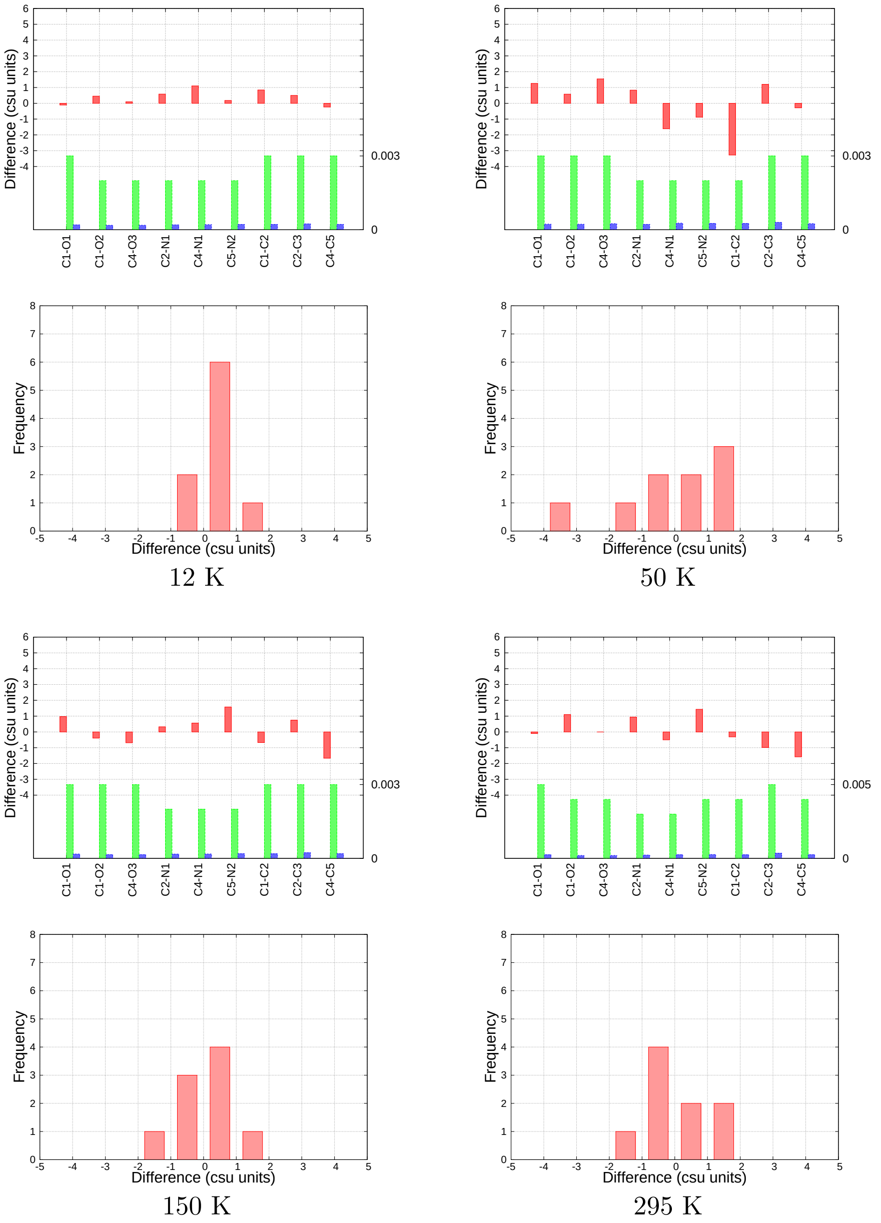

Figure S14: Non-hydrogen atom bond lengths in Å, HF/cc-pVTZ

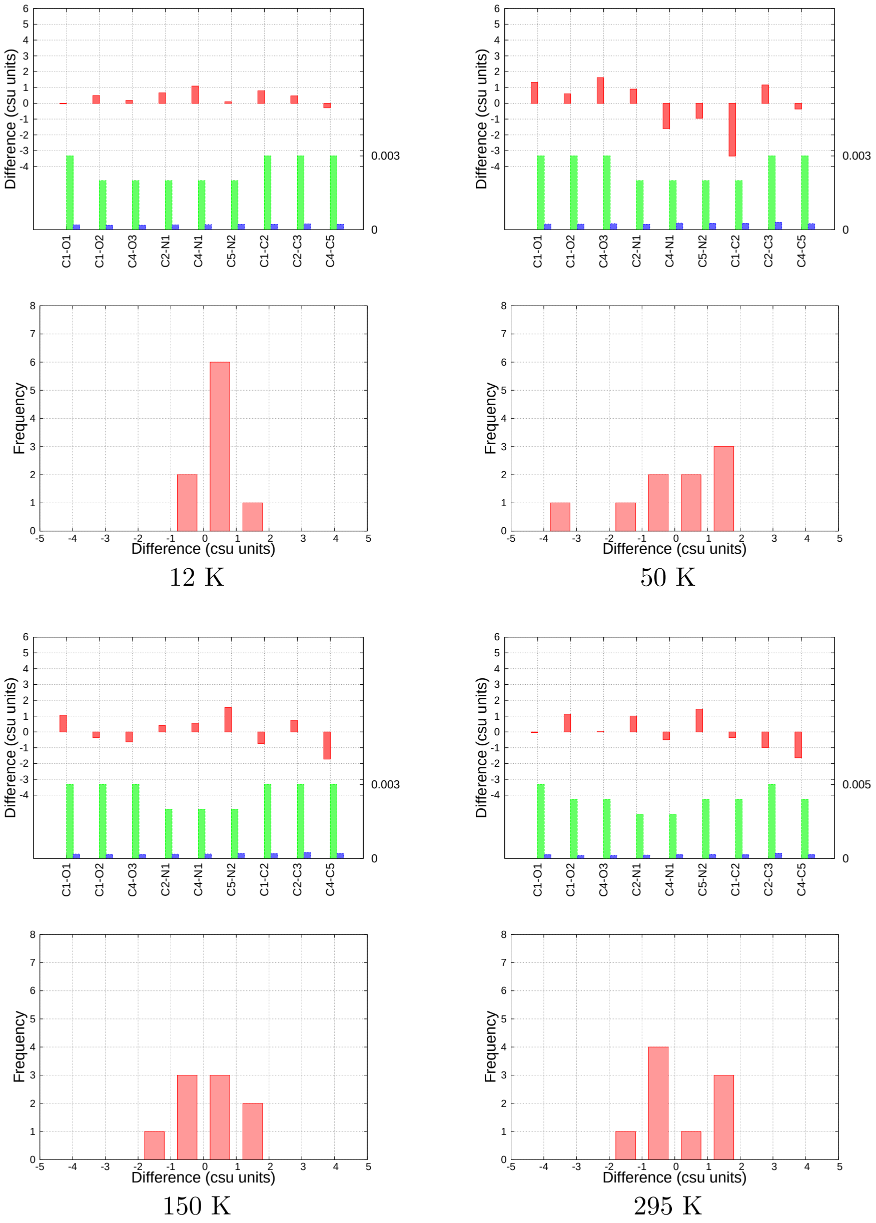

Figure S15: Non-hydrogen atom bond lengths in Å, HF/cc-pVQZ

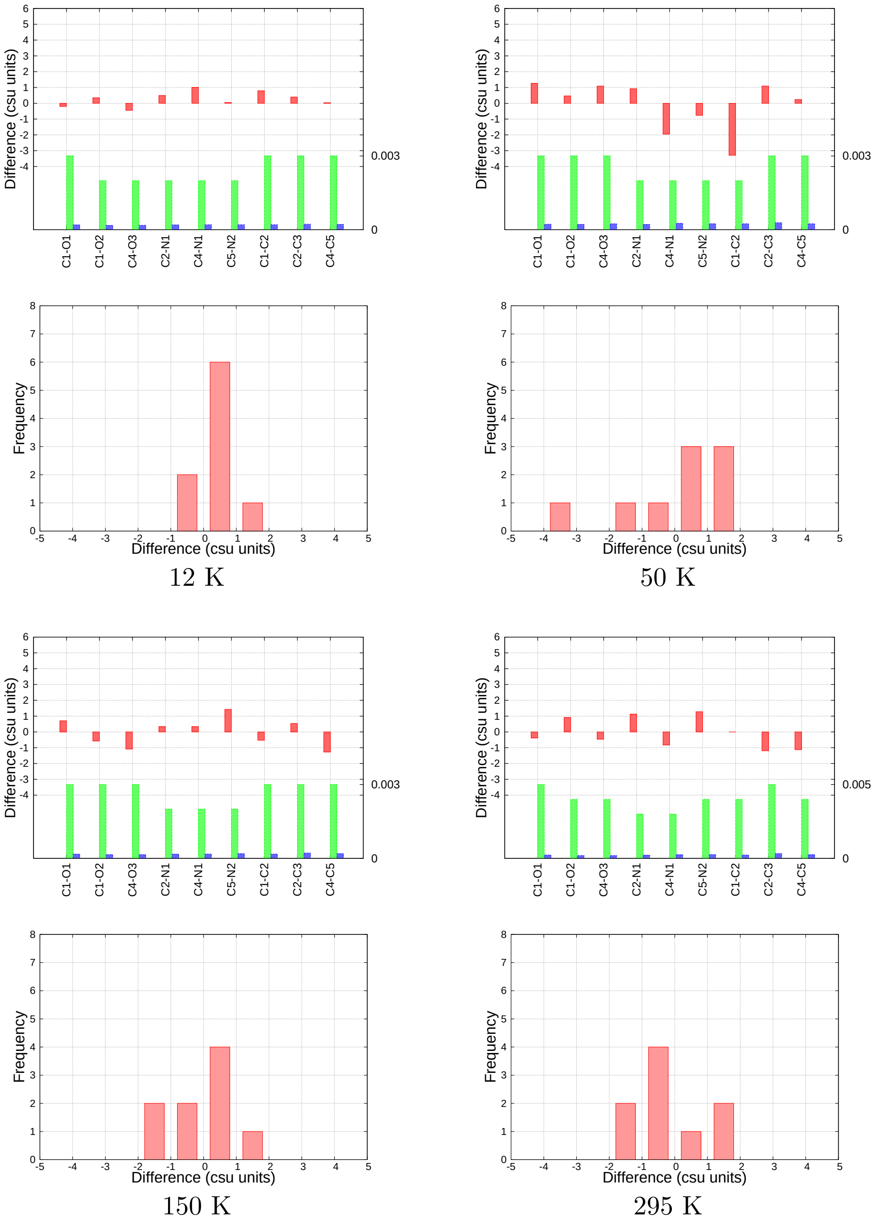

Figure S16: Non-hydrogen atom bond lengths in Å, BLYP/cc-pVDZ

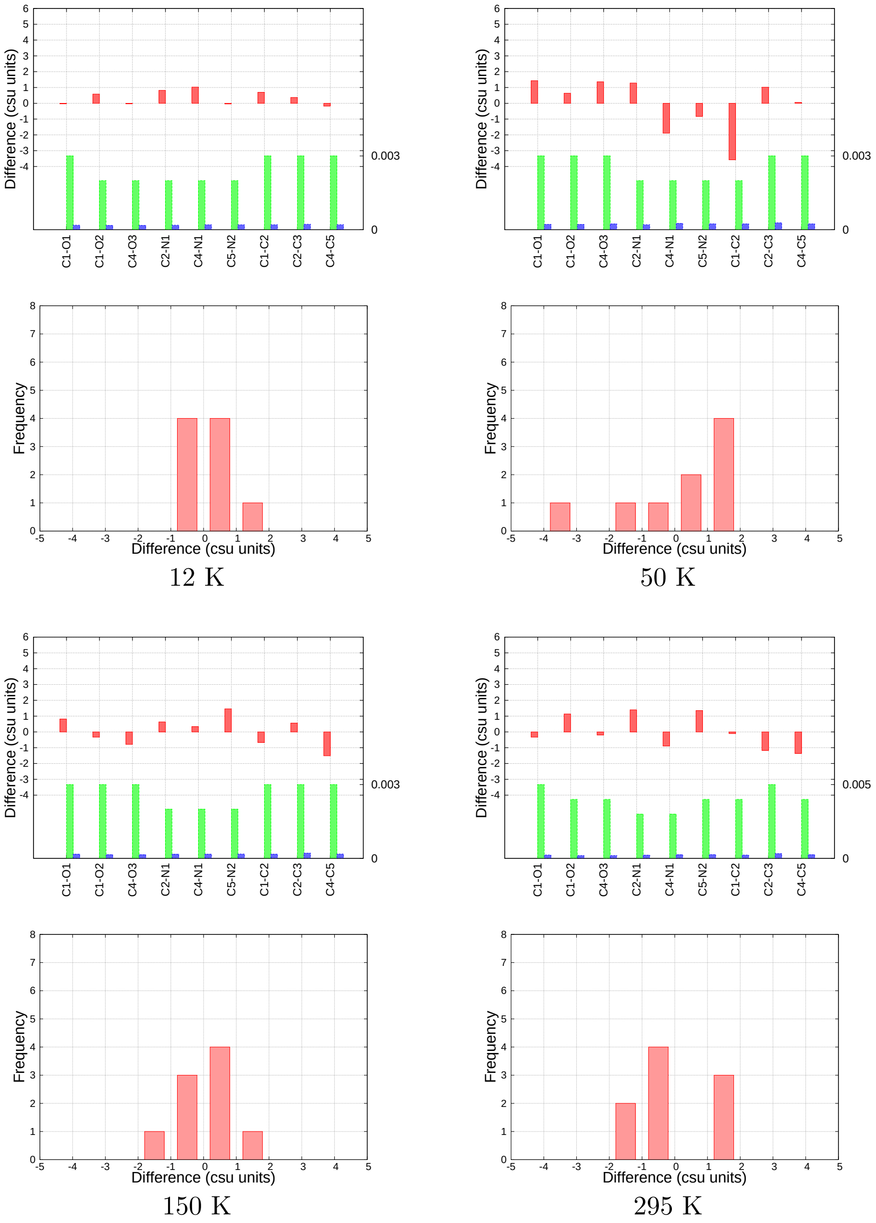

Figure S17: Non-hydrogen atom bond lengths in Å, BLYP/cc-pVTZ

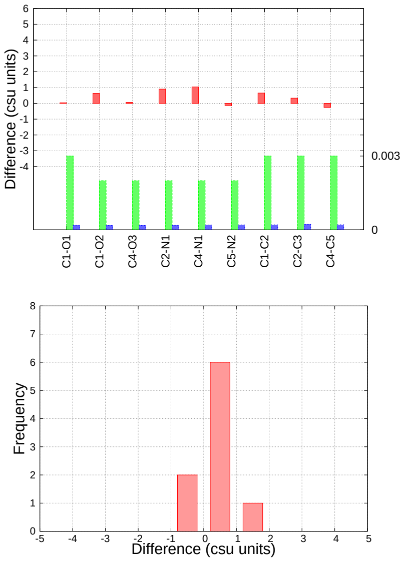

12 K

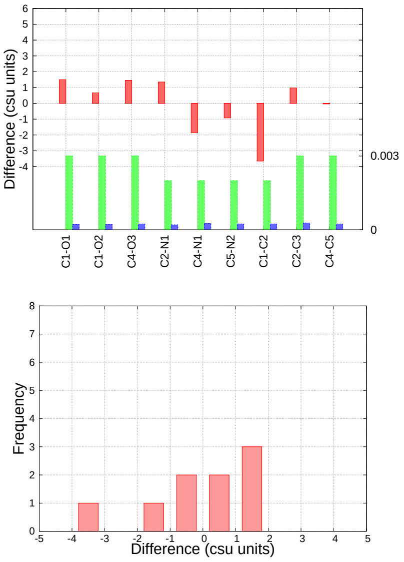

50 K

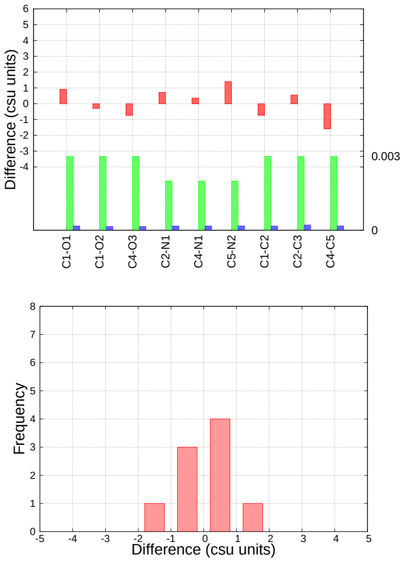

150 K

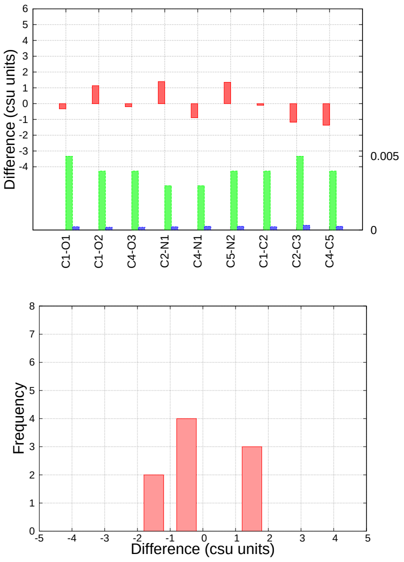

295 K

Figure S18: Non-hydrogen atom bond lengths in Å, BLYP/cc-pVQZ

8 Statistical data for D-H bond lengths

Table S14: Mean absolute differences (MADs) of D-H bond lengths $\langle|\Delta d|\rangle$ and corresponding population standard deviations σ_{pop} in Å. No. of data averaged: 10.

T	Method	cc-pVDZ $\langle|\Delta d|\rangle$	σ_{pop}	cc-pVTZ $\langle|\Delta d|\rangle$	σ_{pop}	cc-pVQZ $\langle|\Delta d|\rangle$	σ_{pop}
12K	HF	0.006759	0.006216	0.008418	0.004518	0.008249	0.004284
	BLYP	0.008205	0.004757	0.008539	0.005132	0.008882	0.004871
50K	HF	0.013442	0.009976	0.014082	0.010326	0.014446	0.009465
	BLYP	0.009173	0.002750	0.009252	0.005225	0.008924	0.005400
150K	HF	0.010017	0.006411	0.012666	0.006950	0.013466	0.006484
	BLYP	0.008146	0.002867	0.008670	0.006168	0.009102	0.006129
295K	HF	0.008798	0.003785	0.008863	0.004913	0.009037	0.005833
	BLYP	0.008827	0.008659	0.012457	0.008435	0.012458	0.008435

Table S15: Mean differences (MDs) for D-H bond lengths $\langle\Delta d\rangle$ and corresponding population standard deviations σ_{pop} in Å. No. of data: averaged 10.

T	Method	cc-pVDZ $\langle\Delta d\rangle$	σ_{pop}	cc-pVTZ $\langle\Delta d\rangle$	σ_{pop}	cc-pVQZ $\langle\Delta d\rangle$	σ_{pop}
12K	HF	0.005031	0.009183	0.008148	0.009554	0.008223	0.009295
	BLYP	-0.007198	0.009485	-0.006019	0.009963	-0.005862	0.010130
50K	HF	0.010453	0.016739	0.014082	0.017462	0.014446	0.017271
	BLYP	-0.001890	0.009577	-0.000188	0.010626	0.000434	0.010431
150K	HF	0.008673	0.011893	0.011659	0.014448	0.011924	0.014946
	BLYP	-0.003319	0.008636	-0.002521	0.010640	-0.002057	0.010973
295K	HF	0.001865	0.009578	0.003918	0.010133	0.004194	0.010756
	BLYP	-0.008662	0.012365	-0.008935	0.015044	-0.008934	0.015045

Table S16: Csu-weighted root-mean-square differences (wRMSDs) of D-H bond lengths $\langle [\Delta d/\text{csu}(d)]^2 \rangle^{1/2}$. No. of data: averaged 10.

T	Method	cc-pVDZ	cc-pVTZ	cc-pVQZ
12K	HF	1.676964	1.712758	1.659692
	BLYP	1.661077	1.775959	1.801854
50K	HF	2.861061	2.985122	2.957359
	BLYP	1.638244	1.802856	1.754762
150K	HF	2.040617	2.406803	2.480419
	BLYP	1.484003	1.791486	1.826654
295K	HF	1.154971	1.251866	1.317044
	BLYP	1.582417	1.906538	1.906633

Table S17: Mean ratios (MRs) of D-H bond lengths $\langle r \rangle$, $r = d(\text{X-ray})/d(\text{Neutron})$, and corresponding population standard deviations σ_{pop}. No. of data averaged: 10.

T	Method	cc-pVDZ		cc-pVTZ		cc-pVQZ	
		$\langle r \rangle$	σ_{pop}	$\langle r \rangle$	σ_{pop}	$\langle r \rangle$	σ_{pop}
12K	HF	1.004822	0.007305	1.007663	0.004758	1.007714	0.004112
	BLYP	0.993332	0.005771	0.994337	0.007383	0.994475	0.007659
50K	HF	1.009927	0.012318	1.013277	0.009789	1.013609	0.008985
	BLYP	0.998286	0.008745	0.999825	0.009811	1.000410	0.009604
150K	HF	1.008191	0.007672	1.010927	0.008015	1.011159	0.008456
	BLYP	0.996881	0.007491	0.997547	0.009716	0.997969	0.010118
295K	HF	1.001854	0.008896	1.003724	0.008876	1.003964	0.009389
	BLYP	0.991851	0.008371	0.991507	0.011407	0.991507	0.011408

9 Statistical plots for D-H bond lengths

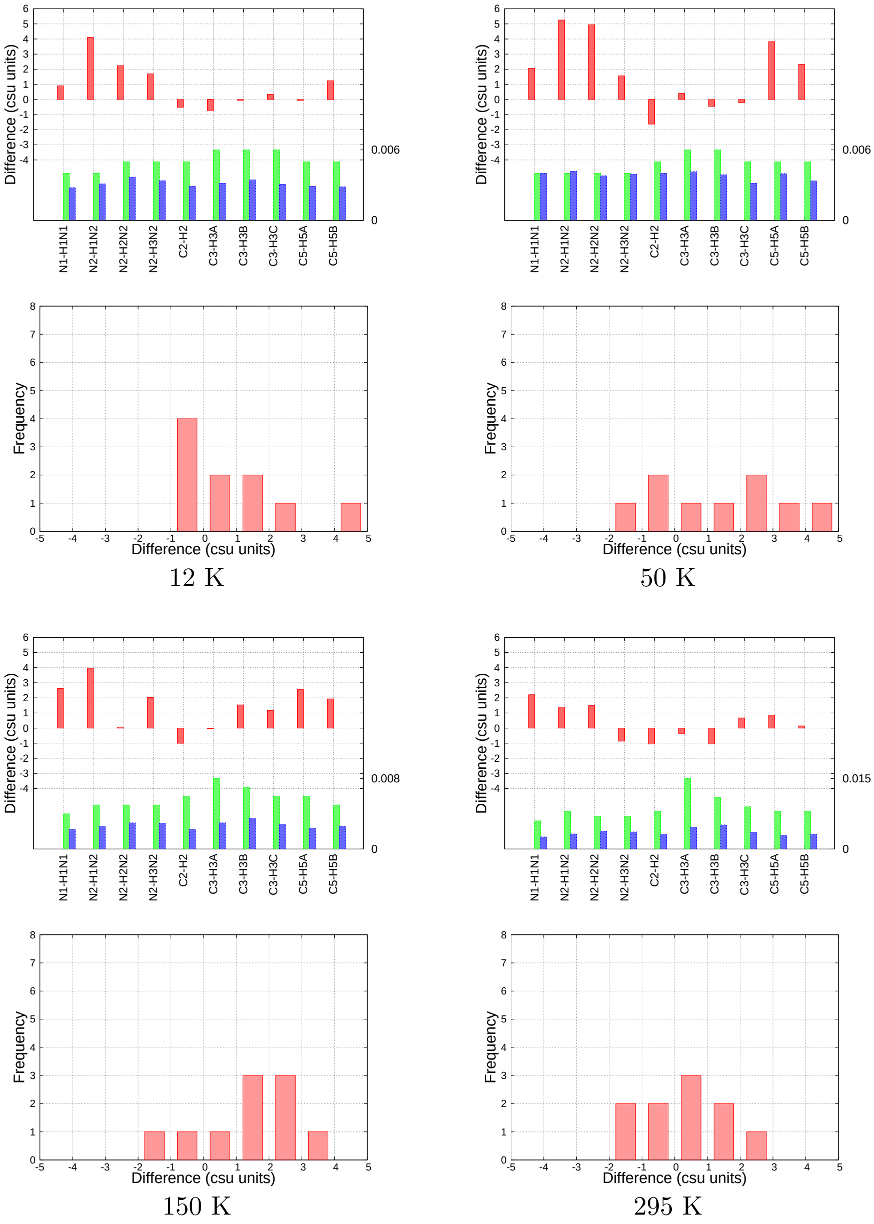

Figure S19: D-H bond lengths in Å, HF/cc-pVDZ

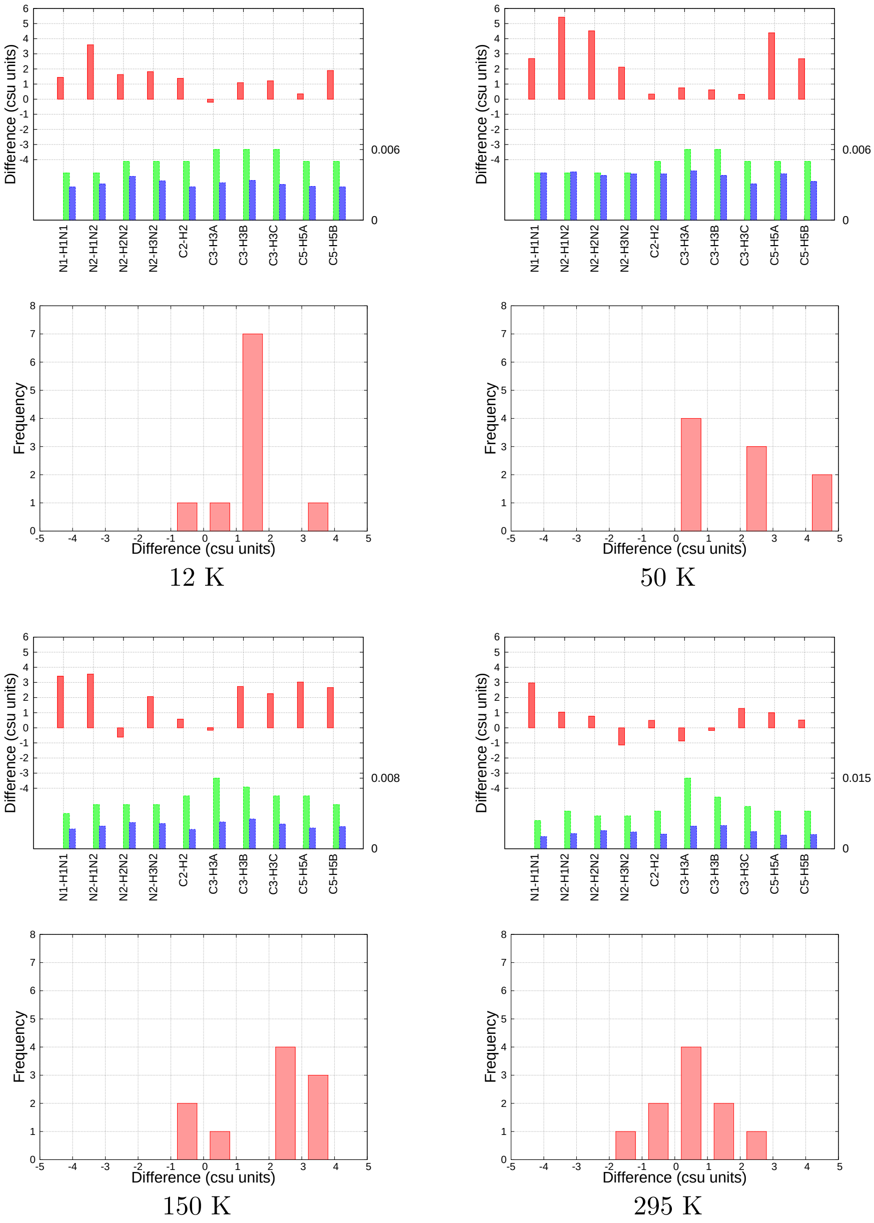

Figure S20: D-H bond lengths in Å, HF/cc-pVTZ

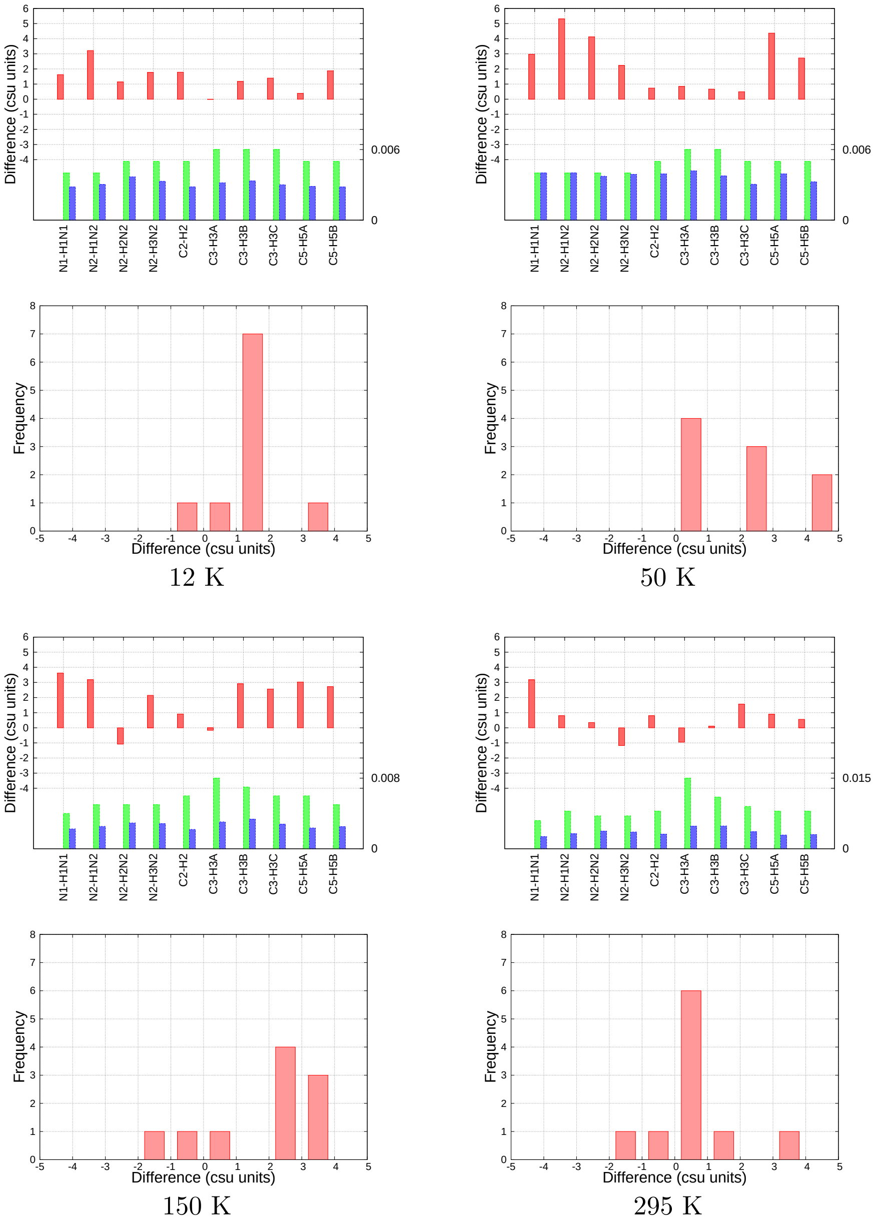

Figure S21: D-H bond lengths in Å, HF/cc-pVQZ

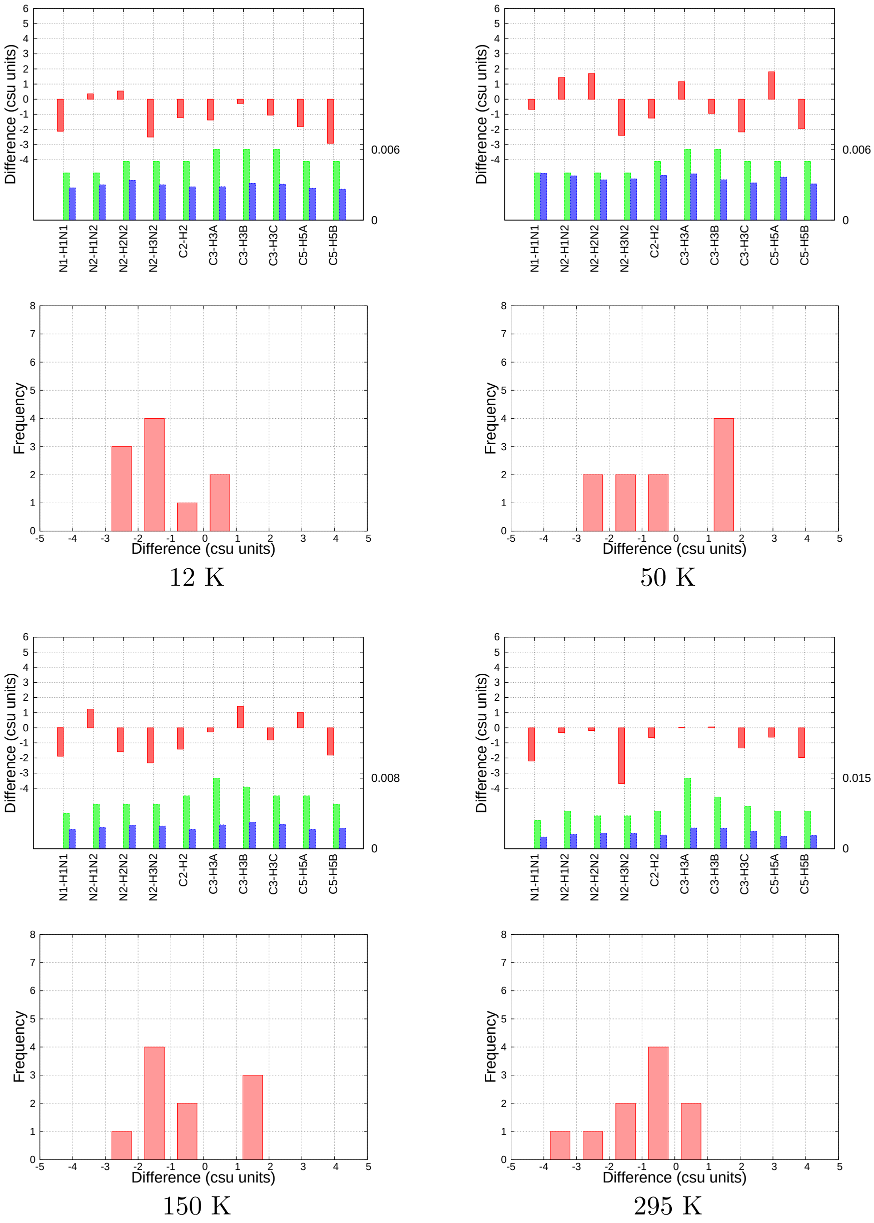

Figure S22: D-H bond lengths in Å, BLYP/cc-pVDZ

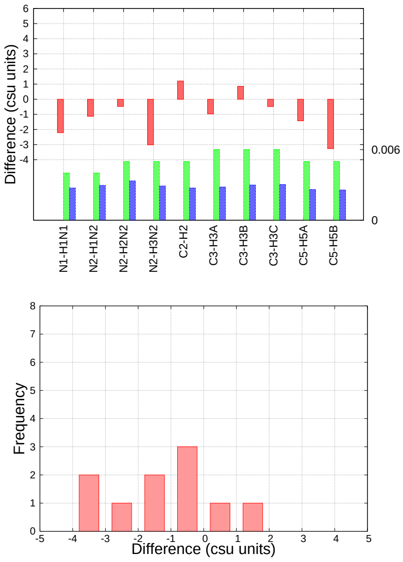

12 K

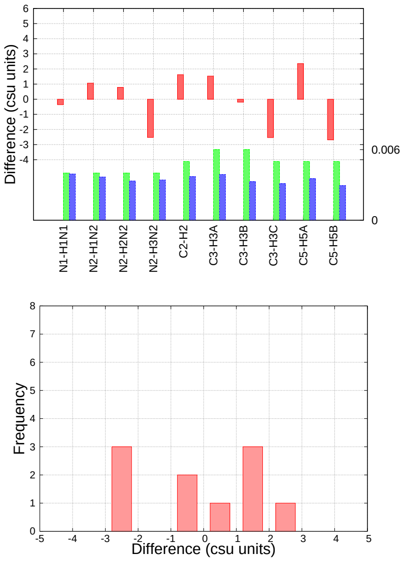

50 K

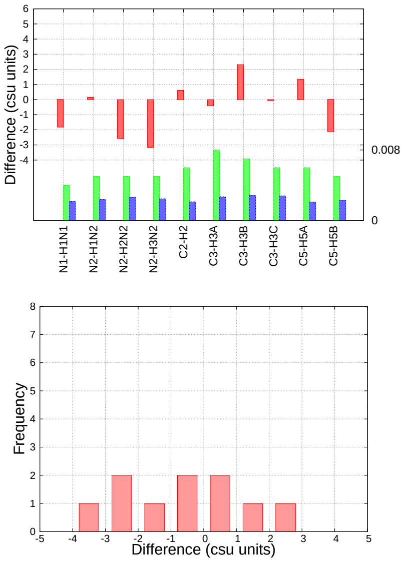

150 K

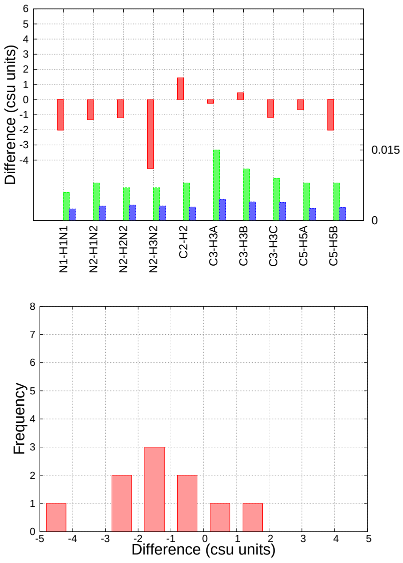

295 K

Figure S23: D-H bond lengths in Å, BLYP/cc-pVTZ

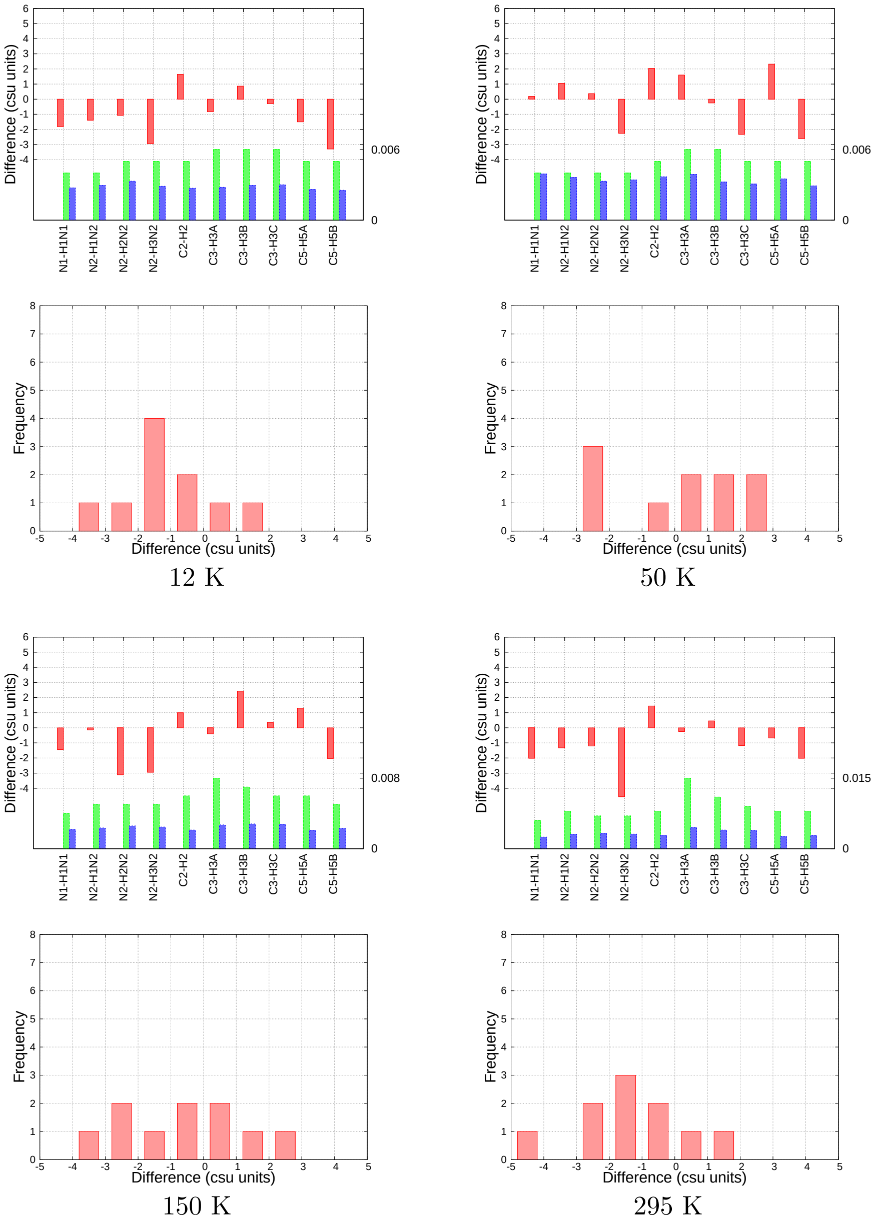

Figure S24: D-H bond lengths in Å, BLYP/cc-pVQZ

10 Statistical data for non-hydrogen atom ADPs

Table S18: Mean absolute differences (MADs) of non-hydrogen atom ADPs $\langle|\Delta U_{ij}|\rangle$ and corresponding population standard deviations σ_{pop} in Å^2. No. of data averaged: 60.

T	Method	cc-pVDZ $\langle\lvert\Delta U_{ij}\rvert\rangle$	σ_{pop}	cc-pVTZ $\langle\lvert\Delta U_{ij}\rvert\rangle$	σ_{pop}	cc-pVQZ $\langle\lvert\Delta U_{ij}\rvert\rangle$	σ_{pop}
12K	HF	0.001090	0.001089	0.001056	0.001045	0.001059	0.001040
	BLYP	0.001236	0.001189	0.001153	0.001136	0.001141	0.001123
50K	HF	0.001203	0.001242	0.001152	0.001213	0.001152	0.001219
	BLYP	0.001290	0.001334	0.001194	0.001290	0.001180	0.001286
150K	HF	0.001182	0.001216	0.001137	0.001163	0.001130	0.001156
	BLYP	0.001315	0.001270	0.001249	0.001194	0.001233	0.001177
295K	HF	0.001319	0.001109	0.001336	0.001072	0.001333	0.001066
	BLYP	0.001373	0.001114	0.001357	0.001008	0.001357	0.001008

Table S19: Mean differences (MDs) for non-hydrogen atom ADPs $\langle\Delta U\rangle$ and corresponding population standard deviations σ_{pop} in Å^2. No. of data: averaged 60.

T	Method	cc-pVDZ $\langle\Delta U\rangle$	σ_{pop}	cc-pVTZ $\langle\Delta U\rangle$	σ_{pop}	cc-pVQZ $\langle\Delta U\rangle$	σ_{pop}
12K	HF	-0.000823	0.001541	-0.000815	0.001485	-0.000818	0.001484
	BLYP	-0.000951	0.001715	-0.000905	0.001619	-0.000898	0.001601
50K	HF	-0.000893	0.001729	-0.000880	0.001673	-0.000884	0.001677
	BLYP	-0.001001	0.001856	-0.000951	0.001758	-0.000940	0.001745
150K	HF	-0.000787	0.001696	-0.000768	0.001627	-0.000777	0.001617
	BLYP	-0.000905	0.001828	-0.000853	0.001727	-0.000842	0.001705
295K	HF	-0.000802	0.001724	-0.000784	0.001713	-0.000786	0.001707
	BLYP	-0.000874	0.001769	-0.000759	0.001691	-0.000759	0.001691

Table S20: Csu-weighted root-mean-square differences (wRMSDs) of non-hydrogen atom ADPs $\left\langle [\Delta U_{ij}/\mathrm{csu}(U_{ij})]^2 \right\rangle^{1/2}$. No. of data: averaged 60.

T	Method	cc-pVDZ	cc-pVTZ	cc-pVQZ
12K	HF	2.624725	2.525565	2.526459
	BLYP	2.897461	2.734121	2.703618
50K	HF	2.666691	2.589051	2.594691
	BLYP	2.836473	2.686887	2.669673
150K	HF	2.308936	2.211385	1.930859
	BLYP	2.469452	2.326422	2.300628
295K	HF	1.486751	1.486372	1.480162
	BLYP	1.481299	1.415492	1.415492

Table S21: Mean ratios (MRs) of non-hydrogen atom ADPs $\langle r \rangle$ for diagonal elements, $r = U_{ii}(\text{X-ray})/U_{ii}(\text{Neutron})$, and corresponding population standard deviations σ_{pop}. No. of data averaged: 30.

T	Method	cc-pVDZ		cc-pVTZ		cc-pVQZ	
		$\langle r \rangle$	σ_{pop}	$\langle r \rangle$	σ_{pop}	$\langle r \rangle$	σ_{pop}
12K	HF	0.740119	0.170907	0.750579	0.166088	0.751647	0.167306
	BLYP	0.692667	0.184053	0.711751	0.177196	0.715515	0.175831
50K	HF	0.779114	0.166116	0.788322	0.163865	0.789615	0.165321
	BLYP	0.743533	0.167385	0.763786	0.164968	0.768415	0.166734
150K	HF	0.889324	0.094512	0.895412	0.092325	0.895537	0.091667
	BLYP	0.869333	0.097352	0.879288	0.095122	0.881585	0.094243
295K	HF	0.962327	0.062765	0.966640	0.062434	0.967380	0.061648
	BLYP	0.956159	0.066207	0.967150	0.065682	0.967150	0.065682

11 Statistical plots for non-hydrogen atom ADPs

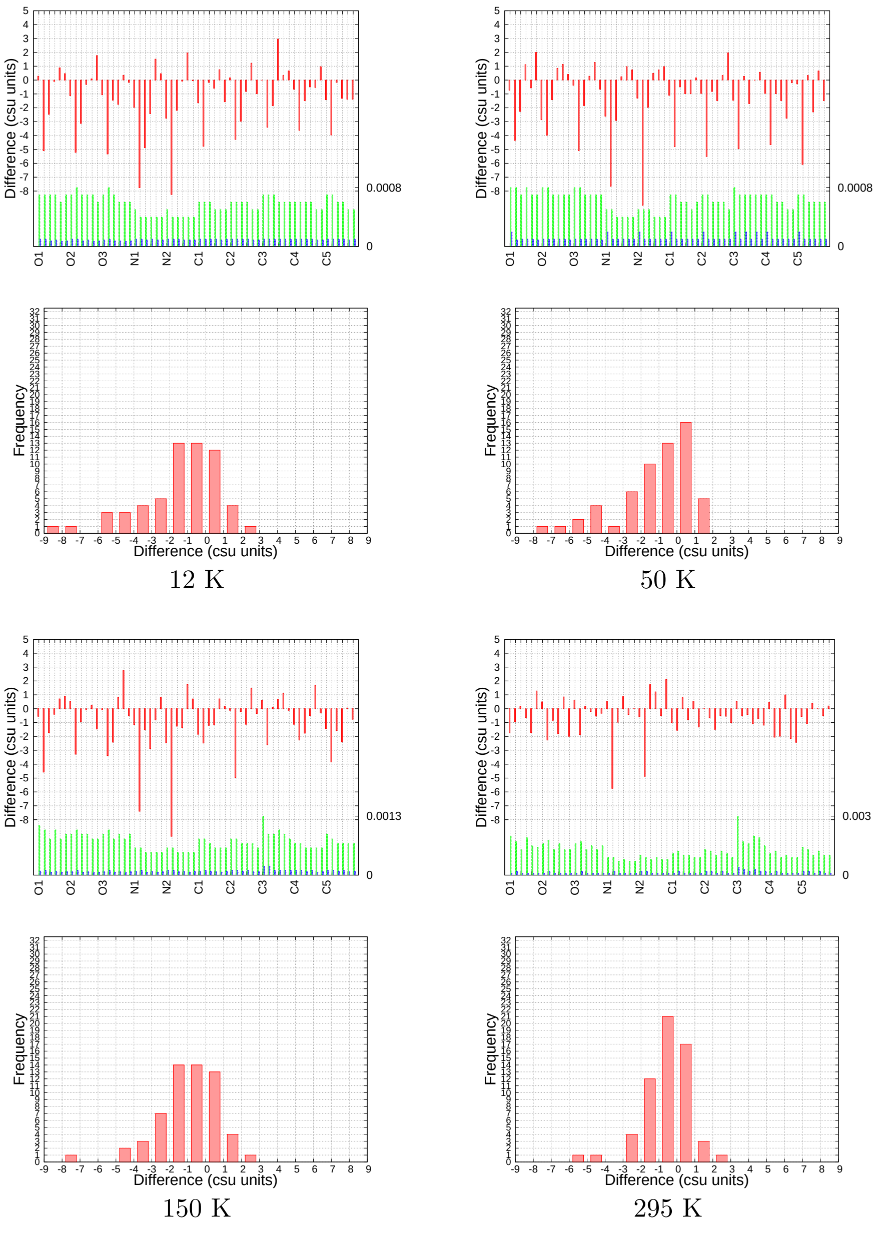

Figure S25: Non-hydrogen atom ADPs in Å^2, HF/cc-pVDZ

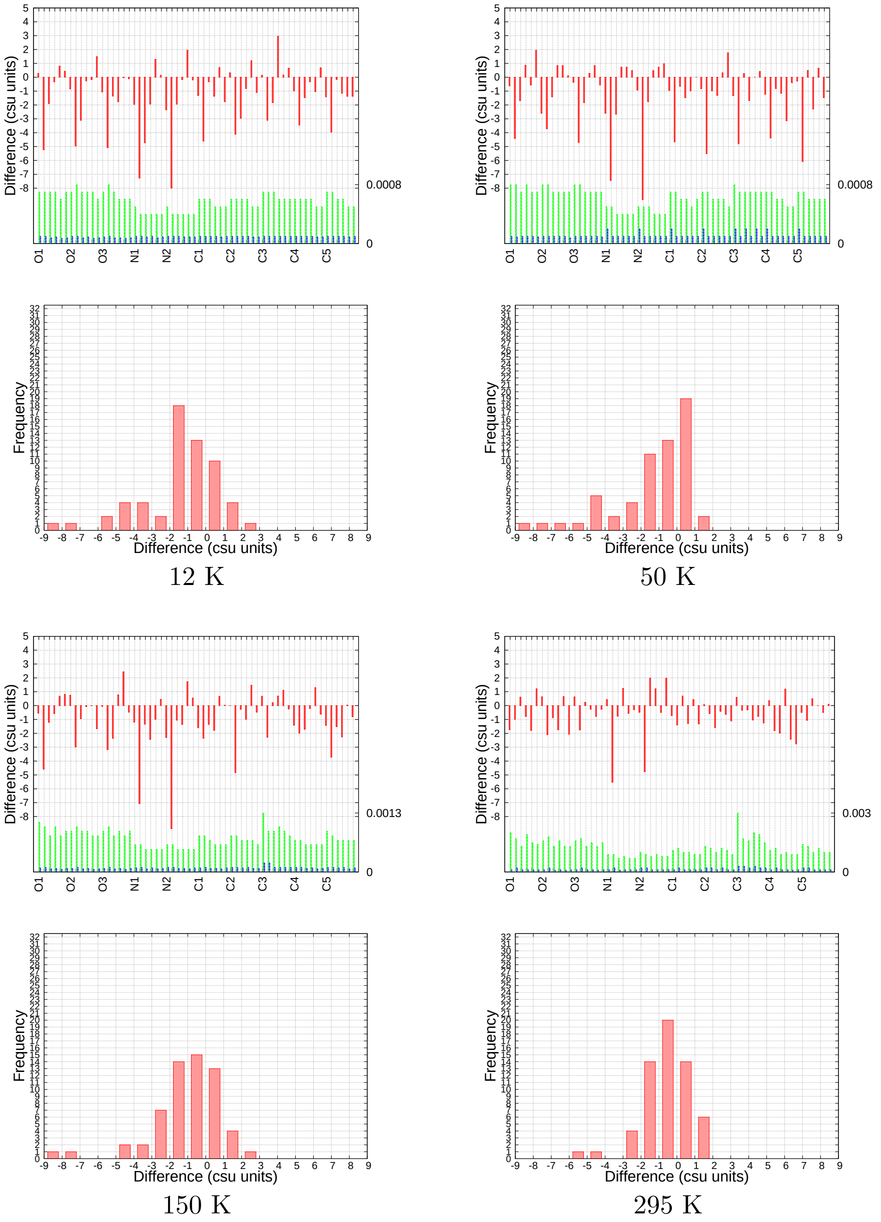

Figure S26: Non-hydrogen atom ADPs in Å^2, HF/cc-pVTZ

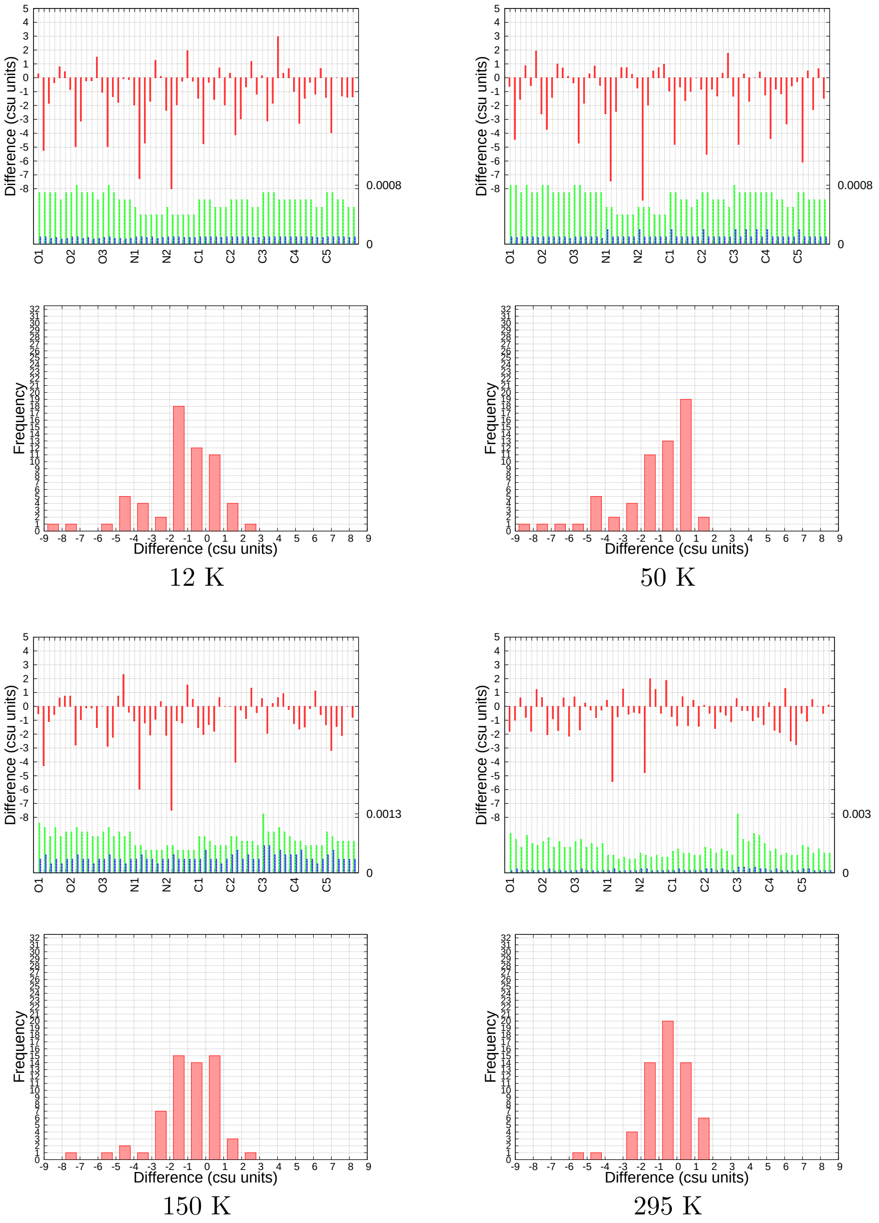

Figure S27: Non-hydrogen atom ADPs in Å^2, HF/cc-pVQZ

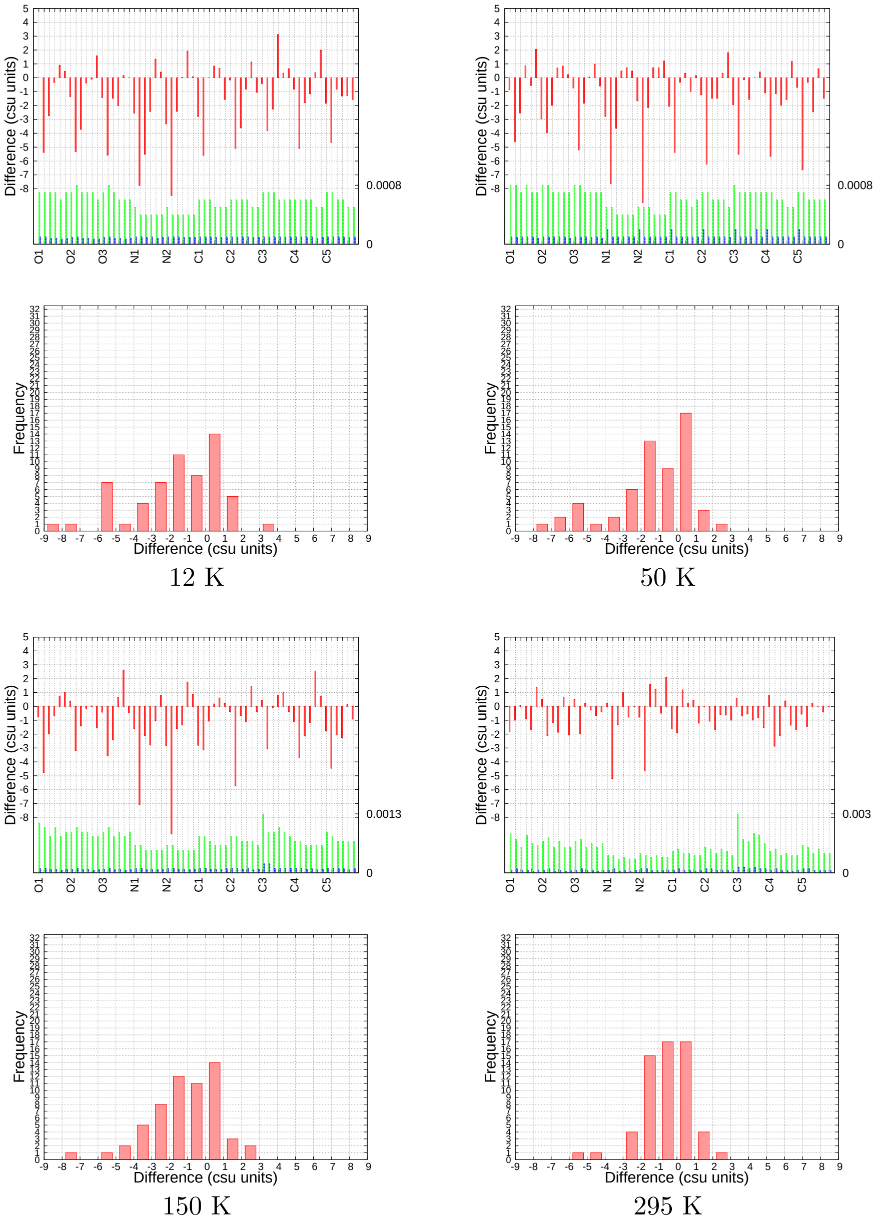

Figure S28: Non-hydrogen atom ADPs in Å^2, BLYP/cc-pVDZ

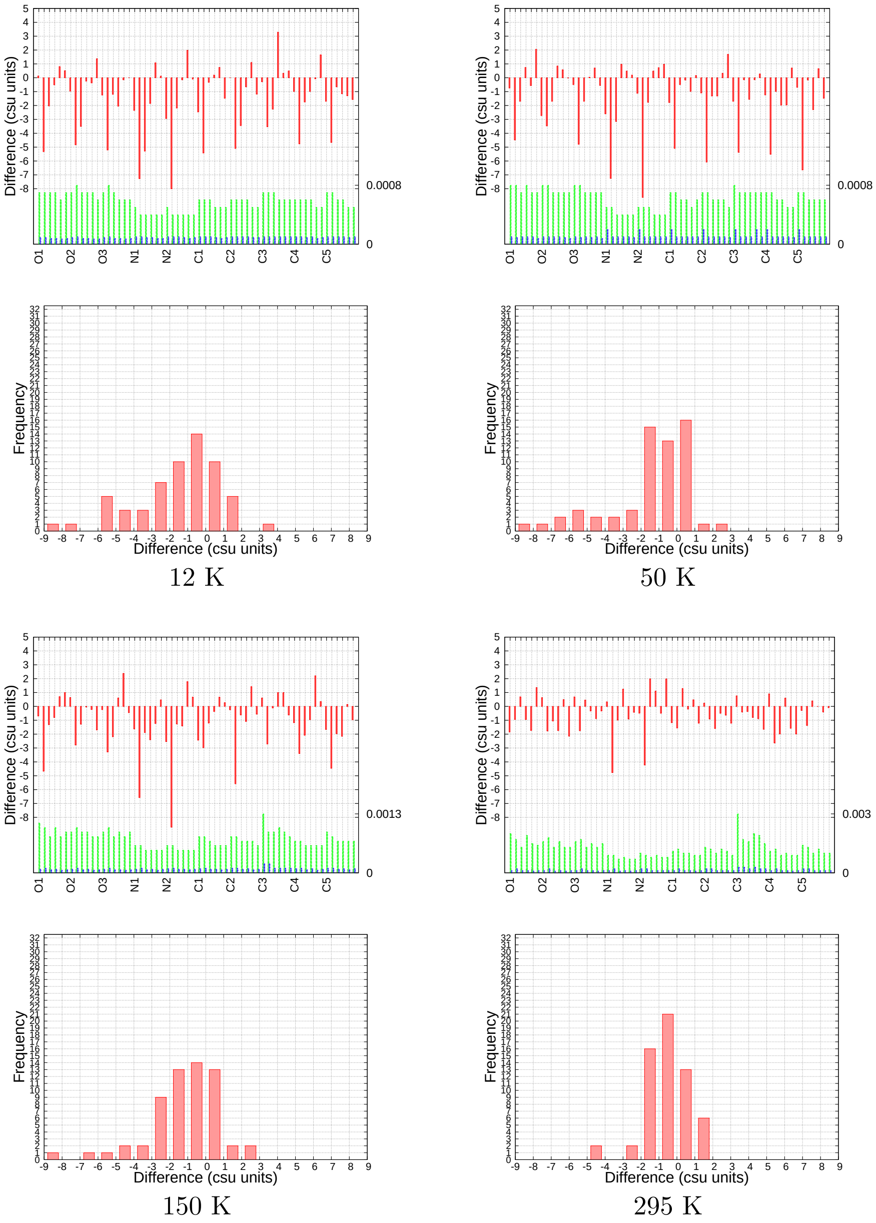

Figure S29: Non-hydrogen atom ADPs in Å^2, BLYP/cc-pVTZ

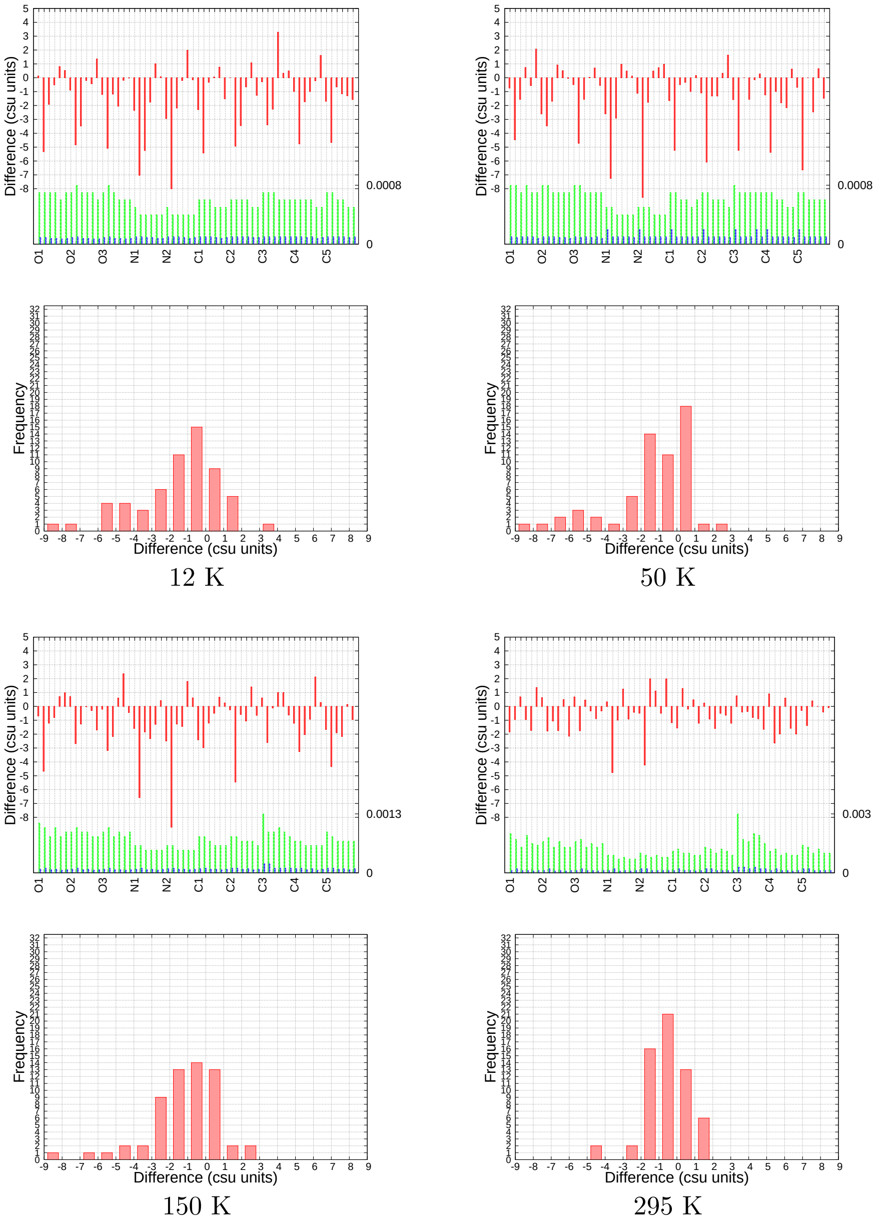

Figure S30: Non-hydrogen atom ADPs in $Å^2$, BLYP/cc-pVQZ

12 Statistical data for hydrogen atom ADPs

Table S22: Mean absolute differences (MADs) of hydrogen atom ADPs $\langle|\Delta U_{ij}|\rangle$ and corresponding population standard deviations σ_{pop} in Å^2. No. of data averaged: 60.

T	Method	cc-pVDZ $\langle\|\Delta U_{ij}\|\rangle$	σ_{pop}	cc-pVTZ $\langle\|\Delta U_{ij}\|\rangle$	σ_{pop}	cc-pVQZ $\langle\|\Delta U_{ij}\|\rangle$	σ_{pop}
12K	HF	0.005873	0.004076	0.004993	0.003702	0.004977	0.003724
	BLYP	0.005393	0.004367	0.004653	0.004217	0.004687	0.004224
50K	HF	0.006177	0.004593	0.005000	0.004246	0.004967	0.004046
	BLYP	0.005297	0.004034	0.004003	0.003443	0.003970	0.003338
150K	HF	0.005447	0.004497	0.004850	0.003663	0.005327	0.004469
	BLYP	0.004453	0.004008	0.003663	0.003133	0.003647	0.003122
295K	HF	0.010400	0.008804	0.009683	0.007758	0.009367	0.007559
	BLYP	0.008000	0.006385	0.007450	0.005795	0.007450	0.005795

Table S23: Mean differences (MDs) for hydrogen atom ADPs $\langle\Delta U\rangle$ and corresponding population standard deviations σ_{pop} in Å^2. No. of data: averaged 60.

T	Method	cc-pVDZ $\langle\Delta U\rangle$	σ_{pop}	cc-pVTZ $\langle\Delta U\rangle$	σ_{pop}	cc-pVQZ $\langle\Delta U\rangle$	σ_{pop}
12K	HF	-0.001350	0.007149	-0.000050	0.006216	-0.000150	0.006216
	BLYP	-0.002200	0.006940	-0.000267	0.006280	-0.000350	0.006309
50K	HF	-0.001280	0.007697	0.000003	0.006560	-0.000030	0.006406
	BLYP	-0.002630	0.006658	-0.000680	0.005281	-0.000813	0.005187
150K	HF	-0.001430	0.007063	0.000003	0.006078	-0.000147	0.006953
	BLYP	-0.002613	0.005991	-0.000480	0.004820	-0.000497	0.004800
295K	HF	0.001033	0.013626	0.002383	0.012408	0.002167	0.012036
	BLYP	-0.001000	0.010236	0.001283	0.009438	0.001283	0.009438

Table S24: Csu-weighted root-mean-square differences (wRMSDs) of hydrogen atom ADPs $\left\langle [\Delta U_{ij}/\mathrm{csu}(U_{ij})]^2 \right\rangle^{1/2}$. No. of data: averaged 60.

T	Method	cc-pVDZ	cc-pVTZ	cc-pVQZ
12K	HF	1.945640	1.712070	1.706289
	BLYP	1.999283	1.830323	1.841836
50K	HF	1.845716	1.493656	1.484204
	BLYP	1.717691	1.292494	1.257232
150K	HF	1.734508	1.525596	0.685231
	BLYP	1.557473	1.426491	1.439919
295K	HF	1.966212	1.780602	1.725434
	BLYP	1.656165	1.607518	1.607518

Table S25: Mean ratios (MRs) of hydrogen atom ADPs $\langle r\rangle$ for diagonal elements, $r = U_{ii}(\text{X-ray})/U_{ii}(\text{Neutron})$, and corresponding population standard deviations σ_{pop}. No. of data averaged: 30.

T	Method	cc-pVDZ		cc-pVTZ		cc-pVQZ	
		$\langle r\rangle$	σ_{pop}	$\langle r\rangle$	σ_{pop}	$\langle r\rangle$	σ_{pop}
12K	HF	0.892092	0.386815	1.013665	0.334025	1.003950	0.339050
	BLYP	0.798897	0.312733	0.971171	0.318888	0.964122	0.317709
50K	HF	0.868458	0.364408	0.988599	0.326270	0.980772	0.324584
	BLYP	0.772995	0.311272	0.941492	0.313424	0.934082	0.310385
150K	HF	0.927826	0.203969	1.013072	0.180484	1.014254	0.214481
	BLYP	0.866438	0.135265	0.989109	0.145564	0.979957	0.142395
295K	HF	1.017317	0.240993	1.078869	0.214357	1.074480	0.212488
	BLYP	0.961265	0.172638	1.048397	0.148622	1.048397	0.148622

13 Statistical plots for hydrogen atom ADPs

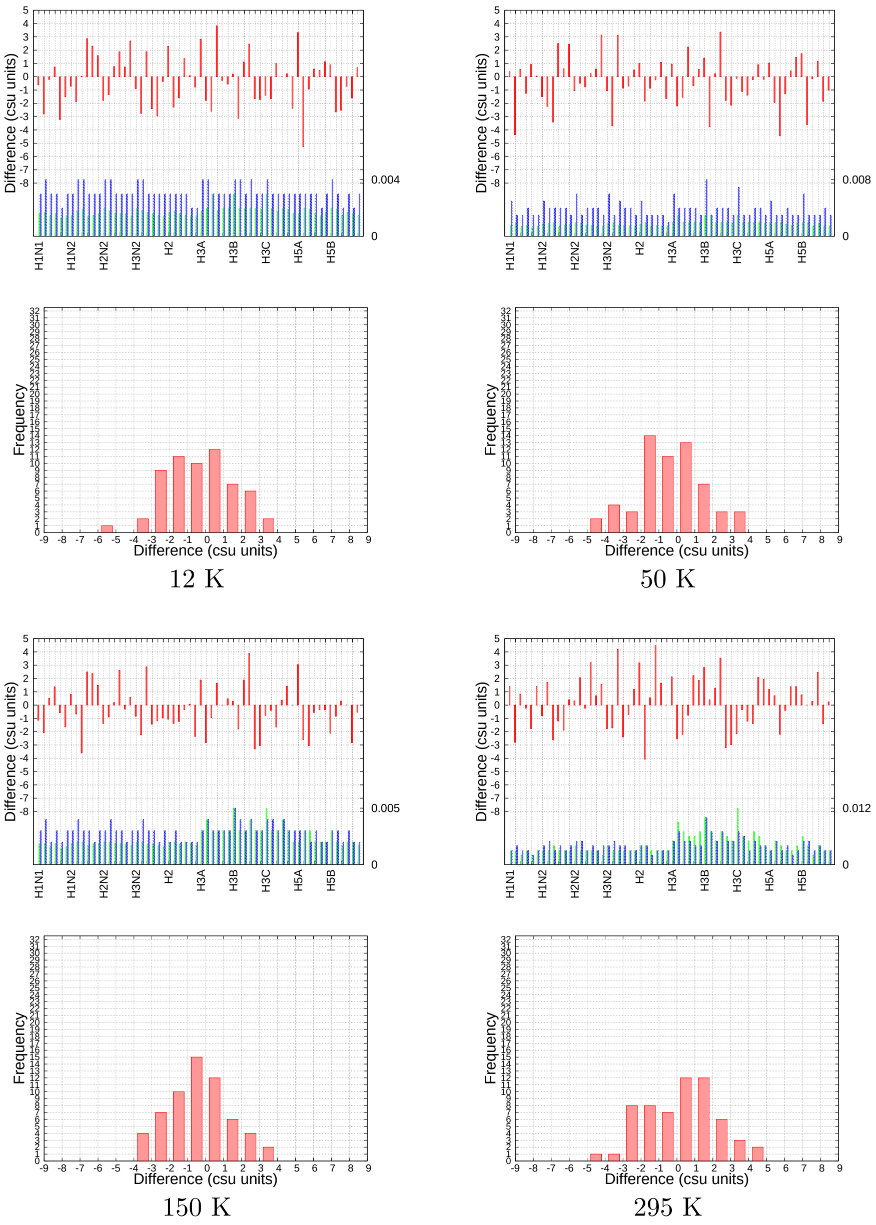

Figure S31: Hydrogen atom ADPs in Å^2, HF/cc-pVDZ

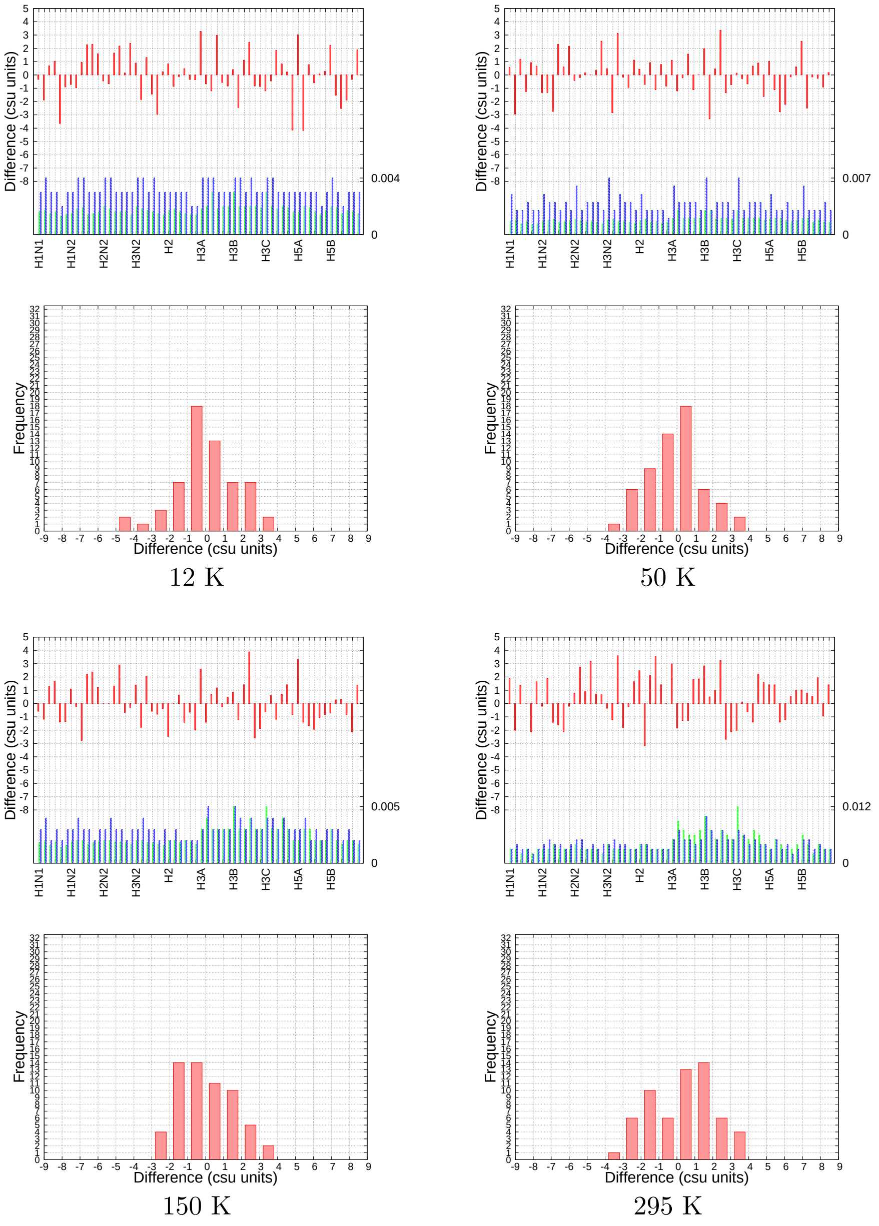

Figure S32: Hydrogen atom ADPs in Å^2, HF/cc-pVTZ

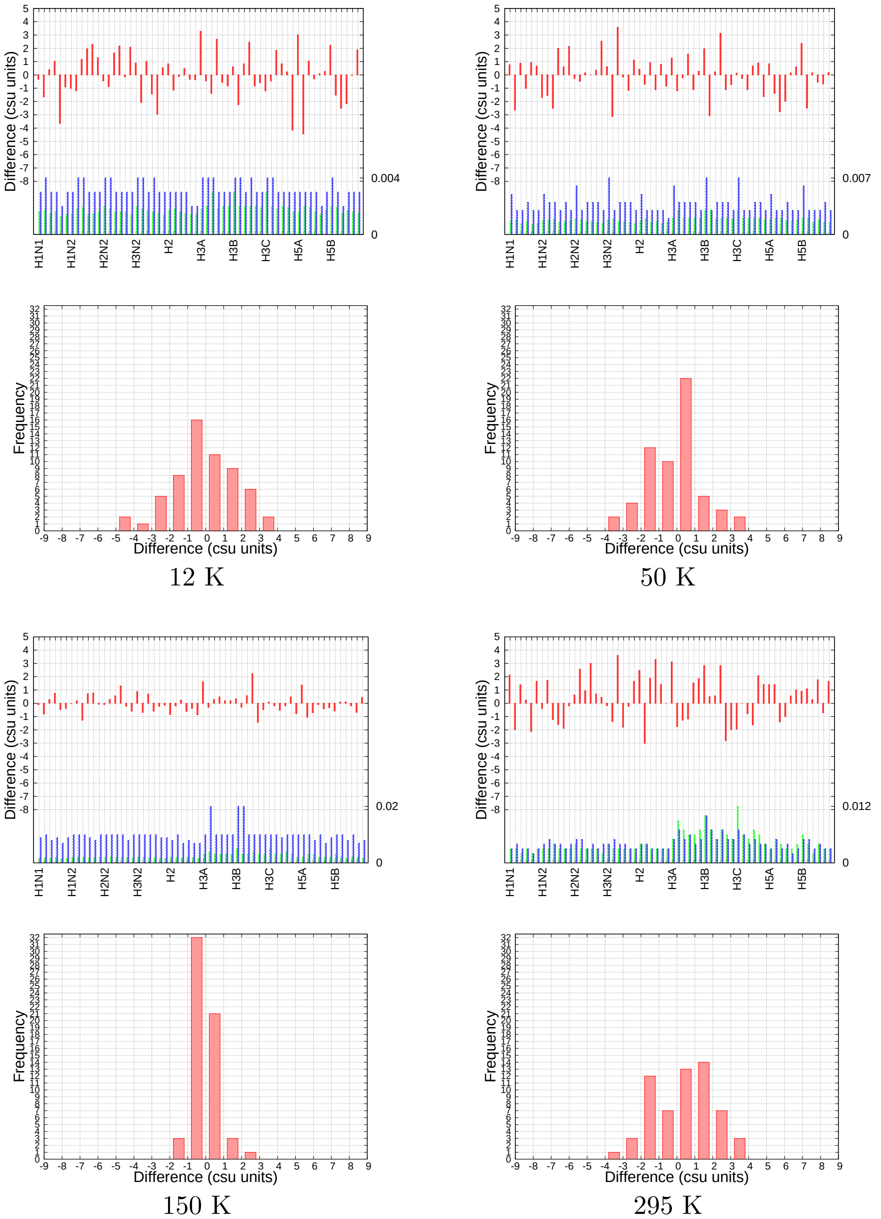

Figure S33: Hydrogen atom ADPs in Å^2, HF/cc-pVQZ

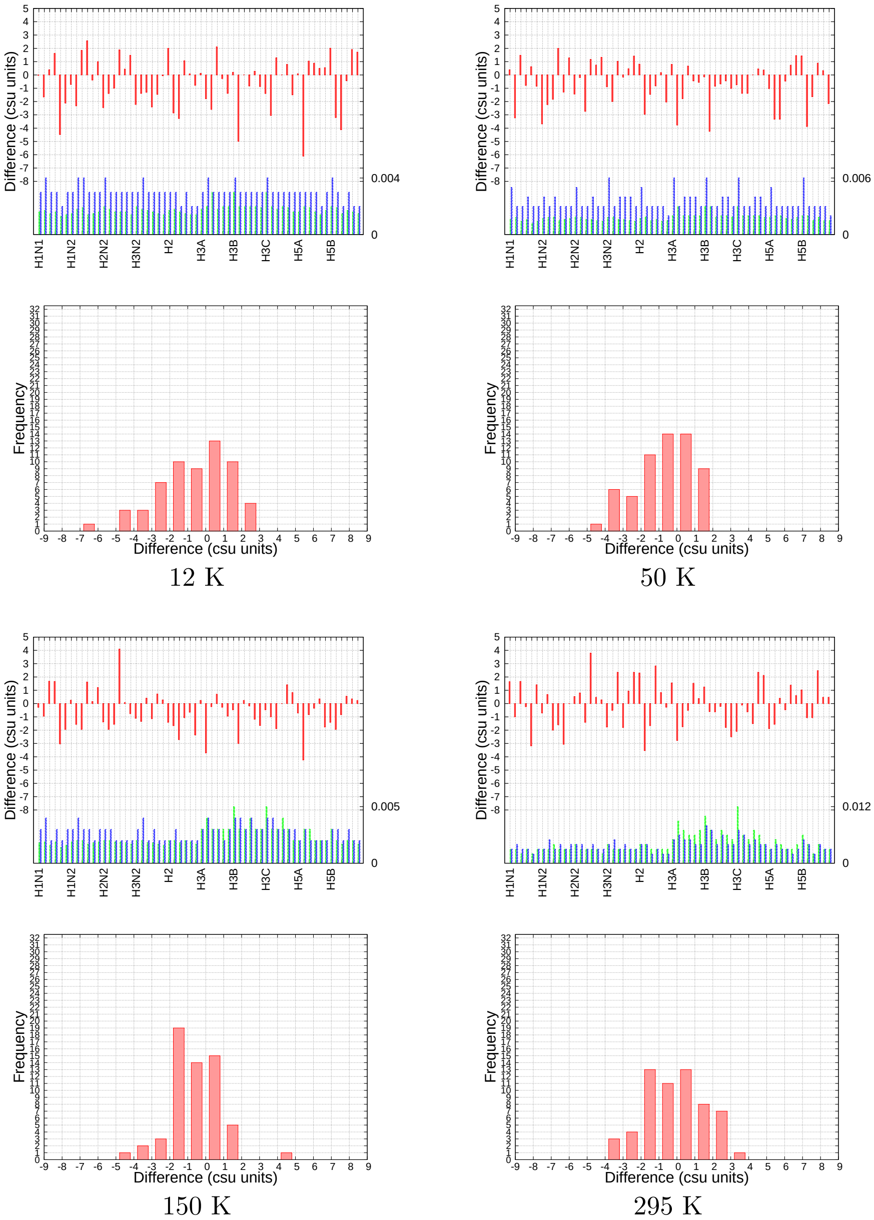

Figure S34: Hydrogen atom ADPs in Å^2, BLYP/cc-pVDZ

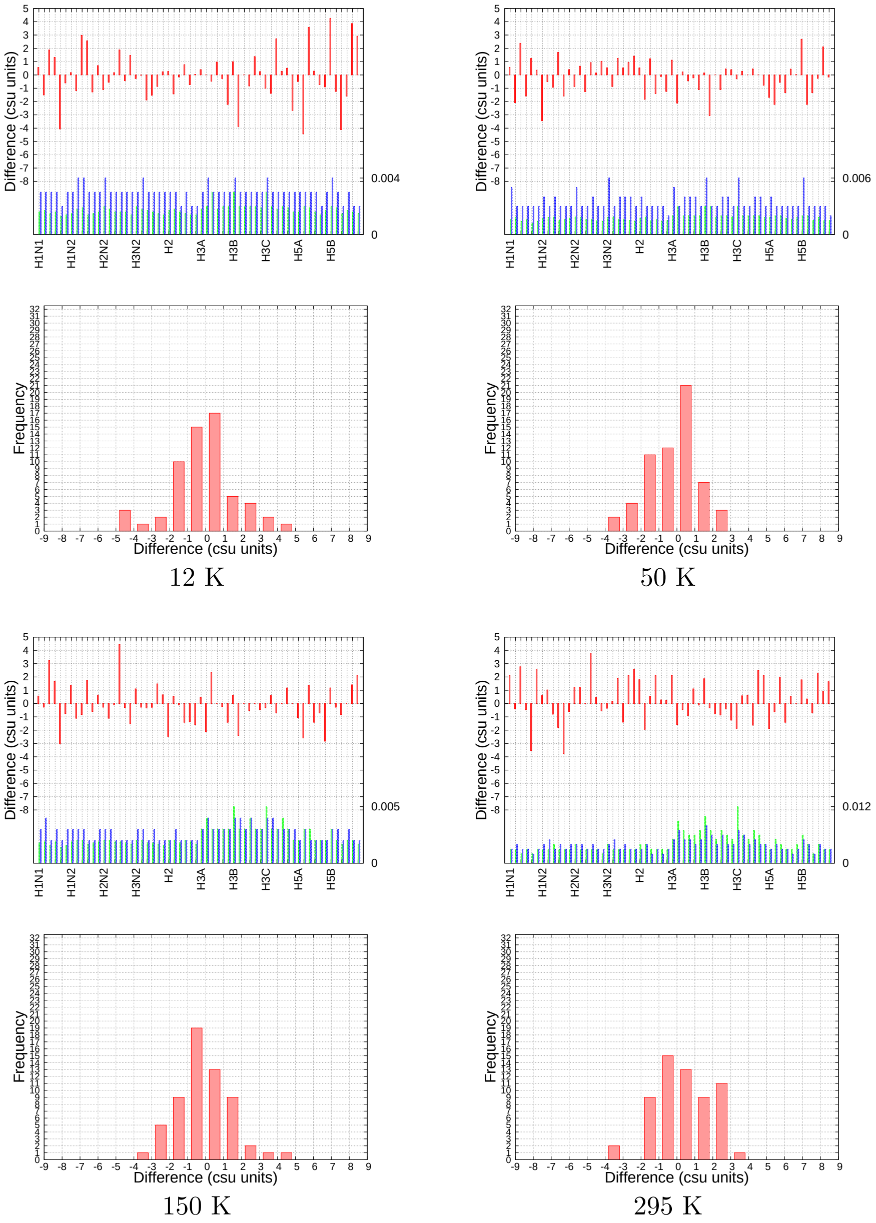

Figure S35: Hydrogen atom ADPs in Å^2, BLYP/cc-pVTZ

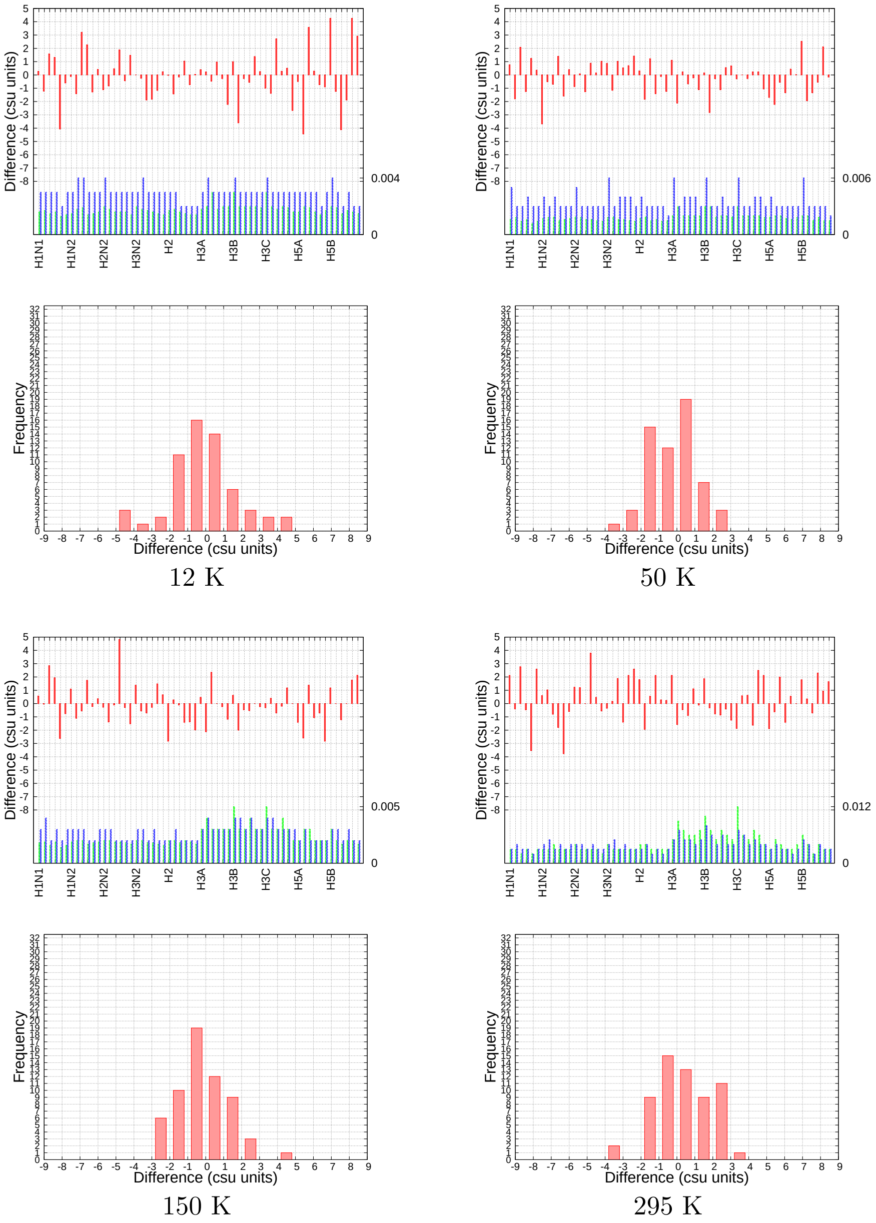

Figure S36: Hydrogen atom ADPs in Å^2, BLYP/cc-pVQZ

14 Hirshfeld rigid-bond test

Table S26: Differences of the mean square displacement amplitudes (DMSDA; first atom minus second atom per bond) along the bond vector in Å^2 for ADPs derived from HARs at BLYP/cc-pVTZ level of theory and from neutron diffraction.

T	Method	C1-O1	C1-O2	C4-O3	C2-N1	C4-N1
12 K	HAR	0.0003(1)	0.0002(1)	0.0004(1)	0.0001(1)	0.0001(1)
	neutron	0.0002(9)	-0.0007(9)	0.0004(9)	0.0007(7)	-0.0004(7)
50 K	HAR	0.0003(2)	0.0004(2)	0.0003(2)	-0.0001(2)	0.0002(2)
	neutron	0.0012(10)	0.0005(10)	0.0018(10)	-0.0010(8)	-0.0018(8)
150 K	HAR	0.0000(1)	0.0001(1)	-0.0001(1)	0.0000(1)	0.0003(1)
	neutron	-0.0014(12)	-0.0013(12)	0.0006(11)	0.0008(10)	0.0005(9)
295 K	HAR	0.0001(2)	-0.0002(2)	-0.0001(2)	-0.0002(2)	0.0003(2)
	neutron	-0.0025(20)	-0.0040(19)	0.0013(19)	-0.0004(14)	-0.0008(14)

T	Method	C5-N2	C1-C2	C2-C3	C4-C5
12 K	HAR	0.0005(1)	0.0000(1)	-0.0004(1)	-0.0005(1)
	neutron	-0.0002(8)	-0.0016(8)	-0.0028(9)	-0.0016(9)
50 K	HAR	0.0004(2)	-0.0003(2)	-0.0004(2)	-0.0001(2)
	neutron	-0.0013(8)	-0.0018(9)	-0.0001(10)	-0.0012(9)
150 K	HAR	0.0006(1)	-0.0003(1)	-0.0004(2)	-0.0004(1)
	neutron	-0.0011(10)	-0.0002(11)	-0.0006(13)	-0.0015(10)
295 K	HAR	0.0005(2)	-0.0002(2)	-0.0013(3)	-0.0007(2)
	neutron	-0.0019(15)	-0.0017(16)	0.0011(24)	0.0027(16)

T	Method	N1-H1N1	N2-H1N2	N2-H2N2	N2-H3N2	C2-H2
12 K	HAR	-0.0021(30)	-0.0116(37)	-0.0115(33)	-0.0007(33)	-0.0075(27)
	neutron	-0.0059(17)	-0.0040(18)	-0.0052(19)	-0.0043(19)	-0.0052(18)
50 K	HAR	-0.0150(37)	-0.0001(37)	-0.0115(37)	-0.0082(43)	-0.0029(33)
	neutron	-0.0038(17)	-0.0077(19)	-0.0072(18)	-0.0076(18)	-0.0046(18)
150 K	HAR	-0.0062(30)	-0.0132(30)	-0.0057(27)	-0.0039(27)	-0.0024(23)
	neutron	-0.0065(19)	-0.0038(20)	-0.0095(20)	-0.0051(20)	-0.0105(21)
295 K	HAR	-0.0019(33)	-0.0070(40)	-0.0077(37)	-0.0023(40)	-0.0127(30)
	neutron	-0.0019(28)	-0.0111(35)	-0.0076(35)	-0.0093(35)	0.0005(38)

T	Method	C3-H3A	C3-H3B	C3-H3C	C5-H5A	C5-H5B
12 K	HAR	-0.0071(33)	-0.0025(33)	-0.0024(33)	-0.0012(30)	-0.0094(30)
	neutron	-0.0059(24)	-0.0080(24)	-0.0063(24)	-0.0029(20)	-0.0085(19)
50 K	HAR	-0.0116(40)	-0.0022(40)	-0.0051(40)	0.0004(33)	-0.0032(40)
	neutron	-0.0084(24)	-0.0073(27)	-0.0064(24)	-0.0075(21)	-0.0088(20)
150 K	HAR	-0.0005(33)	-0.0055(37)	-0.0071(37)	-0.0001(23)	-0.0026(27)
	neutron	-0.0037(35)	-0.0066(38)	-0.0066(38)	-0.0059(28)	-0.0084(24)
295 K	HAR	-0.0100(57)	-0.0038(60)	-0.0154(57)	-0.0068(33)	-0.0054(37)
	neutron	-0.0052(73)	-0.0023(76)	0.0006(79)	-0.0023(42)	-0.0104(42)
