# Supplementary material for: Hirshfeld atom refinement
Source: IUCrJ. 2014 Aug 29;1(Pt 5):361–79. doi: 10.1107/S2052252514014845 (PMC4174878; doi:10.1107/S2052252514014845)

checkCIF/PLATON report

You have not supplied any structure factors. As a result the full set of tests cannot be run.

THIS REPORT IS FOR GUIDANCE ONLY. IF USED AS PART OF A REVIEW PROCEDURE FOR PUBLICATION, IT SHOULD NOT REPLACE THE EXPERTISE OF AN EXPERIENCED CRYSTALLOGRAPHIC REFEREE.

No syntax errors found. CIF dictionary Interpreting this report

Datablock: ga_12K_BLYP_cc-pVQZ

Bond precision: C-C = 0.0004 A Wavelength=0.52590

Cell:	a=7.4583(4)	b=9.4892(7)	c=9.7301(6)
	alpha=90	beta=90	gamma=90
Temperature:	12 K		

	Calculated	Reported
Volume	688.63(8)	688.63(8)
Space group	P 21 21 21	P 21 21 21
Hall group	P 2ac 2ab	P 2ac 2ab
Moiety formula	C5 H10 N2 O3	C5 H10 N2 O3
Sum formula	C5 H10 N2 O3	C5 H10 N2 O3
Mr	146.15	146.15
Dx,g cm-3	1.410	1.410
Z	4	4
Mu (mm-1)	0.063	0.071
F000	312.0	312.0
F000’	312.03	
h,k,lmax	11,14,14	11,14,14
Nref	2554[1481]	2474
Tmin,Tmax		
Tmin’		

Correction method= Not given

Data completeness= 1.67/0.97 Theta(max)= 23.640

R(reflections)= 0.0188(2380) wR2(reflections)= wR= 0.0231(8078)

S = 1.101 Npar= Npar = 181

The following ALERTS were generated. Each ALERT has the format
test-name_ALERT_alert-type_alert-level.
Click on the hyperlinks for more details of the test.

Alert level A

PLAT211_ALERT_2_A ADP of Atom H5B is N.P.D. or (nearly) 2D ... Please Check

Author Response: These are the values from the HAR refinement which is the subject of the paper; so the value is intentionally left. Please note this is a H ADP and overall agreement of these H ADPs with those derived from neutron measurements is within two combined standard uncertainty units.

Alert level C

CELLK01_ALERT_1_C Check that the cell measurement temperature is in Kelvin.
Value of measurement temperature given = 12.000
PLAT213_ALERT_2_C Atom H1N1 has ADP max/min Ratio ..... 3.6 prolat
PLAT213_ALERT_2_C Atom H3C has ADP max/min Ratio ..... 3.3 oblate
PLAT353_ALERT_3_C Long N-H (N0.87,N1.01A) N1 - H1N1 ... 1.01 Ang.
PLAT353_ALERT_3_C Long N-H (N0.87,N1.01A) N2 - H1N2 ... 1.04 Ang.
PLAT353_ALERT_3_C Long N-H (N0.87,N1.01A) N2 - H2N2 ... 1.04 Ang.
PLAT353_ALERT_3_C Long N-H (N0.87,N1.01A) N2 - H3N2 ... 1.03 Ang.
PLAT731_ALERT_1_C Bond Calc 1.5300(5), Rep 1.5300(2) ...... 3 su-Rat
C2 -C3 1.555 1.555 # 12

Alert level G

ABSMU01_ALERT_1_G Calculation of _exptl_absorpt_correction_mu
not performed for this radiation type.
PLAT720_ALERT_4_G Number of Unusual/Non-Standard Labels .... 4 Note
PLAT791_ALERT_4_G The Model has Chirality at C2 .... S Verify
PLAT808_ALERT_5_G No Parseable SHELXL Style Weighting Scheme Found Please Check
PLAT980_ALERT_1_G No Anomalous Scattering Factors Found in CIF ... Please Check

---

1 **ALERT level A** = Most likely a serious problem - resolve or explain
0 **ALERT level B** = A potentially serious problem, consider carefully
8 **ALERT level C** = Check. Ensure it is not caused by an omission or oversight
5 **ALERT level G** = General information/check it is not something unexpected

4 ALERT type 1 CIF construction/syntax error, inconsistent or missing data
3 ALERT type 2 Indicator that the structure model may be wrong or deficient
4 ALERT type 3 Indicator that the structure quality may be low
2 ALERT type 4 Improvement, methodology, query or suggestion
1 ALERT type 5 Informative message, check

It is advisable to attempt to resolve as many as possible of the alerts in all categories. Often the minor alerts point to easily fixed oversights, errors and omissions in your CIF or refinement strategy, so attention to these fine details can be worthwhile. In order to resolve some of the more serious problems it may be necessary to carry out additional measurements or structure refinements. However, the purpose of your study may justify the reported deviations and the more serious of these should normally be commented upon in the discussion or experimental section of a paper or in the "special_details" fields of the CIF. checkCIF was carefully designed to identify outliers and unusual parameters, but every test has its limitations and alerts that are not important in a particular case may appear. Conversely, the absence of alerts does not guarantee there are no aspects of the results needing attention. It is up to the individual to critically assess their own results and, if necessary, seek expert advice.

Publication of your CIF in IUCr journals

A basic structural check has been run on your CIF. These basic checks will be run on all CIFs submitted for publication in IUCr journals (*Acta Crystallographica*, *Journal of Applied Crystallography*, *Journal of Synchrotron Radiation*); however, if you intend to submit to *Acta Crystallographica Section C* or *E*, you should make sure that full publication checks are run on the final version of your CIF prior to submission.

Publication of your CIF in other journals

Please refer to the *Notes for Authors* of the relevant journal for any special instructions relating to CIF submission.

PLATON version of 05/02/2014; check.def file version of 05/02/2014

Datablock ga_12K_BLYP_cc-pVQZ - ellipsoid plot

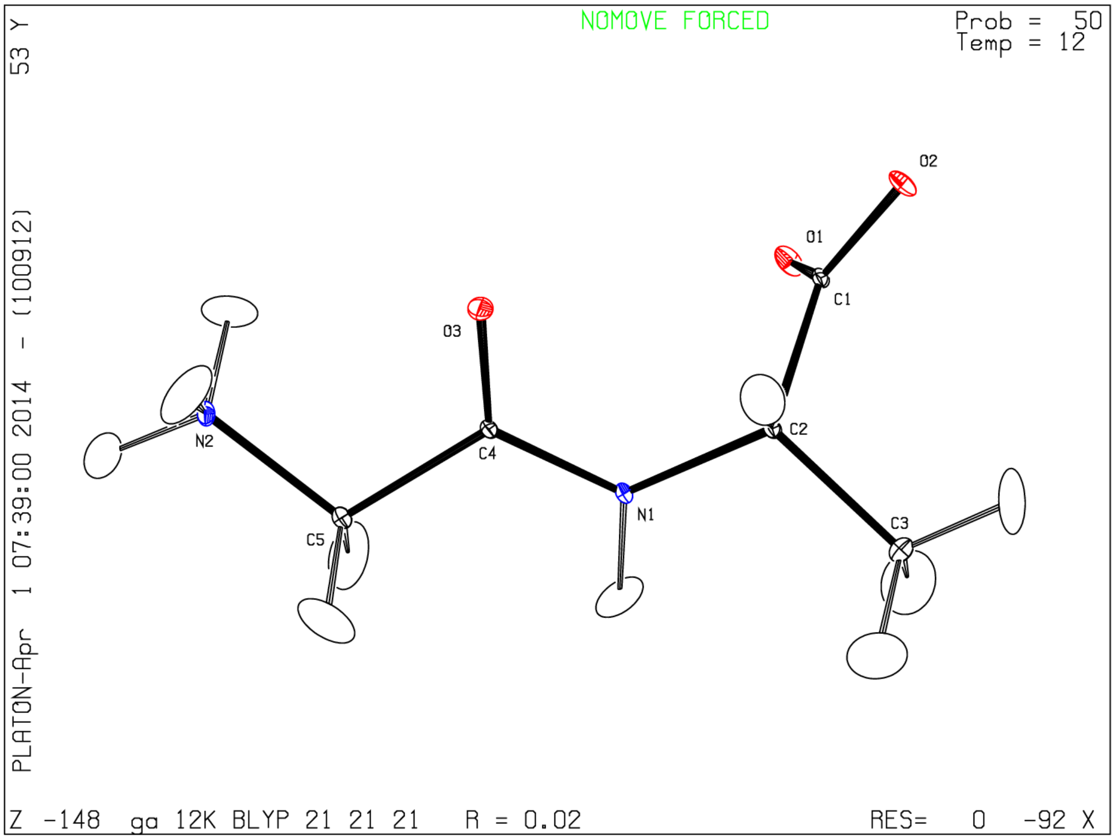

Supplement: Supplementary file 2 [file 12K.BLYP.cc-pVQZ.pdf › 12K.BLYP.cc-pVQZ.pdf]
