# Supplementary material for: Hirshfeld atom refinement
Source: IUCrJ. 2014 Aug 29;1(Pt 5):361–79. doi: 10.1107/S2052252514014845 (PMC4174878; doi:10.1107/S2052252514014845)

checkCIF/PLATON report

You have not supplied any structure factors. As a result the full set of tests cannot be run.

THIS REPORT IS FOR GUIDANCE ONLY. IF USED AS PART OF A REVIEW PROCEDURE FOR PUBLICATION, IT SHOULD NOT REPLACE THE EXPERTISE OF AN EXPERIENCED CRYSTALLOGRAPHIC REFEREE.

No syntax errors found. CIF dictionary Interpreting this report

Datablock: NH3HAR

```
Bond precision:  = 0.0000 A             Wavelength=0.71073

Cell:               a=5.1305(8)      b=5.1305(8)      c=5.1305(8)
                    alpha=90         beta=90          gamma=90
Temperature:        160 K

                Calculated                 Reported
Volume          135.05(6)                  135.05(6)
Space group     P 21 3                     P 21 3
Hall group      P 2ac 2ab 3                P 2ac 2ab 3
Moiety formula  H3 N                       H3 N
Sum formula     H3 N                       H3 N1
Mr              17.03                      17.03
Dx,g cm-3       0.838                      0.838
Z               4                          4
Mu (mm-1)       0.064                      0.064
F000            40.0                       40.0
F000'           40.00
h,k,lmax        7,7,7
Nref            130[ 83]
Tmin,Tmax
Tmin'

Correction method= Not given

Data completeness= 0.00/0.00      Theta(max)=

R(reflections)= 0.0092( 88)       wR2(reflections)= wR= 0.0074( 88)

S = 0.600                  Npar= Npar = 13
```

The following ALERTS were generated. Each ALERT has the format
test-name_ALERT_alert-type_alert-level.
Click on the hyperlinks for more details of the test.

Alert level B

```
PLAT049_ALERT_1_B Calculated Density less than 1.0 gcm-3 .........     0.8378
```

Alert level C

```
GOODF01_ALERT_2_C  The least squares goodness of fit parameter lies
            outside the range 0.80 <> 2.00
            Goodness of fit given =      0.600
PLAT029_ALERT_3_C _diffrn_measured_fraction_theta_full Low .......      0.962 Note
PLAT053_ALERT_1_C Minimum Crystal Dimension Missing (or Error) ...     Please Check
PLAT054_ALERT_1_C Medium  Crystal Dimension Missing (or Error) ...     Please Check
PLAT055_ALERT_1_C Maximum Crystal Dimension Missing (or Error) ...     Please Check
PLAT089_ALERT_3_C Poor Data / Parameter Ratio (Zmax < 18) ........       6.38 Note
```

Alert level G

```
PLAT005_ALERT_5_G No _iucr_refine_instructions_details  in the CIF     Please Do !
PLAT194_ALERT_1_G Missing _cell_measurement_reflns_used datum ....     Please Do !
PLAT195_ALERT_1_G Missing _cell_measurement_theta_max   datum ....     Please Do !
PLAT196_ALERT_1_G Missing _cell_measurement_theta_min   datum ....     Please Do !
PLAT808_ALERT_5_G No Parseable SHELXL Style Weighting Scheme Found     Please Check
PLAT980_ALERT_1_G No Anomalous Scattering Factors Found in CIF ...     Please Check
```

---

0 **ALERT level A** = Most likely a serious problem - resolve or explain
1 **ALERT level B** = A potentially serious problem, consider carefully
6 **ALERT level C** = Check. Ensure it is not caused by an omission or oversight
6 **ALERT level G** = General information/check it is not something unexpected

8 ALERT type 1 CIF construction/syntax error, inconsistent or missing data
1 ALERT type 2 Indicator that the structure model may be wrong or deficient
2 ALERT type 3 Indicator that the structure quality may be low
0 ALERT type 4 Improvement, methodology, query or suggestion
2 ALERT type 5 Informative message, check

It is advisable to attempt to resolve as many as possible of the alerts in all categories. Often the minor alerts point to easily fixed oversights, errors and omissions in your CIF or refinement strategy, so attention to these fine details can be worthwhile. In order to resolve some of the more serious problems it may be necessary to carry out additional measurements or structure refinements. However, the purpose of your study may justify the reported deviations and the more serious of these should normally be commented upon in the discussion or experimental section of a paper or in the "special_details" fields of the CIF. checkCIF was carefully designed to identify outliers and unusual parameters, but every test has its limitations and alerts that are not important in a particular case may appear. Conversely, the absence of alerts does not guarantee there are no aspects of the results needing attention. It is up to the individual to critically assess their own results and, if necessary, seek expert advice.

Publication of your CIF in IUCr journals

A basic structural check has been run on your CIF. These basic checks will be run on all CIFs submitted for publication in IUCr journals (*Acta Crystallographica*, *Journal of Applied Crystallography*, *Journal of Synchrotron Radiation*); however, if you intend to submit to *Acta Crystallographica Section C* or *E*, you should make sure that full publication checks are run on the final version of your CIF prior to submission.

Publication of your CIF in other journals

Please refer to the *Notes for Authors* of the relevant journal for any special instructions relating to CIF submission.

PLATON version of 05/02/2014; check.def file version of 05/02/2014

Datablock NH3HAR - ellipsoid plot

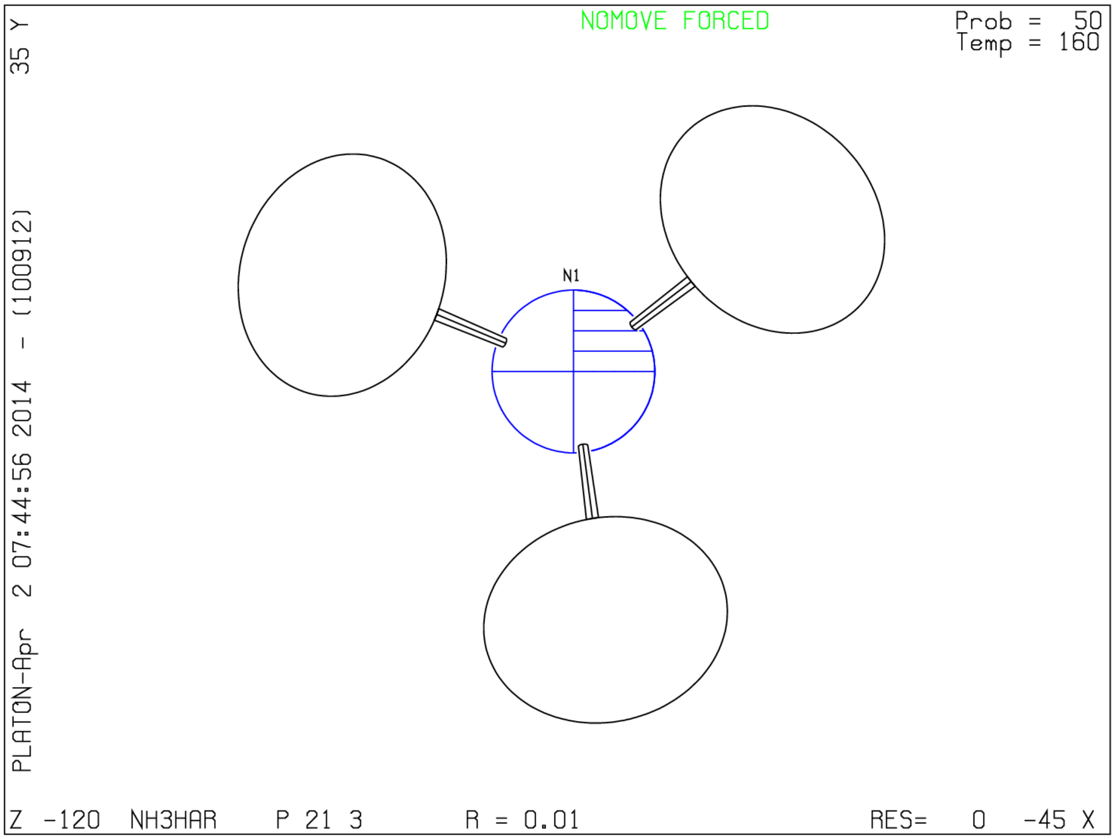

Supplement: Supplementary file 2 [file m-01-00361-sup2.zip › ../NH3_HAR.pdf]
